# Impact of Statin Therapy on the Risk of Stroke Recurrence, Mortality, and Dementia After Ischemic Stroke (ISMARDD Study): A Comprehensive Meta-Analysis

**DOI:** 10.3390/neurolint17110176

**Published:** 2025-11-01

**Authors:** Muskaan Gupta, Kevin J. Spring, Roy G. Beran, Sonu Bhaskar

**Affiliations:** 1 Global Health Neurology Lab, Sydney, NSW 2150, Australia; 2 UNSW Medicine and Health, University of New South Wales (UNSW), South West Sydney Clinical Campuses, Sydney, NSW 2170, Australia; 3 NSW Brain Clot Bank, NSW Health Pathology, Sydney, NSW 2170, Australia; 4 Medical Oncology Group, Ingham Institute for Applied Medical Research, Sydney, NSW 2751, Australia; 5 School of Medicine, Western Sydney University, Sydney, NSW 2000, Australia; 6 Griffith Health, School of Medicine and Dentistry, Griffith University, Southport, QLD 4215, Australia; 7 Ingham Institute for Applied Medical Research, Clinical Sciences Stream, Liverpool, NSW 2170, Australia; 8 Department of Neurology & Neurophysiology, Liverpool Hospital and South West Sydney Local Health District, Liverpool, NSW 2150, Australia; 9 Division of Cerebrovascular Medicine and Neurology, Department of Neurology, National Cerebral and Cardiovascular Center (NCVC), Suita 564-8565, Osaka, Japan

**Keywords:** stroke, statins, secondary prevention, post-stroke dementia, meta-analysis, ISMARDD study, cerebrovascular disorders

## Abstract

Background: Ischemic stroke (IS) remains a leading global cause of mortality, recurrence, and long-term disability, with survivors also at risk of post-stroke dementia (PSD) and cognitive impairment (PSCI). The precise impact of statin therapy across different IS populations, including those with cardioembolic/atrial fibrillation (CE/AF) strokes and patients with low-baseline low-density lipoprotein (LDL) cholesterol, remains unclear, as does the influence of statin timing, intensity, type, and solubility. Methods: We conducted the Impact of Statin Therapy on the Risk of Stroke Recurrence, Mortality, and Dementia After Ischemic Stroke (ISMARDD) meta-analysis, synthesizing evidence from 51 studies (*n* = 521,126), to evaluate the association between post-stroke statin therapy and key outcomes: all-cause mortality, stroke recurrence, cognition, and C-reactive protein (CRP). PSD was defined as new, persistent cognitive decline meeting standard diagnostic criteria, and PSCI as measurable but sub-threshold cognitive deficits. Random-effects models were used, and certainty was assessed with the Grading of Recommendations Assessment, Development, and Evaluation (GRADE) framework. Results: Statin therapy significantly reduced all-cause mortality within 3 months (OR 0.32), at 1 year (OR 0.35), and beyond 1 year (OR 0.56). Stroke recurrence was modestly reduced both within 1 year (OR 0.77) and after 1 year (OR 0.76). Statin use was associated with a lower risk of PSD (OR 0.74) but not PSCI overall. Benefits extended to CE/AF-related strokes and patients with low-baseline LDL cholesterol, both showing significantly lower mortality with statin use. Early initiation (<24 h) was linked with reduced recurrence, though effects of statin intensity, type, and solubility were inconsistent. Statins also significantly reduced CRP levels, underscoring anti-inflammatory and pleiotropic mechanisms. Conclusions: The ISMARDD study demonstrates that statins confer survival benefit and selective cognitive protection (notably reduced PSD risk) after ischemic stroke, with modest recurrence benefit, supporting their broad use in secondary prevention. These findings highlight the need for precision-guided approaches tailored to stroke subtype, pharmacogenomics, and treatment timing to optimize therapeutic outcomes.

## 1. Introduction

Ischemic stroke (IS) remains a leading global cause of death and long-term disability, with rising incidence particularly among ageing populations [1]. Despite advances in acute stroke interventions such as thrombolysis and mechanical thrombectomy, many survivors remain at elevated risk of recurrent stroke, cognitive impairments, and death [2,3]. While this epidemiological burden is well established, the critical question for clinical pharmacology is how pharmacological interventions, particularly statins, modify these risks beyond lipid lowering.

Statins, or 3-hydroxy-3-methylglutaryl-coenzyme A (HMG-CoA) reductase inhibitors, are central to cardiovascular and cerebrovascular risk reduction due to their low-density lipoprotein (LDL) cholesterol-lowering effects [4]. Beyond lipid modulation, accumulating evidence supports statins’ pleiotropic actions, including anti-inflammatory, endothelial-stabilizing, and neuroprotective effects [5], which may be especially relevant in the complex post-ischemic environment. Their ability to lower C-reactive protein (CRP) provides a biomarker-based link between statin exposure and anti-inflammatory benefit, suggesting mechanisms that could explain mortality and cognitive protection across diverse stroke subtypes.

The pivotal Stroke Prevention by Aggressive Reduction in Cholesterol Levels (SPARCL) trial demonstrated the efficacy of high-dose atorvastatin (80 mg daily) in reducing stroke recurrence in non-cardioembolic IS patients [6]. However, the SPARCL trial excluded patients with atrial fibrillation (AF), cardioembolic strokes (CE), and coronary artery disease (CAD)—populations that constitute a large proportion of real-world IS cases [6,7,8]. Current guideline recommendations, derived largely from SPARCL, therefore fail to fully reflect the heterogeneity of IS subtypes and comorbidities seen in practice, including CE/AF-related strokes and patients with low baseline LDL-cholesterol [9,10,11,12,13,14,15,16].

While previous meta-analyses have investigated the impact of statin therapy on stroke recurrence and mortality, they often have inconsistent inclusion criteria, limited subtype representation, and a lack of stratification by pharmacological parameters such as statin intensity, solubility, or timing of initiation [17,18]. Moreover, few reviews have systematically integrated these drug-related factors with outcomes such as post-stroke cognitive impairment (PSCI) (referred to as measurable but sub-threshold cognitive deficits within 3–6 months of the stroke) or dementia (PSD) (defined as persistent cognitive decline meeting standard diagnostic criteria (Diagnostic and Statistical Manual of Mental Disorders [DSM] or International Classification of Diseases [ICD]) within 6 months of stroke) [19]—conditions increasingly recognized as critical long-term sequelae of IS [2,20]. Incorporating CRP and pharmacogenomic considerations provides a translational bridge between statin pharmacology and clinical outcomes, directly informing precision prescribing.

The Impact of Statin Therapy on the Risk of Stroke Recurrence, Mortality, and Dementia After Ischemic Stroke (ISMARDD) study was undertaken to address these critical gaps. We conducted a comprehensive systematic review and meta-analysis to evaluate the effects of post-stroke statin therapy on all-cause mortality, stroke recurrence, and cognitive outcomes across a broad range of IS populations. We further examined how these outcomes vary by statin intensity, type, solubility, and timing of initiation, and assessed the impact on CRP levels as a mechanistic marker, to provide insight into their pleiotropic and anti-inflammatory mechanisms. By synthesizing data from over 500,000 patients, the ISMARDD study provides an updated, evidence-based foundation for optimizing statin therapy in post-stroke management and advancing precision-based prevention strategies.

### Objectives

This study aims to address the following research questions:

Primary Questions

(a) In the broader IS cohort:
  i. What is the prevalence of all-cause mortality, stroke recurrence and PSD/PSCI in statin users, nonusers and overall?
  ii. Is post-stroke statin use associated with all-cause mortality, stroke recurrence and PSD/PSCI?
(b) Variations in statin parameters:
  i. Is increasing statin intensity associated with all-cause mortality and stroke recurrence?
  ii. What is the prevalence of all-cause mortality and stroke recurrence by statin intensity, type, timing of initiation and solubility?

Secondary Questions

(a) In CE/AF-related IS subgroups:
  i. What is the prevalence of all-cause mortality and stroke recurrence in statin users, nonusers and overall?
  ii. Is post-stroke statin use associated with all-cause mortality and stroke recurrence?
(b) In IS patients with low baseline LDL-cholesterol:
  i. What is the prevalence of all-cause mortality in statin users, nonusers, and overall?
  ii. Is post-stroke statin use associated with all-cause mortality?
(c) Is post-stroke statin use associated with changes in CRP levels in IS patients?

## 2. Materials and Methods

### 2.1. Literature Search and Study Selection

A comprehensive literature search for studies published between January 2005 to June 2025 was conducted in PubMed, EMBASE, Scopus, Web of Science and Cochrane Library databases. Key terms in the search strategy included: “stroke”, “brain infarction”, “cerebrovascular event”, “cerebrovascular accident”, “statin” and “hmg-coa reductase inhibitor”. Searches were limited to human studies and those published in or translated to English. A detailed search strategy is available in Supplemental Table S1.

All identified titles and abstracts were initially screened and filtered using EndNote Version 21.5 (Clarivate Analytics, London, UK). The eligibility of remaining articles was thoroughly examined against the inclusion and exclusion criteria outlined below. Additional relevant studies were identified by examining the reference list of systematic reviews and meta-analyses and via Google Scholar. These processes were conducted independently by two researchers, and any discrepancies were resolved through discussion.

**Figure 1:**
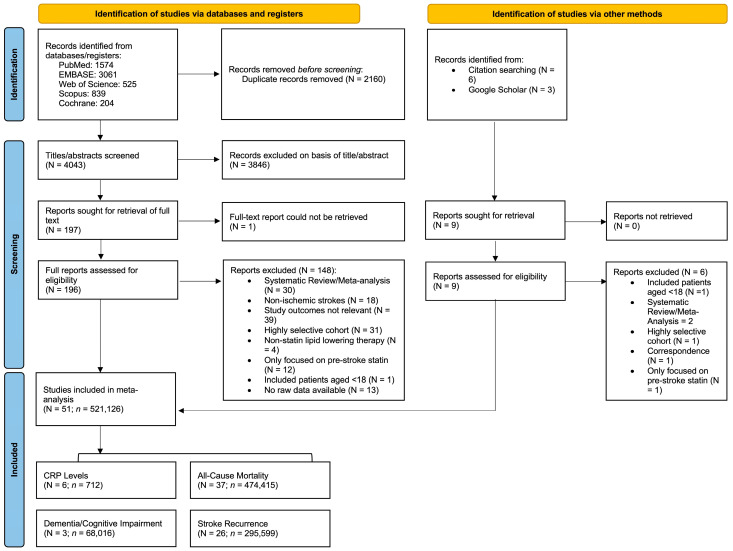
Preferred Reporting System for Systematic Reviews and Meta-Analyses (PRISMA) Flowchart showing the studies included in the meta-analysis. Abbreviations: N, number of studies; *n*, number of patients; CRP, C-reactive protein.

The systematic flow of the literature search and study selection is depicted in the Preferred Reporting Items for Systematic Reviews and Meta-Analyses (PRISMA) flowchart (Figure 1). This report aligns with the PRISMA 2020 and the Meta-analysis Of Observational Studies in Epidemiology (MOOSE) checklists, which are presented in Supplemental Tables S2 and S3. This study was also registered in Open Science, registration number “mqnk5” (https://osf.io/mqnk5/ [accessed on 20 July 2025]).

### 2.2. Inclusion and Exclusion Criteria

Studies were included in this meta-analysis if they satisfied the following criteria:

(a) participants aged 18 years or older;
(b) patients receiving statin therapy after IS (either pre-stroke statin continued or newly initiated statin after stroke);
(c) studies recruiting patients with all IS subtypes, or those focused on CE/AF-related strokes or patients with low baseline LDL-cholesterol;
(d) included data on the outcomes of PSD/PSCI, stroke recurrence, all-cause mortality; or reported mean and standard deviation or median and interquartile ranges of CRP levels;
(e) English language publications or translated to English; and
(f) sample size of at least 20 patients.

Studies were excluded if:

(a) included hemorrhagic stroke patients;
(b) restricted to highly select cohorts, such as those with cancer or excluded key IS subtypes, such as those focused only on non-CE strokes;
(c) did not contain raw data for outcomes;
(d) only reported on pre-stroke statin use;
(e) case reports, small case series, or studies with insufficient sample size;
(f) systematic reviews and meta-analyses;
(g) non-English or not translated.

### 2.3. Data Extraction

An Excel spreadsheet was utilized to extract the following key data from the selected studies:

1. Study demographics—author, publication year, country, study design, cohort size.
2. Control and intervention characteristics—statin type, dose, and timing of initiation.
3. Patient demographics—age, sex, National Institute of Health Stroke Scale (NIHSS) score, IS subtype according to Trial of Org 10172 in Acute Stroke Treatment (TOAST) classification, comorbidities (hypertension, diabetes mellitus, atrial fibrillation, coronary artery disease, dyslipidemia, previous stroke) and lifestyle factors (smoking and alcohol consumption).
4. Clinical outcomes—all-cause mortality (within 3 months, 1 year and after 1 year), stroke recurrence (within and after 1 year), any PSD/PSCI diagnosis and CRP levels (within 3–7 days and after 7 days).

When required, Wan et al.’s method was employed to estimate means and standard deviations (SDs) from medians and interquartile ranges (IQR) [21]. Statin solubility was determined based on the statin characteristics defined by Climent et al. [22]. Statin intensity was based on the American College of Cardiology & American Heart Association (ACC/AHA) Classification of Intensity [23].

### 2.4. Methodological Quality Assessment of Included Studies

The methodological quality of the included studies was assessed using the Modified Jadad Analysis (Supplemental Table S4). We also assessed non-randomized studies with the Risk of Bias In Non-randomized Studies of Interventions (ROBINS-I) tool and randomized controlled trials (RCTs) with the Risk of Bias (RoB 2) tool (Supplemental Tables S5 and S6); two reviewers assessed each study independently. The potential risk of bias due to funding was also evaluated based on the declaration of funding sources and conflicts of interest reported in each individual study (Supplemental Table S7).

### 2.5. Statistical Analyses

Statistical analyses were conducted using STATA version 13.0 (StataCorp, College Station, TX, USA). Summary analyses of patient characteristics were determined from the patient demographic data extracted from the included studies. In the analysis of statin parameter variations, any study that specified a statin parameter (such as timing of initiation, type or dose) was included in the analyses. In some instances, the number of patients included in the analysis were fewer than the initial total of patients due to missing data or lack of follow-up. Where possible, analyses were stratified by time-period, to determine both short-term and long-term effects of statin therapy, as well as by study design.

#### 2.5.1. Prevalence Estimations

The “metaprop” package in STATA was used to perform a random-effects meta-analysis of proportions in studies to determine the pooled prevalence of the clinical outcomes in statin users and nonusers. The “cimethod(exact)” and “ftt” commands were used to obtain the 95% confidence intervals (CIs). Pooled prevalence estimates were calculated using all available studies, with no minimum number of studies required for inclusion in each analysis.

#### 2.5.2. Outcome Associations

The “metan” package in STATA was used to perform a DerSimonian and Laird (DL) random-effects meta-analysis and construct forest plots to determine the association between post-stroke statin use and clinical outcomes. The DL model was selected given its ability to account for heterogeneity between and within studies. A minimum of three studies were required to conduct association analyses.

The analysis involved the calculation of odds ratios (ORs) between post-stroke statin exposure and outcomes such as all-cause mortality, stroke recurrence and PSD/PSCI. For zero-event studies, we used a continuity correction of 0.5. An OR < 1.0 indicated lower odds of the adverse outcome. Standardized mean differences (SMDs) were also calculated to determine the association between post-stroke statin use and changes CRP levels. A negative SMD indicated lower CRP levels in the statin exposed group.

#### 2.5.3. Statistical Significance, Heterogeneity and Variance

Statistical significance was considered when the *p*-value was <0.05. Heterogeneity was quantified with τ^2^, I^2^, H^2^, and Cochran’s Q. Similarly to Shen et al., heterogeneity was considered low when I^2^ < 30%, moderate when I^2^ = 30–50%, substantial when I^2^ = 51–75%, and severe when I^2^ > 75% [24]. We explored heterogeneity sources through subgroup analyses by stroke subtype, statin parameters, and study design.

#### 2.5.4. Bias and Sensitivity Analyses

The “metaninf” package in STATA was used to conduct sensitivity analyses which assessed the impact on the pooled OR when individual studies were excluded. We assessed small-study effects using Egger’s and Peters’ tests and inspected funnel plots.

### 2.6. Certainty of Evidence Assessment

We assessed certainty of evidence for each primary outcome using the Grading of Recommendations Assessment, Development and Evaluation (GRADE) framework, considering risk of bias, inconsistency, indirectness, imprecision, and publication bias. Certainty ratings were presented as high, moderate, low, or very low. For each outcome, we provided the relative effect, assumed control risk, and calculated absolute effect estimates where baseline risk was available.

## 3. Results

### 3.1. Description of Included Studies

From 4052 identified articles, 206 full-text articles were assessed for eligibility. Of these, 144 were excluded for various reasons such as irrelevant study outcomes and highly selective or non-IS focused cohorts (Figure 1). A total number (N) of 51 studies, encompassing a total of (*n*) 521,126 patients, were included in this meta-analysis [25,26,27,28,29,30,31,32,33,34,35,36,37,38,39,40,41,42,43,44,45,46,47,48,49,50,51,52,53,54,55,56,57,58,59,60,61,62,63,64,65,66,67,68,69,70,71,72,73,74,75]. Among these, 31 studies were focused on all IS subtypes, of which 3 studies looked at PSD/PSCI [49,72,75], 21 looked at all-cause mortality [25,26,28,31,36,37,38,39,40,41,44,45,53,56,59,62,64,67,68,70,73], 12 looked at stroke recurrence [25,39,43,45,53,55,56,59,65,67,70,74], and 6 studies looked at CRP levels [26,29,57,61,65,74]. 10 studies were focused on CE/AF-related stroke, of which 8 looked at all-cause mortality [33,34,35,42,52,58,60,66] and 8 looked at stroke recurrence [33,34,35,54,58,60,66,69]. Three studies looked at all-cause mortality in IS patients with low baseline LDL-cholesterol [47,51,63]. Finally, 9 studies purely focused on statin parameters, including 6 studies on statin intensity [27,28,32,50,59,71], 2 on statin type [30,48], and 1 on statin timing of initiation [46]. Of the 51 studies included in the analysis, 22 studies provided data for multiple research questions.

Characteristics of the studies included in this meta-analysis—including study design, patient demographics as well as comorbidities and lifestyle factors—are detailed in Table 1 and Table 2, respectively. The meta-analysis incorporated studies from a diverse range of countries and age groups. Patients had high prevalence of cardiovascular risk factors and comorbidities such as hypertension, coronary artery disease and smoking. Variation in stroke treatment protocols was observed as the proportion of patients initiated on statin therapy after stroke ranged from 9.7% to 83.7%. A broad range of stroke severities were included, as evidenced by the wide spread of NIHSS scores.

### 3.2. Primary Analysis

#### 3.2.1. Impact of Post-Stroke Statin Use on Clinical Outcomes in Broader Ischemic Stroke Cohorts

##### Prevalence Estimations

All-Cause Mortality

The overall prevalence of all-cause mortality was 12% within 3 months (95% CI: [0.09, 0.16]; z = 11.67; *p* < 0.01; *n* = 11,233; N = 10) [28,31,36,37,41,56,62,64,70,73], 14% within 1 year (95% CI: [0.08, 0.21]; z = 7.82; *p* < 0.01; *n* = 84,567; N = 14) [25,26,28,31,36,37,40,41,44,56,62,64,70,73] and 23% after 1 year (95% CI: [0.07, 0.44]; z = 4.20; *p* = 0.01; *n* = 163,348; N = 8) [37,38,39,45,53,59,67,68] of IS (Table 3; Supplemental Figure S1). There was severe heterogeneity among these studies (I^2^ = 94.83%, 99.73% and 99.98%, respectively, for each analysis).

In statin users, the prevalence of all-cause mortality within 3 months was 8% (95% CI: [0.05, 0.11]; z = 8.38; *p* < 0.01; *n* = 5052; N = 10) compared to 18% (95% CI: [0.13, 0.24]; z = 10.38, *p* < 0.01; *n* = 6181; N = 10) in statin non-users) [28,31,36,37,41,56,62,64,70,73]. Within 1 year after stroke, statin users had a prevalence of 9% (95% CI: [0.06, 0.14]; z = 7.30, *p* < 0.01; *n* = 55,421; N = 14) compared to nonusers who had a 21% prevalence (95% CI: [0.14, 0.30]; z = 8.49; *p* < 0.01; *n* = 29,146; N = 14) [25,26,28,31,36,37,40,41,44,56,62,64,70,73]. After 1 year statin users had a prevalence of 19% (95% CI: [0.05, 0.41]; z = 3.58; *p* < 0.01; *n* = 97,171; N = 8) compared to 30% (95% CI: [0.14, 0.50]; z = 5.30, *p* < 0.01; *n* = 66,177; N = 8) in nonusers [37,38,39,45,53,59,67,68] (Table 3; Supplemental Figure S2).

Stroke Recurrence

The overall prevalence of stroke recurrence was 4% (95% CI: [0.02, 0.08]; z = 4.38; *p* < 0.01; *n* = 60,026; N = 5) [25,56,65,70,74] within 1 year and 17% (95% CI: [0.11, 0.24]; z = 9.02; *p* < 0.01; *n* = 144,993; N = 8) [25,39,43,45,53,55,59,67] after 1 year of IS (Table 3; Supplemental Figure S3). There was severe heterogeneity among these studies, (I^2^ = 81.38% and 99.86%, respectively, for each analysis).

In statin users, the prevalence of stroke recurrence within 1 year was 3% (95% CI: [0.01, 0.07]; z = 3.41; *p* < 0.01; *n* = 43,631; N = 5) compared to 5% (95% CI: [0.02, 0.10]; z = 4.59; *p* < 0.01; *n* = 16,395; N = 5) in nonusers [25,56,65,70,74]. After 1 year of IS, statin users had a prevalence of 13% (95% CI: [0.08, 0.20]; z = 7.69; *p* < 0.01; *n* = 101,244; N = 8) compared to 20% (95% CI: [0.13, 0.28]; z = 9.31; *p* < 0.01; *n* = 43,749; N = 8) in nonusers [25,39,43,45,53,55,59,67] (Table 3; Supplemental Figure S4).

Post-Stroke Dementia/Cognitive Impairment

The overall prevalence of PSD/PSCI was 18% (95% CI: [0.09, 0.30]; z = 6.16; *p* < 0.01; *n* = 68,016; N = 3) [49,72,75] (Table 3; Supplemental Figure S5). There were insufficient studies to calculate heterogeneity between the studies.

The prevalence of PSD/PSCI was 19% in both statin users (95% CI: [0.09, 0.31]; z = 5.67, *p* < 0.01; *n* = 46,831; N = 3) and nonusers (95% CI: [0.09, 0.31]; z = 6.05; *p* < 0.01; *n* = 21,185; N = 3) [49,72,75] (Table 3; Supplemental Figure S6).

##### Outcome Associations

All-Cause Mortality

**Figure 2:**
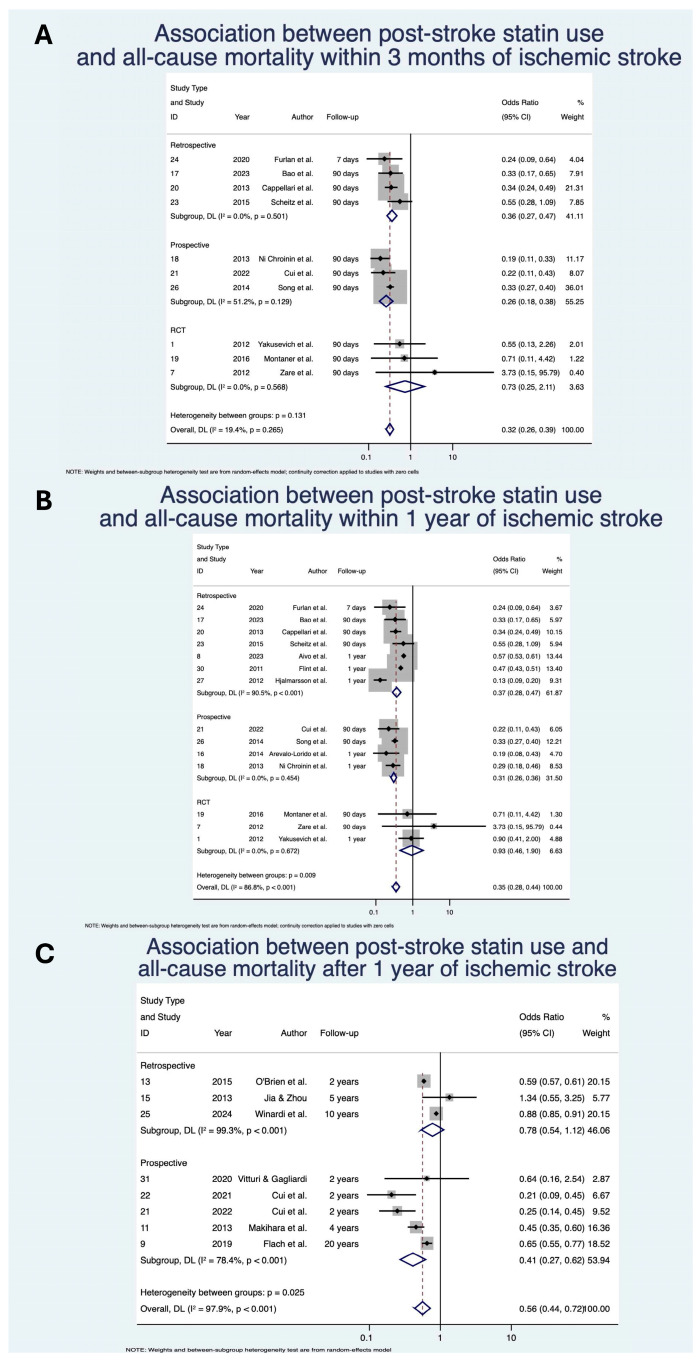
Association between post-stroke statin use and all-cause mortality (**A**) within 3 months of ischemic stroke; (**B**) within 1 year of ischemic stroke; (**C**) after 1 year of ischemic stroke [25,26,28,31,36,37,38,39,40,41,44,45,53,59,56,62,64,67,68,70,73]. Abbreviations: CI, confidence interval; DL, DerSimonian and Laird; *p*, *p*-value.

Statin use was associated with significantly decreased odds of all-cause mortality within 3 months with OR 0.32 (95% CI: [0.26, 0.39]; *p* < 0.01; z = −12.44; *n* = 11,233; N = 10) [28,31,36,37,41,56,62,64,70,73], within 1 year with OR 0.35 (95% CI: [0.28, 0.44]; *p* < 0.01; z = −9.30; *n* = 84,567; N = 14) [25,26,28,31,36,37,40,41,44,56,62,64,70,73] and after 1 year with OR 0.56 (95% CI: [0.44, 0.72]; *p* < 0.01; z = −4.51; *n* = 163,348; N = 8) [37,38,39,45,53,59,67,68] of IS (Table 4; Figure 2). The direction of effect remained consistent when stratified by study design, although was not significant for the RCT subgroups. Heterogeneity of studies was mixed, with low heterogeneity within the 3-month analysis (I^2^ = 19.4%), but severe heterogeneity for within 1-year (I^2^ = 86.8%) and after 1-year (I^2^ = 97.9%) analyses.

Stroke Recurrence

**Figure 3:**
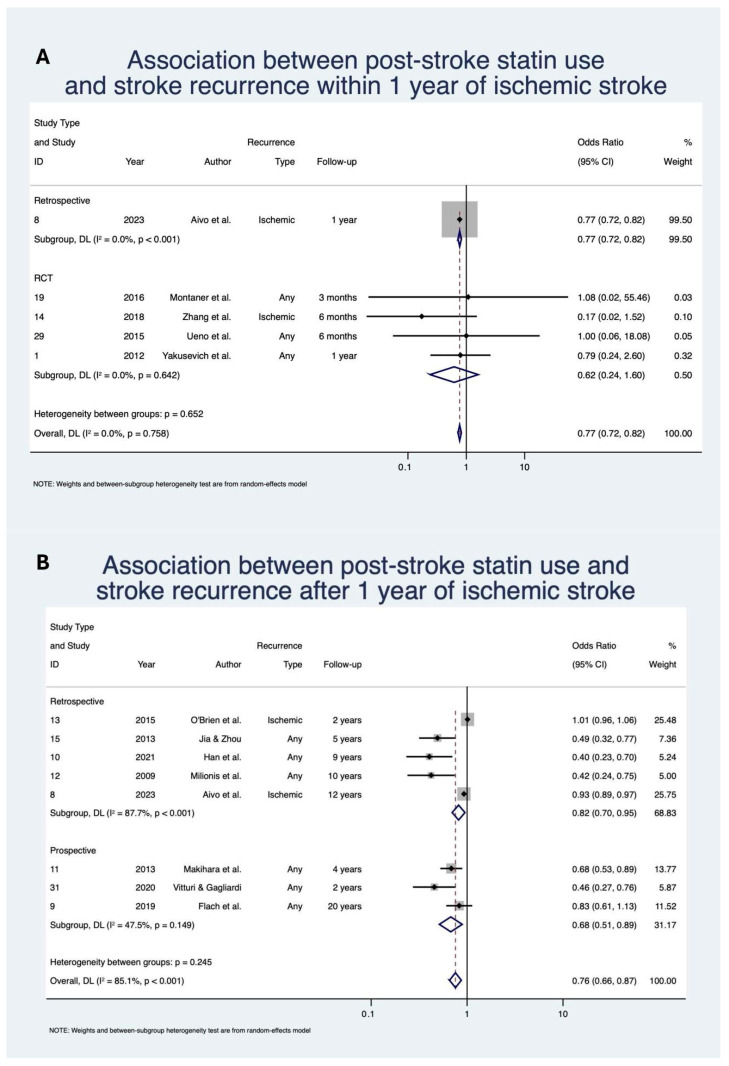
Association between post-stroke statin use and stroke recurrence: (**A**) within 1 year of ischemic stroke; (**B**) after 1 year of ischemic stroke [25,39,43,45,53,55,56,59,65,67,70,74]. Abbreviations: CI, confidence interval; DL, DerSimonian and Laird; *p*, *p*-value; Due to model limitations, studies with 0 events were replaced with 0.5 to allow for OR calculation.

Statin use was associated with significantly decreased odds of stroke recurrence within 1 year with OR 0.77 (95% CI: [0.72, 0.82], z = −7.64; *p* < 0.01; *n* = 60,026; N = 5) [25,56,65,70,74] and after 1 year with OR 0.76 (95% CI: [0.66, 0.87], z = −3.88; *p* < 0.01; *n* = 144,993; N = 8) [25,39,43,45,53,55,59,67] of IS (Table 4; Figure 3). The direction of effect remained consistent when stratified by study design, although was not significant for the RCT subgroup in the within 1 year analysis. Heterogeneity of studies was mixed, with low heterogeneity for the within 1 year analysis (I^2^ = 0.0%) but severe heterogeneity (I^2^ = 85.1%) for the post 1-year analysis.

Dementia/Cognitive Impairment

**Figure 4:**
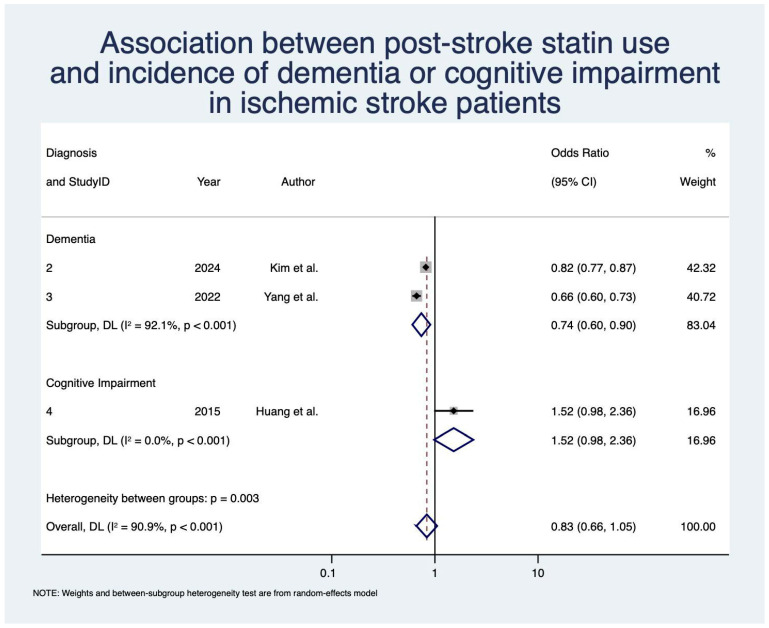
Association between post-stroke statin use and incidence of dementia/cognitive impairment in ischemic stroke patients [49,72,75]. Abbreviations: CI, confidence interval; DL, DerSimonian and Laird; *p*, *p*-value.

Statin use was not significantly associated with decreased odds of overall PSD or PSCI after IS with OR 0.83 (95% CI: [0.66, 1.05]; z = −1.54; *p* = 0.12; *n* = 68,016; N = 3) [49,72,75] (Table 4; Figure 4). When looking at PSD alone, statin use was associated with significantly decreased odds with OR 0.74 (95% CI: [0.60, 0.90]; z = −2.94; *p* < 0.01; *n* = 67,666; N = 2) [49,72]. Heterogeneity was severe for the overall analysis (I^2^ = 90.9%) and the PSD subgroup (I^2^ = 92.1%).

#### 3.2.2. Impact of Variations in Statin Parameters on Clinical Outcomes

Statin Timing

The prevalence of all-cause mortality in patients initiating statin within 24 h was 10% (95% CI: [0.06, 0.12]; z = 7.13; *p* < 0.01; *n* = 316; N = 3) [37,56,62] compared to 6% (95% CI: [0.03, 0.09]; z = 7.45; *p* < 0.01; *n* = 1169; N = 3) [31,32,36] in those initiating statin therapy after 24 h (Table 5; Supplemental Figure S7).

The prevalence of stroke recurrence, in patients initiating statin within 24 h, was 5% (95% CI: [0.01, 0.11]; z = 3.44; *p* < 0.01; *n* = 95; N = 2) [46,56] compared to 9% (95% CI: [0.06, 0.14]; z = 8.45; *p* < 0.01; *n* = 239; N = 2) [32,46] in those initiating statin therapy after 24 h (Table 5; Supplemental Figure S7).

Statin Type

The prevalence of all-cause mortality was lowest amongst rosuvastatin users (4%; 95% CI: [0.01, 0.07]; z = 4.55; *p* < 0.01; *n* = 6919; N = 2) [48,71] and highest amongst simvastatin users (10%; 95% CI: [0.06, 0.16]; z = 6.44; *p* < 0.01; *n* = 136; N = 2) [56,70] (Table 5; Supplemental Figure S8).

The prevalence of stroke recurrence was lowest amongst rosuvastatin users, though not statistically significant (2%; 95% CI: [0.00, 0.07]; z = 1.47; *p* = 0.14; *n* = 6681; N = 3) [30,48,65]. Stroke recurrence was highest amongst atorvastatin users (4%; 95% CI: [0.03, 0.05]; z = 10.93; *p* < 0.01; *n* = 37,080; N = 3) [30,32,48] (Table 5; Supplemental Figure S8).

Statin Solubility

The prevalence of all-cause mortality was similar between hydrophilic (6%, 95% CI: [0.05, 0.06]; z = 36.84; *p* < 0.01; *n* = 6919; N = 2) [48,71] and lipophilic (6%; 95% CI: [0.03, 0.10]; z = 5.88; *p* < 0.01; *n* = 37,181; N = 5) statin users [32,48,56,70,73] (Table 5; Supplemental Figure S9).

The prevalence of stroke recurrence was lower amongst hydrophilic statin users (2%; 95% CI: [0.00, 0.07]; z = 1.47; *p* < 0.01; *n* = 6681; N = 3) [30,48,65] compared to lipophilic (4%; 95% CI: [0.02, 0.06]; z = 6.37; *p* < 0.01; *n* = 37,216; N = 5) statin users [30,32,48,56,70] (Table 5; Supplemental Figure S9).

Statin Intensity

The prevalence of all-cause mortality was lowest in the moderate-high intensity statin group (4%; 95% CI: [0.02, 0.06]; z = 7.54; *p* < 0.01; *n* = 471; N = 2) [28,67] and was highest in the low-moderate intensity statin group (19%; 95% CI: [0.12, 0.28]; z = 8.31; *p* < 0.01; *n* = 21,001; N = 4) [28,37,38,59] (Table 5; Supplemental Figure S10).

The prevalence of stroke recurrence was lowest in the moderate intensity statin group (4%; 95% CI: [0.00, 0.10]; z = 2.40; *p* = 0.02; *n* = 205; N = 4) [32,56,65,70] and was highest in the moderate-high intensity statin group (17%; 95% CI: [0.13, 0.22]; z = 14.90; *p* < 0.01; *n* = 344; N = 1) [67]. The prevalence of stroke recurrence was similar between the low-moderate (10%; 95% CI: [0.10, 0.11]; z = 93.47; *p* = 0.01; *n* = 20,486; N = 1) [59] and high intensity (10%; 95% CI: [0.10, 0.11]; z = 55.10; *p* = 0.12; *n* = 9205; N = 2) [32,59] statin groups (Table 5; Supplemental Figure S10).

Increasing intensity of statin was not significantly associated with all-cause mortality with OR 0.92 (95% CI: [0.76, 1.12]; z = −0.80; *p* = 0.42; *n* = 103,236; N = 6) [27,28,32,50,59,71] or stroke recurrence with OR 1.10 (95% CI [0.88, 1.38]; z = −0.82; *p* = 0.41; *n* = 102,647; N = 4) [27,32,50,59] (Table 5; Supplemental Figure S11).

### 3.3. Secondary Analysis

#### 3.3.1. Impact of Post-Stroke Statin Use in Cardioembolic/Atrial Fibrillation-Related Strokes

##### Prevalence Estimations

All-Cause Mortality

The overall prevalence of all-cause mortality was 23% (95% CI: [0.18, 0.28]; z = 15.74; *p* < 0.01; *n* = 51,280; N = 3) [42,52,60] within 1 year and 10% (95% CI: [0.04, 0.17]; z = 4.94; *p* < 0.01; *n* = 24,805; N = 5) [33,34,35,58,66] after 1 year of cardioembolic or AF-related stroke (Supplemental Table S8; Supplemental Figure S12).

In statin users, the prevalence of all-cause mortality within 1 year was 19% (95% CI: [0.14, 0.24]; z = 12.97; *p* < 0.01; *n* = 7107; N = 3) compared to 30% (95% CI: [0.19, 0.42]; z = 8.66, *p* < 0.01; *n* = 44,373; N = 3) in nonusers [42,52,60]. This was again observed after 1 year with a prevalence of 5% (95% CI: [0.02, 0.10]; z = 4.07, *p* < 0.01; *n* = 9497; N = 5) in statin users compared to 10% (95% CI: [0.03, 0.20]; z = 3.95, *p* < 0.01; *n* = 14,591; N = 5) in nonusers [33,34,35,58,66] (Supplemental Table S8; Supplemental Figure S13).

Stroke Recurrence

The overall prevalence of stroke recurrence was 6% (95% CI: [0.05, 0.06]; z = 31.22; *p* < 0.01; *n* = 4630; N = 2) [54,60] within 1 year and 13% (95% CI: [0.10, 0.18]; z = 11.60; *p* < 0.01; *n* = 28,723; N = 6) [33,34,35,58,66,69] after 1 year of cardioembolic or AF-related stroke (Supplemental Table S8; Supplemental Figure S14).

In statin users, the prevalence of stroke recurrence within 1 year was 5% (95% CI: [0.04, 0.06]; z = 22.78, *p* < 0.01; *n* = 2761; N = 2) compared to 6% (95% CI: [0.05, 0.07]; z = 20.53, *p* < 0.01; *n* = 1869; N = 2) in nonusers [54,60]. 1 year after stroke, prevalence of stroke recurrence was 12% (95% CI: [0.08, 0.17]; z = 8.94; *p* < 0.01; *n* = 11,040; N = 6) among statin users compared to 13% (95% CI: [0.10, 0.16]; z = 13.35; *p* < 0.01; *n* = 17,683; N = 6) in nonusers [33,34,35,58,66,69] (Supplemental Table S8; Supplemental Figure S15).

##### Outcome Associations

All-Cause Mortality

Statin use was associated with significantly decreased odds of all-cause mortality both within 1 year with OR 0.57 (95% CI: [0.42, 0.78]; z = −3.49; *p* < 0.01; *n* = 51,280; N = 3) [42,52,60] and after 1 year with OR 0.47 (95% CI: [0.25, 0.88]; z = −2.37; *p* = 0.02; *n* = 24,805; N = 5) [33,34,35,58,66] in CE/AF-related stroke patients (Supplemental Table S9; Supplemental Figure S16). Heterogeneity for both analyses was severe, (I^2^ = 88.5% and 90.5%, respectively).

*Stroke* *Recurrence*

Due to insufficient data, association analyses were unable to be conducted for stroke recurrence within 1 year. However, statin use was not significantly associated with decreased odds of stroke recurrence after 1 year with OR 0.84 (95% CI: [0.63, 1.12]; z = −1.23, *p* = 0.22; *n* = 28,723; N = 6) [33,34,35,58,66,69] in CE/AF-related stroke patients (Supplemental Table S9; Supplemental Figure S17). Heterogeneity of studies was severe (I^2^ = 82.3%).

#### 3.3.2. Impact of Post-Stroke Statin Use in Ischemic Stroke Patients with Low Baseline LDL-Cholesterol Levels

Prevalence Estimations

The overall prevalence of all-cause mortality was 11% (95% CI: [0.07, 0.16]; z = 8.94; *p* < 0.01; *n* = 4824; N = 3) [47,51,63] (Supplemental Table S8; Supplemental Figure S12).

In statin users, the prevalence of all-cause mortality was 6% (95% CI: [0.04, 0.09]; z = 8.18; *p* < 0.01; *n* = 2799; N = 3) compared to 17% (95% CI: [0.11, 0.23]; z = 9.68; *p* < 0.01; *n* = 2025; N = 3) in statin non-users [47,51,63] (Supplemental Table S8; Supplemental Figure S13).

Outcome Associations

In patients with low baseline LDL-cholesterol, statin use was associated with significantly lower odds of all-cause mortality in IS patients with low baseline LDL-cholesterol levels with OR 0.32 (95% CI: [0.17, 0.58]; z = −3.708; *p* < 0.001; *n* = 4824; N = 3) [47,51,63] (Supplemental Table S9; Supplemental Figure S16). Heterogeneity of studies was severe (I^2^ = 85.6%).

#### 3.3.3. Impact of Post-Stroke Statin Use on CRP Levels in Ischemic Stroke Patients

Statin use was associated with significantly lower CRP levels within 3–7 days after IS (SMD = −0.41, 95% CI: [−0.75, −0.06]; z = −2.33; *p* = 0.02; *n* = 157; N = 3) [29,57,61] and after 7 days of IS (SMD = −2.64, 95% CI: [−5.26, −0.03]; z = −1.98; *p* < 0.01; *n* = 410; N = 4) [26,29,65,74] (Table 4; Supplemental Figure S18). Heterogeneity of studies was mixed, with low heterogeneity for the 3–7-day analysis (I^2^ = 0.0%) and severe for the post 7-day analysis (I^2^ = 98.6%).

### 3.4. Bias & Sensitivity Analysis

Sensitivity analysis demonstrated that the exclusion of outliers and high-risk studies did not significantly alter the pooled estimates for most analyses (Supplemental Figure S19). The *p*-values in Egger’s and Peter’s tests were insignificant for most analyses (Supplemental Tables S10 and S11; Supplemental Figure S20). Inspection of funnel plots, however, revealed asymmetry, suggesting potential for publication bias (Supplemental Figure S21).

## 4. Discussion

This meta-analysis, the ISMARDD Study, significantly contributes to the evolving understanding of statin therapy’s role in secondary prevention following IS. Beyond confirming reduced mortality and recurrence risks, ISMARDD highlights the clinical pharmacology relevance of statin therapy. The mortality benefit likely reflects pleiotropic actions, anti-inflammatory, antioxidant, and endothelial-stabilizing, beyond LDL reduction. The observed reduction in CRP provides a biomarker-based link between statin exposure and anti-inflammatory benefit, strengthening mechanistic plausibility and translational relevance.

**Figure 5:**
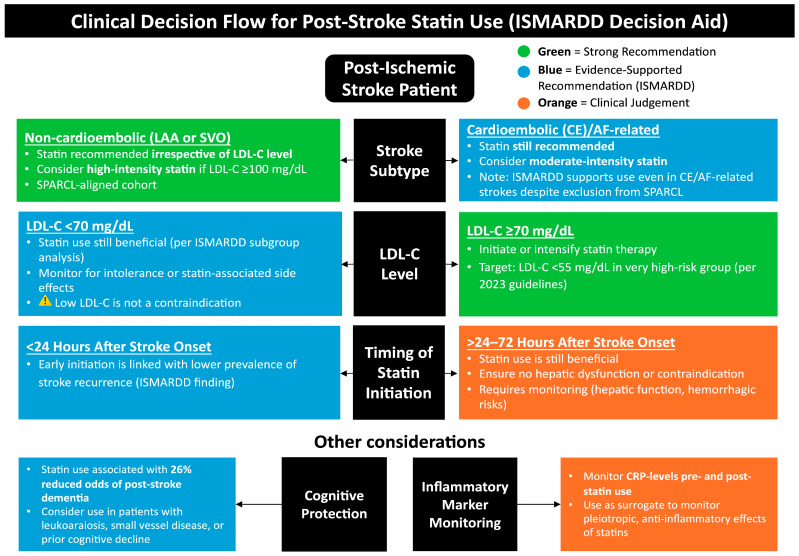
Clinical decision flow for post-stroke statin use (ISMARDD Decision Aid). This framework summarizes the recommended approach to post-stroke statin therapy based on stroke subtype, LDL-cholesterol (LDL-C) level, and timing of initiation. High-intensity statins (e.g., atorvastatin 80 mg or rosuvastatin 20 mg daily) are recommended for most patients with ischemic stroke or TIA to reduce recurrent vascular events, particularly in those with large-artery atherosclerosis (LAA) or mixed etiologies. In cardioembolic or AF-related stroke, statins may be considered to lower overall vascular risk but do not substitute for oral anticoagulation [78], which remains the cornerstone of secondary prevention. Lipid-lowering therapy should target LDL-C < 70 mg/dL or a ≥50% reduction from baseline, with lipid reassessment 4–12 weeks after initiation. Baseline liver enzyme testing is recommended, with repeat testing if clinically indicated; creatine kinase (CK) measurement is reserved for symptomatic cases. Statins are contraindicated in active liver disease or pregnancy, and dose adjustments may be required in renal impairment or when used with interacting medications [9,12,16,79,80]. Abbreviations: ISMARDD, Impact of Statin Therapy on the Risk of Stroke Recurrence, Mortality, and Dementia After Ischemic Stroke; LAA, large artery atherosclerosis; SVO, small vessel occlusion; LDL-C, low-density lipoprotein cholesterol; AF, atrial fibrillation; CE, cardioembolic; CRP, C-reactive protein.

Pooled prevalence rates were 13–23% for all-cause mortality, 4–17% for stroke recurrence, and 19% for cognitive impairment or dementia. Statin users consistently experienced lower mortality and dementia risk compared to non-users, though effects on overall cognitive impairment were neutral. No consistent trends were identified concerning statin intensity, type, or solubility. Early initiation of statins (within 24 h) was linked with a lower prevalence of stroke recurrence. While early initiation of statin therapy (<24 h) demonstrated benefit for recurrence reduction, evidence regarding statin intensity, type, and solubility remains heterogeneous and should be interpreted with caution. These pharmacologic parameters warrant validation in controlled, phenotype-stratified cohorts. Pooled association analyses showed that statin use significantly reduced the odds of all-cause mortality by 44–68%, a benefit that extended across key subgroups, including patients with CE/AF-related strokes and those with low baseline LDL-cholesterol, who showed 43–53% and 68% lower odds of mortality, respectively. The effect on stroke recurrence was modest and mixed. While the broader IS group saw a 23–24% reduction in both short- and long-term recurrence, this effect did not reach significance in the CE/AF subgroup. Despite substantial heterogeneity (I^2^ > 75%), sensitivity analyses confirmed the robustness and consistency of the mortality and recurrence findings, underscoring the generalizability of the benefit across varied study populations and methods. No significant differences were observed for PSCI and composite cognitive impairment. However, statin use was associated with a 26% lower risk of post-stroke dementia. These findings extend the evidence base beyond the SPARCL trial [6], demonstrating benefit even in real-world populations excluded from that trial, including CE/AF-related strokes and patients with low baseline LDL-cholesterol, and align with prior meta-analyses reporting consistent mortality reductions but variable effects on recurrence [17,18,76,77]. Key clinical insights relating to post-stroke statin use, generated by the ISMARDD study and by previous studies, are summarized in Figure 5, and the certainty of the findings is summarized in Table 6. Substantial heterogeneity (I^2^ > 75%) across several analyses reflects the inclusion of diverse populations, study designs, and statin regimens. Nevertheless, sensitivity analyses confirmed the consistency of direction and robustness of pooled estimates.

To clarify the clinical guidance depicted in Figure 5, in accordance with the 2021 AHA/ASA secondary prevention guideline [12], high-intensity statin therapy (e.g., atorvastatin 80 mg or rosuvastatin 20 mg daily) is preferred for most ischemic stroke or TIA patients to reduce recurrent cardiovascular and cerebrovascular events. The recommendation for statin use in CE or AF-related ischemic stroke primarily addresses comorbid atherosclerotic and systemic vascular risk rather than the embolic mechanism itself. Moderate-intensity regimens may be considered only when high-intensity therapy is contraindicated or poorly tolerated. Evidence from trials such as SPARCL supports the long-term benefit of statin therapy for secondary prevention [6], though not specifically within the first 24–48 h post-onset. All patients should have lipid profiles reassessed 4–12 weeks after initiation, with ongoing monitoring for hepatic or muscle-related adverse effects.

Mechanistic Insights and Subgroup Effects

The mortality benefit, observed in post-stroke populations, likely reflects statin’s dual mechanism of action. While fatality after IS often arises from direct neurological damage, cardiovascular complications, or systemic infections [81], statins offer pleiotropic effects—anti-inflammatory, antioxidant, and endothelial-stabilizing—that extend beyond lipid lowering [81]. These effects may explain the consistent mortality benefit observed even in subgroups such as CE/AF-related stroke and patients with low baseline LDL-cholesterol, where hyperlipidemia is not central to pathophysiology. These findings are supported by our meta-analysis of CRP, a systemic inflammation marker, which was significantly lower among statin users compared to nonusers.

Stroke Recurrence and Etiology-Specific Effects

While statin use was associated with reduced stroke recurrence in the broader IS group, this benefit did not extend to the CE/AF subgroup. This may reflect etiological differences, especially since stroke recurrence tends to follow the same mechanism as the index event [82]. Patients with large artery atherosclerosis may benefit more from statin use due to their plaque-stabilizing effects [83], whereas CE/AF-related strokes, which are typically driven by cardiac dysrhythmia, may require alternative strategies [8]. Although statins exhibit antithrombotic properties, they likely do not modify the electrophysiological mechanisms of dysrhythmias [84].

Cognitive Outcomes: Differentiating PSD and PSCI

Statin therapy was associated with a 26% reduction in PSD risk but showed no clear effect on PSCI. This divergence may reflect underlying pathophysiological differences. While PSCI is often linked to neurodegeneration [85], PSD involves vascular injury, neuroinflammation, and possible acceleration of Alzheimer’s pathology [86,87,88]. Statins’ neuroprotective and anti-inflammatory properties may better align with the pathogenesis of PSD, explaining the differential benefit [89].

Optimizing Statin Therapy in Ischemic Stroke: Toward Precision Neurology

Statins are well established in the secondary prevention of IS and transient ischemic attack (TIA). Emerging evidence, including from this meta-analysis, suggests that statins may also confer mortality and cognitive benefits. The optimal regimen, including type, dose, and timing of initiation, remains to be clearly defined to inform evidence-based clinical guidelines. Given that the majority of included studies were observational, causality cannot be inferred. Nonetheless, the consistency of effect direction across study designs supports a strong associative signal warranting further randomized validation. Although our inclusion criteria focused on post-stroke statin therapy, several studies distinguished between pre-existing and newly initiated users. Evidence suggests that continuation of pre-stroke statins may further enhance survival and reduce early neurological deterioration, consistent with the concept of ‘statin preconditioning’ [90]. Future analyses differentiating continuation versus de novo initiation are warranted.

In our analysis, early statin initiation (within 24 h of stroke onset) was linked with a lower prevalence of stroke recurrence, though this was not seen with a reduction in all-cause mortality. This finding contrasts with several trials that reported no difference in outcomes between early and delayed initiation [91,92]. Similarly, no significant association was found between statin intensity and reduced adverse outcomes, mirroring mixed results in the literature. While some studies suggest high-intensity statins yield better outcomes, others report no clear advantage [93,94]. No consistent trends emerged regarding statin type or solubility. Lipophilic statins (such as atorvastatin, simvastatin) have greater blood–brain barrier penetration, which may enhance neuroprotective effects, though their use has also been linked to potential cognitive decline [95], underscoring the need for further clarification regarding their role in stroke recovery. The ISMARDD meta-analysis also revealed variable outcome profiles among individual statins. While simvastatin demonstrated higher prevalence of mortality and recurrence, rosuvastatin exhibited the most favorable outcomes, with atorvastatin showing intermediate or inconsistent effects. These apparent differences are likely attributable to variations in study design, sample sizes, and population characteristics rather than inherent pharmacologic disparities. In contrast to cardiology trials, where lipid-lowering and plaque-stabilizing effects dominate [23,96,97,98], post-stroke outcomes may be influenced by additional factors such as blood–brain barrier penetration [99,100,101], central nervous system distribution [102], and pleiotropic anti-inflammatory mechanisms [103]. Rosuvastatin’s higher hydrophilicity and potent CRP-lowering capacity may enhance cerebrovascular protection [104], whereas variability in atorvastatin findings may reflect differential inclusion of cardioembolic and mixed-etiology cohorts. These findings underscore the need for head-to-head, stroke-specific comparative studies to delineate the neurovascular effects of individual statins beyond their cardiovascular efficacy.

Responses to statin therapy vary depending on stroke subtype and individual patient characteristics such as age, ethnicity, comorbidities, and baseline LDL-cholesterol levels. The current study showed consistent mortality benefits across subgroups, but recurrence benefits differed by stroke etiology. CE/AF-related strokes showed less consistent recurrence benefit. Genetic variability further contributes to differential statin response [105,106]; Asian patients may achieve similar outcomes with lower doses due to altered metabolism [105], while some individuals exhibit pharmacogenomic resistance to statins [79,107]. These interindividual differences underscore the importance of pharmacogenomic modifiers of statin metabolism and transport, such as SLCO1B1 and ABCG2 variants, which alter bioavailability and treatment response. Patients with variations in SLCO1B1 gene have lower hepatic uptake of statins [108], or those with ABCG2 variants have impaired statin transport and lower bioavailability [109]. In such cases, adjunctive therapies (such as ezetimibe, PCSK9 inhibitors, bempedoic acid) warrant exploration. This highlights how precision-guided statin prescribing, integrating pharmacogenomics, solubility, and inflammatory markers, can optimize secondary prevention and cognitive protection after stroke.

These interindividual variations challenge the utility of a one-size-fits-all model, which is currently reflected in guidelines. A shift toward precision neurology, tailoring therapy to stroke subtype, pharmacogenomic profile, and inflammatory or lipid biomarkers, may enhance statin efficacy and safety [106]. This is particularly relevant for low- and middle-income countries (LMICs), where access to reperfusion therapies is limited and cost-effective interventions like statins may offer critical clinical value [110].

### 4.1. Strengths and Limitations

To our knowledge, this is the first meta-analysis to comprehensively evaluate the effects of post-stroke statin therapy on key clinical outcomes across short- and long-term time points. It is also the first to meta-analyze outcomes related to PSCI and PSD, as well as in patients with low baseline LDL-cholesterol levels—populations historically underrepresented in prior trials.

Nonetheless, several limitations warrant consideration. First, variability in treatment protocols across included studies may have influenced outcomes. Although only studies evaluating post-stroke statin use were included, differences in treatment duration, adherence, and concurrent use of thrombolysis, thrombectomy, or antiplatelet agents may introduce confounding. Second, the analysis primarily relied on observational data, inherently subject to selection bias and unmeasured confounders. Third, substantial statistical heterogeneity (I^2^ > 75%) was observed in most outcomes, likely reflecting differences in patient populations and local management practices, which may limit generalizability. Fourth, limited data were available on statin parameters (type, intensity, solubility), CE/AF-related stroke outcomes, and PSCI/PSD, restricting our ability to conduct association and robust subgroup analyses. Notably, data on lacunar strokes, an important etiology particularly in Asian populations [111], were insufficient for inclusion. Fifth, analyses involving statin-specific parameters were descriptive in nature and could not adjust for confounders, precluding causal inference. Lastly, evidence of publication bias may have inflated effect estimates, impacting the reliability of conclusions.

### 4.2. Future Directions

This meta-analysis highlights key gaps that should inform future research. First, RCTs are needed to evaluate the impact of statin regimen variations—including intensity, type, and timing—on clinical outcomes to support more tailored, evidence-based recommendations. Second, longitudinal studies assessing cognitive outcomes, particularly PSCI and PSD, are essential to better understand statins’ role in long-term brain health. Future longitudinal studies should employ standardized cognitive batteries, e.g., Montreal Cognitive Assessment (MoCA), Mini Mental State Examination (MMSE), or National Institute of Neurological Disorders and Stroke and the Canadian Stroke Network (NINDS-CSN), to uniformly quantify post-stroke cognitive trajectories [112]. Third, focused research on the efficacy of statins in lacunar strokes is needed, especially given their prevalence in non-Western populations [111]. Fourth, meta-regression was limited by inadequate stratified data but will be considered in future pooled analyses. Fifth, exploring combination strategies involving statins and non-statin agents (e.g., PCSK9 inhibitors, ezetimibe) in statin-resistant individuals may help optimize outcomes in precision-guided, post-stroke lipid management. Serial measurement of CRP and related inflammatory mediators could clarify the temporal dynamics of statins’ pleiotropic effects and guide optimal initiation timing. Finally, future trials integrating genetic, lipidomic, and inflammatory biomarkers will enable precision-guided statin therapy tailored to stroke subtype and pharmacogenomic profile.

## 5. Conclusions

This comprehensive meta-analysis, the ISMARDD Study, demonstrates that post-stroke statin therapy is associated with significant reductions in all-cause mortality and PSD, with modest benefits on stroke recurrence. These effects were observed consistently across diverse IS populations, including patients with CE or AF-related strokes and those with low-baseline LDL cholesterol levels—subgroups historically excluded from key trials. Early initiation of statin therapy (within 24 h of stroke onset) was linked with reduced prevalence of recurrence, reinforcing the importance of timing in maximizing therapeutic outcomes. Although heterogeneity was present across included studies, sensitivity analyses confirmed the stability of key findings. No consistent variations were noted based on statin intensity, type, or solubility, suggesting that other biological and clinical factors may better guide individualized statin strategies. The reduction in CRP levels among statin users supports the role of anti-inflammatory and pleiotropic mechanisms in their neuroprotective effects. Taken together, these findings support a broad recommendation for statin use after IS. However, they also highlight the limitations of a one-size-fits-all approach. A shift toward precision neurology, where treatment is tailored to stroke subtype, comorbidities, LDL thresholds, cognitive risk, and inflammatory markers, may help unlock the full therapeutic potential of statins in post-stroke care [106]. Future trials should prioritize individualized treatment models and investigate optimal statin regimens for improving survival and long-term brain health in diverse stroke populations.

## Figures and Tables

**Table 1:** Clinical characteristics of studies included in meta-analysis of post-stroke statin use and clinical outcomes after ischemic stroke.

Study ID	Author	Year	Country	Study Design	Study Cohort	Control	Intervention	Cohort Size *n* (%)			Male *n* (%)			Age Mean (SD)			NIHSS Score Mean (SD)			TOAST *n* (%)										Outcomes Reported
																				LAA		CE		SVO		Other		Undetermined		
								Overall	C ^^^	I ^^^	Overall ^^^	C *	I ^+^	Overall	C	I	Overall	C	I	C *	I ^+^	C *	I ^+^	C *	I ^+^	C *	I ^+^	C *	I ^+^	
All IS Subtypes																														
1	Yakusevich et al. [70]	2012	Russia	RCT	First acute cerebrovascular incident	Standard IS therapy	Simvastatin 40 mg + Standard IS therapy	183 (100)	97 (53.0)	86 (47.0)	80 (44)	43 (44)	37 (43)	65.7 (8.3)	65.8 (9.3)	65.6 (7.2)	NR	9.2 (3.7)	8.9 (4.0)	NR	NR	NR	NR	NR	NR	NR	NR	NR	NR	Mortality, Stroke Recurrence
2	Kim et al. [49]	2024	South Korea	Retrospective	IS	No statin	Discharge statin	37,553 (100)	8764 (23.3)	28,789 (76.7)	23,237 (61.9)	NR	NR	64.9 (12.4)	NR	NR	3.0 (3.0)	NR	NR	NR	NR	NR	NR	NR	NR	NR	NR	NR	NR	PSD
3	Yang et al. [72]	2022	United Kingdom	Retrospective	IS	No statin	Statin	30,113 (100)	12,207 (40.5)	17,906 (59.5)	NR	NR	NR	NR	NR	NR	NR	NR	NR	NR	NR	NR	NR	NR	NR	NR	NR	NR	NR	PSD
4	Huang et al. [75]	2015	China	Retrospective	First-ever IS	No statin	Statin	350 (100)	214 (61.1)	136 (38.9)	244 (69.7)	NR	NR	NR	NR	NR	NR	NR	NR	NR	NR	NR	NR	NR	NR	NR	NR	NR	NR	PSCI
5	Beer et al. [29]	2012	Australia	RCT	Acute IS	Placebo	Atorvastatin 80 mg	40 (100)	23 (57.5)	17 (42.5)	NR	NR	NR	NR	NR	NR	NR	NR	NR	NR	NR	NR	NR	NR	NR	NR	NR	NR	NR	CRP Levels
6	Muscari et al. [57]	2011	Italy	RCT	IS	Placebo	Atorvastatin 80 mg	62 (100)	31 (50.0)	31 (50.0)	20 (32.2)	12 (38.7)	8 (25.8)	75.3 (11.9)	75.6 (12.2)	74.9 (11.8)	NR	13.7 (10.1)	13.7 (10.9)	6 (19.4)	6 (19.4)	10 (32.3)	10 (32.3)	6 (19.4)	5 (16.1)	1 (3.2)	0 (0)	7 (22.6)	10 (32.2)	CRP Levels
7	Zare et al. [73]	2012	Iran	RCT	Acute moderate stroke in MCA	No statin	Lovastatin 20 mg	55 (100)	30 (54.5)	25 (45.5)	29 (52.7)	16 (53.3)	13 (52.0)	NR	68.3 (10.7)	63.3 (10.8)	NR	14.5 (4.3)	16.0 (4.3)	NR	NR	NR	NR	NR	NR	NR	NR	NR	NR	Mortality
8	Aivo et al. [25]	2023	Finland	Retrospective	IS	No statin	Statin	59,588 (100)	16,171 (27.1)	43,417 (72.9)	32,604 (54.5)	8377 (51.2)	24,227 (55.8)	NR	71.7 (14.4)	70.1 (11.4)	NR	NR	NR	NR	NR	NR	NR	NR	NR	NR	NR	NR	NR	Mortality, Stroke Recurrence
9	Flach et al. [39]	2019	England	Prospective	IS	No statin	Statin	3061 (100)	2108 (68.9)	953 (31.1)	1536 (50.2)	1026 (48.7)	510 (53.5)	NR	NR	NR	NR	NR	NR	93 (4.4)	116 (12.2)	321 (15.2)	170 (17.8)	235 (11.1)	289 (30.3)	38 (1.8)	24 (2.5)	364 (17.3)	266 (27.9)	Mortality, Stroke Recurrence
10	Han et al. [43]	2021	China	Retrospective	Non-acute IS	No statin	Statin	466 (100)	279 (59.9)	187 (40.1)	NR	NR	NR	71.5 (11.2)	NR	NR	NR	NR	NR	NR	NR	NR	NR	NR	NR	NR	NR	NR	NR	Stroke Recurrence
11	Makihara et al. [53]	2013	Japan	Prospective	First ever acute IS	No statin	Statin	2822 (100)	1829 (64.8)	993 (35.2)	1677 (59.4)	1137 (62.2)	540 (54.4)	NR	71.2 (13.5)	70.0 (10.9)	NR	4.0 (3.7)	3.0 (3.0)	316 (17.3)	239 (24.1)	475 (26.0)	139 (14.0)	452 (24.7)	260 (26.2)	NR	NR	NR	NR	Mortality, Stroke Recurrence
12	Milionis et al. [55]	2009	Greece	Retrospective	First ever acute IS	No statin	Statin	794 (100)	596 (75.1)	198 (24.9)	543 (68.4)	404 (67.8)	139 (70.2)	NR	68.8 (11.1)	67.0 (10.3)	NR	NR	NR	177 (29.7)	62 (31.3)	146 (24.5)	49 (24.7)	166 (27.9)	53 (26.8)	NR	NR	107 (18.0)	34 (17.2)	Stroke Recurrence
13	O’Brien et al. [59]	2015	United States	Retrospective	IS	No statin	Statin	77,468 (100)	22,477 (29.0)	54,991 (71.0)	31,252 (40.3)	8541 (38.0)	22,711 (41.3)	NR	81.2 (8.3)	78.6 (8.2)	NR	6.0 (6.7)	5.0 (5.2)	NR	NR	NR	NR	NR	NR	NR	NR	NR	NR	Mortality, Stroke Recurrence
14	Zhang et al. [74]	2018	China	RCT	IS	Aspirin	Aspirin + Statin	127 (100)	61 (48.0)	66 (52.0)	83 (65.4)	40 (65.6)	43 (65.2)	NR	65.9 (1.8)	64.6 (1.2)	NR	NR	NR	NR	NR	NR	NR	NR	NR	NR	NR	NR	NR	Stroke Recurrence, CRP Levels
15	Jia & Zhou [45]	2013	China	Retrospective	IS with prior ICH	No statin	Atorvastatin 20 mg	354 (100)	193 (54.5)	161 (45.5)	212 (59.9)	117 (60.6)	95 (59.0)	NR	62.4 (12.8)	64.3 (12.0)	NR	NR	NR	NR	NR	NR	NR	NR	NR	NR	NR	NR	NR	Mortality, Stroke Recurrence
16	Arevalo-Lorido et al. [26]	2014	Germany	Prospective	IS	No statin	Statin	313 (100)	108 (34.5)	205 (65.5)	150 (47.9)	34 (31.5)	116 (56.6)	NR	77.5 (8.9)	NR	NR	7.9 (5.1)	NR	39 (36.1)	59 (28.8)	29 (26.9)	44 (21.5)	34 (31.5)	84 (41.0)	3 (2.8)	5 (2.4)	3 (2.8)	13 (6.3)	Mortality, CRP levels
17	Bao et al. [28]	2023	China	Retrospective	Acute IS	No statin	Low-to-moderate and high-dose statin	327 (100)	48 (14.7)	279 (85.3)	181 (55.4)	25 (52.1)	156 (55.9)	70.0 (14.2)	66.0 (17.8)	NR	9.7 (8.9)	16.7 (7.6)	NR	12 (25.0)	100 (35.8)	26 (54.2)	117 (41.9)	0 (0.0)	26 (9.3)	2 (4.2)	8 (2.9)	8 (16.7)	28 (10.0)	Mortality
18	Ni Chroinin et al. [36]	2011	Ireland	Prospective	New IS	No statin	Statin	301 (100)	112 (37.2)	189 (62.8)	153 (50.1)	52 (46.4)	101 (53.4)	NR	73.4 (15.6)	69.9 (13.1)	NR	NR	NR	1 (0.9)	23 (12.2)	51 (45.5)	56 (29.6)	12 (10.7)	33 (17.5)	4 (3.6)	4 (2.1)	44 (39.3)	72 (38.1)	Mortality
19	Montaner et al. [56]	2016	Spain	RCT	IS	Placebo	Simvastatin 40 mg	104 (100)	54 (51.9)	50 (48.1)	56 (53.8)	32 (59.3)	24 (48.0)	72.8 (14.7)	71.3 (19.0)	73.2 (13.7)	8.2 (4.1)	8.0 (5.3)	8.0 (3.8)	8 (14.8)	2 (4.0)	24 (44.4)	17 (34.0)	4 (7.4)	5 (10.0)	1 (1.9)	0 (0.0)	15 (2.8)	25 (50.0)	Mortality, Stroke Recurrence
20	Cappellari et al. [31]	2013	Italy	Retrospective	Acute IS with IVT	No statin	Statin	2072 (100)	1233 (59.5)	839 (40.5)	1210 (58.4)	705 (57.2)	505 (60.2)	66.6 (12.5)	66.1 (13.7)	67.3 (10.6)	12.6 (6.0)	13.3 (6.1)	11.7 (5.9)	NR	NR	NR	NR	NR	NR	NR	NR	NR	NR	Mortality
21	Cui et al. [37]	2022	China	Prospective	Acute IS with EVT	No statin	Low-dose statin	256 (100)	73 (28.5)	183 (71.5)	139 (54.3)	39 (53.4)	100 (54.6)	NR	67.4 (13.1)	67.1 (13.7)	NR	16.0 (6.1)	15.0 (6.0)	38 (52.1)	104 (56.8)	34 (46.6)	76 (41.5)	NR	NR	NR	NR	NR	NR	Mortality
22	Cui et al. [38]	2021	China	Prospective	Acute IS with IVT	No statin	Low-dose statin	215 (100)	35 (16.3)	180 (83.7)	102 (47.4)	17 (48.9)	85 (47.2)	NR	72.2 (14.5)	70.7 (12.1)	NR	14.0 (7.0)	8.7 (7.5)	NR	NR	NR	NR	NR	NR	NR	NR	NR	NR	Mortality
23	Scheitz et al. [62]	2015	Germany	Retrospective	Acute IS with IVT	No statin	Statin	481 (100)	398 (82.7)	83 (17.3)	241 (50.1)	198 (49.7)	43 (51.8)	NR	74.3 (12.6)	75.7 (9.1)	NR	11.7 (8.9)	10.7 (7.5)	162 (40.7)	41 (49.4)	179 (45.0)	30 (36.1)	11 (2.8)	3 (3.6)	12 (3.0)	2 (2.4)	34 (8.5)	7 (8.4)	Mortality
24	Furlan et al. [41]	2020	Brazil	Retrospective	IS	No statin	Statin	97 (100)	34 (35.1)	63 (64.9)	NR	NR	NR	NR	NR	NR	NR	NR	NR	NR	NR	NR	NR	NR	NR	NR	NR	NR	NR	Mortality
25	Winardi et al. [68]	2024	Taiwan	Retrospective	IS	No statin	Statin	78,732 (100)	39,366 (50.0)	39,366 (50.0)	42,936 (54.5)	21,468 (54.5)	21,468 (54.5)	NR	67.7 (9.1)	68.2 (9.2)	NR	NR	NR	NR	NR	NR	NR	NR	NR	NR	NR	NR	NR	Mortality
26	Song et al. [64]	2014	China	Prospective	First IS	No statin	Statin	7455 (100)	4224 (56.7)	3231 (43.3)	4584 (61.5)	2585 (61.2)	1999 (61.9)	NR	64.5 (13.2)	64.2 (12.3)	NR	5.7 (5.9)	4.7 (4.4)	NR	NR	NR	NR	NR	NR	NR	NR	NR	NR	Mortality
27	Hjalmarsson et al. [44]	2012	Sweden	Retrospective	Acute IS	No statin	Statin	744 (100)	291 (39.1)	453 (60.9)	358 (48.1)	117 (40.2)	241 (53.2)	NR	80.8 (8.0)	76.3 (7.6)	NR	NR	NR	50 (17.2)	139 (30.7)	134 (46.0)	115 (25.4)	64 (22.0)	125 (27.6)	4 (1.4)	5 (1.1)	39 (13.4)	69 (15.2)	Mortality
28	Sakurai et al. [61]	2011	Japan	Prospective	IS	No statin	Statin	146 (100)	101 (69.2)	45 (30.8)	91 (62.3)	63 (62.4)	28 (62.2)	NR	71.5 (13.6)	69.9 (12.5)	NR	7.1 (7.4)	5.0 (4.6)	22 (21.8)	17 (37.8)	51 (50.5)	14 (31.1)	21 (20.8)	13 (28.9)	NR	NR	NR	NR	CRP Levels
29	Ueno et al. [65]	2015	Japan	RCT	Acute IS	No statin	Rosuvastatin 5 mg	24 (100)	12 (50.0)	12 (50.0)	23 (95.8)	12 (100.0)	11 (91.7)	71.3 (9.0)	71.5 (10.6)	71.0 (7.6)	NR	NR	NR	NR	NR	NR	NR	NR	NR	NR	NR	NR	NR	Stroke Recurrence, CRP Levels
30	Flint et al. [40]	2011	United States	Retrospective	IS	No statin	Statin	12,689 (100)	6395 (50.4)	6294 (49.6)	5959 (47.0)	2800 (43.8)	3159 (50.2)	NR	76.5 (NR)	73.5 (NR)	NR	NR	NR	NR	NR	NR	NR	NR	NR	NR	NR	NR	NR	Mortality
31	Vitturi & Gagliardi [67]	2020	Brazil	Prospective	First ever IS	No statin	Statin	440 (100)	96 (21.8)	344 (78.2)	189 (43.0)	NR	NR	58.3 (NR)	NR	NR	NR	NR	NR	NR	NR	NR	NR	NR	NR	NR	NR	NR	NR	Mortality, Stroke Recurrence
Cardioembolic or AF-related Stroke																														
32	Choi et al. [34]	2019	South Korea	Prospective	Acute IS with AF	No statin	Low-moderate- or high-intensity statin	2153 (100)	574 (26.7)	1579 (73.3)	1120 (52.0)	322 (56.1)	798 (50.5)	73.2 (9.8)	72.7 (11.0)	NR	7.7 (8.9)	8.7 (10.4)	NR	N/A	N/A	499 (86.9)	1294 (82.0)	N/A	N/A	N/A	N/A	75 (13.1)	285 (18.0)	Mortality, Stroke Recurrence
33	Lin et al. [52]	2019	Taiwan	Retrospective	Acute IS with AF	No statin	Statin	47,882 (100)	43,242 (90.3)	4640 (9.7)	27,177 (56.8)	24,553 (56.8)	2624 (56/6)	NR	69.6 (14.2)	65.6 (12.8)	NR	NR	NR	NR	NR	NR	NR	NR	NR	NR	NR	NR	NR	Mortality
34	Ntaios et al. [58]	2014	Greece	Retrospective	Acute IS with AF	No statin	Statin	404 (100)	302 (74.8)	102 (25.2)	NR	NR	NR	67.2 (12.3)	74.6 (11.5)	73.7 (9.8)	NR	NR	NR	NR	NR	NR	NR	NR	NR	NR	NR	NR	NR	Mortality, Stroke Recurrence
35	Choi et al. [33]	2014	South Korea	Retrospective	Cardioembolic stroke	No statin	Statin	535 (100)	295 (55.1)	240 (44.9)	295 (55.1)	163 (55.3)	132 (55.0)	NR	68.6 (13.3)	NR	NR	NR	NR	N/A	N/A	295 (100)	240 (100)	N/A	N/A	N/A	N/A	N/A	N/A	Mortality, Stroke Recurrence
36	Choi et al. [35]	2024	Various	Retrospective	Acute IS with AF	No statin	Statin	20,902 (100)	13,402 (64.1)	7500 (35.9)	10,947 (52.4)	6941 (51.8)	4006 (53.4)	NR	73.5 (12.2)	73.8 (11.1)	NR	NR	NR	NR	NR	NR	NR	NR	NR	NR	NR	NR	NR	Mortality, Stroke Recurrence
37	Vitturi & Gagliardi [66]	2019	Brazil	Prospective	Cardioembolic stroke	No statin	Statin	91 (100)	18 (19.8)	73 (80.2)	47 (51.6)	7 (38.9)	40 (85.1)	NR	55.9 (13)	NR	NR	NR	NR	NR	NR	NR	NR	NR	NR	NR	NR	NR	NR	Mortality, Stroke Recurrence
38	Gong et al. [42]	2023	China	Retrospective	Cardioembolic stroke	No statin	Statin	510 (100)	106 (20.8)	404 (79.2)	227 (44.5)	47 (44.3)	180 (44.6)	70.7 (10.4)	70.7 (10.5)	70.7 (10.4)	16.3 (5.2)	17.0 (4.5)	16.3 (5.2)	N/A	N/A	106 (20.8)	404 (79.2)	N/A	N/A	N/A	N/A	N/A	N/A	Mortality
39	Park et al. [60]	2020	South Korea	Prospective	Cardioembolic stroke	No statin	Statin	2888 (100)	1025 (35.5)	1863 (64.5)	1289 (44.6)	469 (45.8)	820 (44.0)	75.3 (NR)	75.2 (1.2)	75.4 (0.9)	NR	12.3 (10.4)	8.7 (8.9)	N/A	N/A	1025	1863	N/A	N/A	N/A	N/A	N/A	N/A	Mortality, Stroke Recurrence
40	Marvardi et al. [54]	2025	Various	Prospective	Acute IS with AF	No statin	Statin	1742 (100)	844 (48.5)	898 (51.5)	811 (46.6)	357 (42.3)	454 (50.6)	NR	77.2 (10.5)	74.3 (9.1)	NR	9.2 (7.2)	7.6 (6.5)	NR	NR	NR	NR	NR	NR	NR	NR	NR	NR	Stroke Recurrence
41	Wu et al. [69]	2017	Taiwan	Retrospective	Acute IS with AF	No statin	Statin	4638 (100)	3092 (66.7)	1546 (33.3)	2277 (49.1)	1518 (49.1)	759 (49.1)	NR	75.6 (7.4)	75.6 (7.4)	NR	NR	NR	NR	NR	NR	NR	NR	NR	NR	NR	NR	NR	Stroke Recurrence
Low Baseline LDL-C Levels																														
42	Kim et al. [47]	2023	South Korea	Prospective	Acute IS with LDL-C <70 mg/dL	No statin	Statin	2850 (100)	735 (25.8)	2115 (74.2)	1810 (63.5)	448 (61.0)	1362 (64.4)	69.5 (13.4)	70.1 (13.9)	69.3 (13.2)	5.7 (6.7)	7.7 (9.7)	4.7 (5.9)	229 (31.2)	903 (42.7)	400 (54.4)	775 (36.6)	106 (14.4)	437 (20.7)	NR	NR	NR	NR	Mortality
43	Lee et al. [51]	2024	Taiwan	Retrospective	Acute IS with LDL-C <70 mg/dL	No statin	Statin	956 (100)	631 (66.0)	325 (34.0)	NR	NR	NR	76.6 (13.7)	NR	NR	NR	11.8 (9.6)	NR	NR	NR	NR	NR	NR	NR	NR	NR	NR	NR	Mortality
44	Song et al. [63]	2015	China	Prospective	Acute IS with LDL-C <1.8 mmol/L	No statin	Statin	1018 (100)	659 (64.7)	359 (35.3)	687 (67.5)	440 (66.8)	247 (68.8)	NR	65.0 (13.8)	65.6 (12.1)	NR	6.0 (6.7)	5.0 (5.2)	236 (35.8)	176 (49.0)	121 (18.4)	59 (16.4)	55 (8.3)	23 (6.4)	12 (1.8)	6 (1.7)	235 (35.7)	95 (26.5)	Mortality
Statin Intensity																														
17	Bao et al. [28]	2023	China	Retrospective	Acute IS	Low-moderate intensity statin	High-intensity statin	279 (100)	152 (54.5)	127 (45.5)	156 (55.9)	76 (50.0)	80 (63.0)	NR	71.1 (13.4)	67.6 (13.5)	NR	8.3 (8.2)	8.7 (9.0)	43 (28.3)	57 (44.9)	72 (47.4)	45 (35.4)	22 (14.5)	4 (3.1)	1 (0.7)	7 (5.5)	14 (9.2)	14 (11.0)	Mortality
45	Yang et al. [71]	2021	China	RCT	Acute IS	Rosuvastatin 5 mg	Rosuvastatin 20 mg	310 (100)	155 (50.0)	155 (50.0)	228 (73.5)	116 (74.8)	112 (72.3)	NR	66.6 (12.0)	65.0 (11.8)	NR	7.7 (6.0)	6.0 (4.5)	NR	NR	NR	NR	NR	NR	NR	NR	NR	NR	Mortality
46	Chen et al. [32]	2018	China	RCT	Anterior circulation infarct	Atorvastatin 20 mg	Atorvastatin 60 mg	117 (100)	57 (48.7)	60 (51.3)	73 (62.4)	34 (59.6)	39 (65.0)	NR	60.9 (11.9)	62.4 (11.5)	NR	NR	NR	NR	NR	NR	NR	NR	NR	NR	NR	NR	NR	Mortality, Stroke Recurrence
13	O’Brien et al. [59]	2015	United States	Retrospective	IS	Low-moderate intensity statin	High-intensity statin	29,631 (100)	20,486 (69.1)	9145 (30.9)	12,462 (42.1)	8502 (41.5)	3960 (43.3)	NR	78.9 (8.3)	77.9 (8.2)	NR	4.7 (4.4)	5.3 (5.9)	NR	NR	NR	NR	NR	NR	NR	NR	NR	NR	Mortality, Stroke Recurrence
47	Bach et al. [27]	2023	Denmark	Retrospective	First IS	Moderate intensity statin	High-intensity statin	27,387 (100)	14,355 (52.4)	13,032 (47.6)	15,495 (56.6)	7905 (55.1)	7590 (58.2)	NR	68.7 (14.1)	68.3 (13.3)	NR	NR	NR	NR	NR	NR	NR	NR	NR	NR	NR	NR	NR	Mortality, Stroke Recurrence
48	Kyto et al. [50]	2024	Finland	Retrospective	Acute IS	Low-moderate intensity statin	High-intensity statin	45,512 (100)	38,228 (84.0)	7284 (16.0)	25,293 (55.6)	20,762 (54.3)	4531 (62.2)	NR	NR	68.4 (10.7)	NR	NR	NR	NR	NR	NR	NR	NR	NR	NR	NR	NR	NR	Mortality, Stroke Recurrence
Statin Timing																														
49	Kang et al. [46]	2015	South Korea	Retrospective	Acute IS	Delayed statin (after 24 h)	Early statin (within 24 h)	167 (100)	122 (73.1)	45 (26.9)	107 (64.1)	67 (54.9)	30 (66.7)	NR	NR	67.7 (11.6)	NR	NR	13.3 (8.4)	48 (39.3)	20 (44.4)	61 (50.0)	13 (28.9)	NR	NR	NR	NR	NR	NR	Stroke Recurrence
Statin Type																														
50	Kim et al. [48]	2025	South Korea	Prospective	Acute IS	Atorvastatin	Rosuvastatin	43,512 (100)	36,903 (84.8)	6609 (15.2)	26,001 (59.8)	21,992 (59.6)	4009 (60.7)	69.2 (12.5)	69.2 (12.5)	69.7 (12.8)	3.7 (4.4)	3.7 (4.4)	3.3 (3.7)	13,976 (37.9)	2436 (36.9)	7087 (19.2)	1314 (19.9)	6609 (17.9)	1542 (23.3)	NR	NR	9231 (25.0)	1317 (19.9)	Mortality, Stroke Recurrence
51	Cao et al. [30]	2017	China	RCT	Cerebral infarction	Atorvastatin 20 mg	Rosuvastatin 10 mg	120 (100)	60 (50.0)	60 (50.0)	65 (54.2)	35 (58.3)	30 (50.0)	NR	45.8 (7.5)	46.8 (7.5)	NR	NR	NR	NR	NR	NR	NR	NR	NR	NR	NR	NR	NR	Stroke Recurrence

Abbreviations: n, number of patients; SD, standard deviation; NIHSS, National Institute of Health Stroke Scale; RCT, randomized controlled trials; IS, ischemic stroke; C, control; I, intervention; TOAST, Trial of Org 10172 in Acute Stroke Treatment; LAA, large artery atherosclerosis; CE, cardioembolic; SVO, small vessel occlusion; MCA, middle cerebral artery; ICH, intracerebral hemorrhage; IVT, intravenous thrombolysis; EVT, endovascular thrombectomy; AF, atrial fibrillation; LDL-C, low-density lipoprotein cholesterol; PSD, post-stroke dementia; PSCI, post-stroke cognitive impairment; NR, not reported; N/A; not applicable. ^^^ Percentage of total cohort size; * Percentage of control cohort size; ^+^ Percentage of intervention cohort size.

**Table 2:** Comorbidities and lifestyle factors in cohorts of ischemic stroke patients included in meta-analysis of post-stroke statin use and clinical outcomes.

Author	Year	Hypertension n (%)		Baseline Systolic Blood Pressure Mean (SD)		Diabetes Mellitus *n* (%)		Atrial Fibrillation *n* (%)		Coronary Artery Disease *n* (%)		Dyslipidaemia ^^^ *n* (%)		Previous Stroke *n* (%)		Smoking *n* (%)		Alcohol Use *n* (%)	
		C *	I ^+^	C	I	C *	I ^+^	C *	I ^+^	C *	I ^+^	C *	I ^+^	C *	I ^+^	C *	I ^+^	C *	I ^+^
All IS Subtypes																			
Yakusevich et al. [70]	2012	74 (76.3)	36 (41.9)	156.4 (15.8)	156.4 (15.3)	10 (10.3)	8 (9.3)	12 (12.4)	14 (16.3)	8 (8.2)	10 (11.6)	NR	NR	NR	NR	NR	NR	NR	NR
Kim et al. [49]	2024	NR	NR	NR	NR	NR	NR	NR	NR	NR	NR	NR	NR	NR	NR	NR	NR	NR	NR
Yang et al. [72]	2022	NR	NR	NR	NR	NR	NR	NR	NR	NR	NR	NR	NR	NR	NR	NR	NR	NR	NR
Huang et al. [75]	2015	NR	NR	NR	NR	NR	NR	NR	NR	NR	NR	NR	NR	NR	NR	NR	NR	NR	NR
Beer et al. [29]	2012	NR	NR	NR	NR	NR	NR	NR	NR	NR	NR	NR	NR	NR	NR	NR	NR	NR	NR
Muscari et al. [57]	2011	30 (96.8)	27 (87.1)	NR	NR	5 (16.1)	8 (25.8)	NR	NR	NR	NR	NR	NR	3 (9.7)	5 (16.1)	6 (19.4)	4 (12.9)	NR	NR
Zare et al. [73]	2012	20 (66.7)	13 (52.0)	NR	NR	5 (16.7)	4 (16.0)	NR	NR	NR	NR	6 (20.0)	4 (16.0)	NR	NR	8 (26.7)	9 (36.0)	NR	NR
Aivo et al. [25]	2015	95 (55.9)	102 (61.1)	NR	NR	30 (17.6)	32 (19.2)	54 (31.8)	64 (38.3)	NR	NR	NR	NR	46 (27.1)	30 (18.0)	NR	NR	NR	NR
Flach et al. [39]	2023	8894 (55.0)	26,614 (61.3)	NR	NR	3250 (20.1)	9639 (22.2)	4964 (30.7)	9421 (21.7)	1326 (8.2)	4211 (9.7)	NR	NR	NR	NR	NR	NR	NR	NR
Han et al. [43]	2019	749 (35.5)	377 (39.6)	NR	NR	1649 (78.2)	800 (83.9)	1562 (74.1)	833 (87.4)	1654 (78.5)	829 (87.0)	118 (5.6)	764 (80.2)	NR	NR	1255 (59.5)	629 (66.0)	NR	NR
Makihara et al. [53]	2021	NR	NR	NR	NR	NR	NR	NR	NR	NR	NR	NR	NR	NR	NR	NR	NR	NR	NR
Milionis et al. [55]	2013	1327 (72.3)	836 (84.2)	NR	NR	429 (23.5)	388 (39.1)	506 (27.7)	147 (14.8)	207 (11.3)	185 (18.6)	NR	NR	NR	NR	935 (51.1)	476 (47.9)	NR	NR
O’Brien et al. [59]	2009	440 (73.8)	135 (68.2)	159.0 (29.0)	158.0 (26.0)	211 (35.4)	55 (27.8)	77 (12.9)	24 (12.1)	144 (24.2)	45 (22.7)	151 (25.3)	57 (28.8)	NR	NR	233 (39.1)	80 (40.4)	NR	NR
Zhang et al. [74]	2015	17,397 (77.4)	41,518 (75.5)	NR	NR	5147 (22.9)	12,648 (23.0)	NR	NR	5237 (23.3)	11,163 (20.3)	4046 (18.0)	13,858 (25.2)	NR	NR	2113 (9.4)	7259 (13.7)	NR	NR
Jia & Zhou [45]	2018	22 (36.1)	24 (36.4)	NR	NR	25 (41.0)	28 (42.4)	NR	NR	NR	NR	NR	NR	NR	NR	NR	NR	NR	NR
Arevalo-Lorido et al. [26]	2013	137 (71.0)	109 (67.7)	NR	NR	94 (48.7)	83 (51.6)	27 (14.0)	26 (16.1)	93 (48.2)	81 (50.3)	NR	NR	NR	NR	80 (41.5)	73 (45.3)	NR	NR
Bao et al. [28]	2014	92 (85.2)	168 (82.0)	160.4 (27.4)	NR	29 (26.9)	64 (31.2)	NR	NR	NR	NR	NR	NR	NR	NR	16 (14.8)	45 (22.0)	NR	NR
Ni Chroinin et al. [36]	2023	22 (45.8)	158 (56.6)	139.0 (23.8)	NR	7 (14.6)	44 (15.8)	17 (35.4)	77 (27.6)	3 (6.3)	39 (14.0)	0 (0.0)	10 (3.6)	12 (25.0)	53 (19.0)	13 (27.1)	103 (36.9)	NR	NR
Montaner et al. [56]	2011	59 (52.7)	84 (44.4)	NR	NR	6 (5.4)	11 (5.8)	44 (39.3)	52 (27.5)	17 (15.2)	21 (11.1)	18 (16.1)	37 (19.6)	12 (10.7)	16 (8.5)	23 (20.5)	70 (37.0)	NR	NR
Cappellari et al. [31]	2016	35 (64.8)	29 (58.0)	NR	NR	7 (13.0)	10 (20.0)	3 (5.6)	12 (24.0)	1 (1.9)	2 (4.0)	8 (14.8)	7 (14.0)	3 (5.6)	4 (8.0)	13 (24.1)	10 (20.0)	NR	NR
Cui et al. [37]	2013	706 (57.3)	595 (70.9)	146.6 (20.7)	146.6 (19.6)	197 (16.0)	147 (17.5)	316 (25.6)	146 (17.4)	NR	NR	308 (25.0)	478 (57.0)	113 (9.2)	65 (7.7)	294 (23.8)	209 (24.9)	NR	NR
Cui et al. [38]	2022	34 (46.6)	92 (50.3)	135.8 (24.0)	141.4 (25.1)	12 (16.4)	32 (17.5)	NR	NR	10 (13.7)	31 (16.9)	NR	NR	NR	NR	13 (17.8)	57 (31.1)	NR	NR
Scheitz et al. [62]	2021	18 (51.4)	85 (47.2)	153.2 (32.2)	148.5 (24.7)	8 (22.9)	34 (18.9)	17 (48.6)	46 (25.6)	4 (11.4)	22 (12.2)	NR	NR	NR	NR	10 (28.6)	59 (32.8)	NR	NR
Furlan et al. [41]	2015	320 (80.4)	79 (95.2)	NR	NR	87 (21.9)	30 (36.1)	169 (42.5)	32 (38.6)	63 (15.8)	33 (39.8)	148 (37.2)	60 (72.3)	83 (20.9)	29 (34.9)	94 (23.6)	19 (22.9)	NR	NR
Winardi et al. [68]	2020	NR	NR	NR	NR	NR	NR	NR	NR	NR	NR	NR	NR	NR	NR	NR	NR	NR	NR
Song et al. [64]	2024	NR	NR	NR	NR	17,275 (43.9)	17,524 (44.5)	NR	NR	NR	NR	NR	NR	NR	NR	NR	NR	NR	NR
Hjalmarsson et al. [44]	2014	2300 (54.5)	1961 (60.7)	NR	NR	692 (16.4)	646 (20.0)	333 (7.9)	156 (4.8)	540 (12.8)	358 (11.1)	NR	NR	NR	NR	1651 (39.1)	1304 (40.4)	431 (10.2)	379 (11.7)
Sakurai et al. [61]	2012	162 (55.7)	262 (57.8)	142.9 (22.5)	141.4 (21.3)	30 (10.3)	69 (26.3)	115 (39.5)	101 (38.5)	NR	NR	10 (3.4)	108 (41.2)	123 (42.3)	90 (34.4)	NR	NR	NR	NR
Ueno et al. [65]	2011	77 (76.2)	37 (82.2)	NR	NR	22 (21.8)	12 (26.7)	43 (42.6)	8 (17.8)	19 (18.8)	10 (22.2)	7 (6.9)	45 (100.0)	NR	NR	33 (32.7)	18 (40.0)	NR	NR
Flint et al. [40]	2015	9 (75.0)	11 (91.7)	NR	NR	6 (50.0)	3 (25.0)	1 (8.3)	2 (16.7)	NR	NR	12 (100.0)	12 (100.0)	3 (25.0)	3 (25.0)	5 (41.7)	6 (50.0)	NR	NR
Vitturi & Gagliardi [67]	2011	4816 (75.3)	5363 (85.2)	NR	NR	1769 (27.7)	2449 (38.9)	1771 (27.7)	1403 (22.3)	1154 (18.0)	1914 (30.4)	NR	NR	654 (10.2)	475 (7.5)	NR	NR	NR	NR
Yakusevich et al. [70]	2020	NR	NR	NR	NR	NR	NR	NR	NR	NR	NR	NR	NR	NR	NR	NR	NR	NR	NR
Cardioembolic or AF-related stroke																			
Choi et al. [34]	2019	395 (68.8)	1109 (70.2)	NR	NR	156 (27.2)	434 (27.5)	574 (100.0)	1579 (100.0)	61 (10.6)	206 (13.0)	98 (17.1)	404 (25.6)	NR	NR	94 (16.4)	219 (13.9)	NR	NR
Lin et al. [52]	2019	31,698 (73.3)	3579 (77.1)	NR	NR	15,951 (36.9)	1857 (40.0)	NR	NR	NR	NR	NR	NR	NR	NR	NR	NR	NR	NR
Ntaios et al. [58]	2014	224 (74.2)	83 (81.4)	NR	NR	63 (20.9)	20 (19.6)	262 (86.8)	86 (84.3)	NR	NR	83 (27.5)	53 (52.0)	NR	NR	52 (17.2)	21 (20.6)	NR	NR
Choi et al. [33]	2014	199 (67.5)	171 (71.3)	NR	NR	56 (19.0)	67 (27.9)	NR	NR	30 (10.2)	43 (17.9)	NR	NR	NR	NR	76 (25.8)	67 (27.9)	NR	NR
Choi et al. [35]	2024	9424 (70.3)	3693 (49.2)	NR	NR	1305 (9.7)	1597 (21.3)	11,298 (84.3)	4219 (56.3)	NR	NR	5468 (40.8)	2420 (32.3)	NR	NR	NR	NR	NR	NR
Vitturi & Gagliardi [66]	2019	12 (66.7)	58 (79.5)	NR	NR	4 (22.2)	21 (28.8)	9 (50.0)	40 (54.8)	2 (11.1)	18 (24.7)	2 (11.1)	14 (19.2)	4 (22.2)	16 (21.9)	6 (33.3)	18 (24.7)	NR	NR
Gong et al. [42]	2023	52 (49.1)	207 (51.2)	144.0 (25.4)	144.0 (24.4)	18 (17.0)	65 (16.1)	77 (72.6)	276 (68.3)	14 (13.2)	84 (20.8)	12 (11.3)	43 (10.6)	23 (21.7)	73 (18.1)	13 (12.3)	50 (12.4)	NR	NR
Park et al. [60]	2020	708 (69.1)	1297 (69.6)	142.0 (2.6)	140.5 (1.7)	267 (26.0)	460 (24.7)	940 (91.7)	1615 (86.7)	NR	NR	153 (14.9)	569 (30.5)	NR	NR	114 (11.1)	215 (11.5)	NR	NR
Marvardi et al. [54]	2025	623 (73.8)	720 (80.2)	NR	NR	165 (19.5)	231 (25.7)	312 (37.0)	420 (46.8)	92 (10.9)	163 (18.2)	130 (15.4)	445 (49.6)	199 (23.6)	257 (28.6)	NR	NR	51 (6.0)	70 (7.8)
Wu et al. [69]	2017	2986 (96.6)	1493 (96.6)	NR	NR	1036 (33.5)	518 (33.5)	NR	NR	2028 (65.6)	1014 (65.6)	NR	NR	NR	NR	NR	NR	NR	NR
Low Baseline LDL-C Levels																			
Kim et al. [47]	2023	437 (59.5)	1365 (64.5)	NR	NR	240 (32.7)	807 (38.2)	325 (44.2)	627 (29.6)	NR	NR	37 (5.0)	239 (11.3)	NR	NR	173 (23.5)	604 (28.6)	NR	NR
Lee et al. [51]	2024	476 (75.4)	NR	NR	NR	254 (40.3)	NR	192 (30.4)	NR	132 (20.9)	NR	NR	NR	202 (32.0)	NR	143 (22.7)	NR	NR	NR
Song et al. [63]	2015	373 (56.6)	230 (70.8)	NR	NR	121 (18.4)	71 (21.8)	95 (14.4)	38 (11.7)	87 (13.2)	47 (14.5)	NR	NR	215 (32.6)	115 (35.4)	294 (44.6)	151 (46.5)	71 (10.8)	34 (10.5)
Statin Intensity																			
Bao et al. [28]	2023	93 (61.2)	65 (51.2)	146.5 (24.2)	151.1 (26.1)	28 (18.4)	16 (12.6)	53 (34.9)	24 (18.9)	24 (15.8)	15 (11.8)	5 (3.3)	5 (3.9)	36 (23.7)	17 (13.4)	55 (36.2)	48 (37.8)	NR	NR
Yang et al. [71]	2021	85 (54.8)	85 (54.8)	NR	NR	21 (13.5)	19 (12.3)	25 (16.1)	9 (5.8)	21 (13.5)	11 (7.1)	9 (5.8)	7 (4.5)	28 (18.1)	25 (16.1)	NR	NR	NR	NR
Chen et al. [32]	2018	41 (71.9)	45 (75.0)	144.0 (22.6)	143.5 (22.1)	17 (29.8)	21 (35.0)	NR	NR	3 (5.3)	5 (8.3)	18 (31.6)	23 (38.3)	5 (8.8)	7 (11.7)	25 (43.9)	28 (46.7)	25 (43.9)	22 (36.7)
O’Brien et al. [59]	2015	15,201 (74.2)	6850 (74.9)	NR	NR	4650 (22.7)	2268 (24.8)	NR	NR	4261 (20.8)	2094 (22.9)	5183 (25.3)	2405 (26.3)	NR	NR	2684 (13.1)	1244 (13.6)	NR	NR
Bach et al. [27]	2023	8100 (56.4)	7387 (56.7)	NR	NR	1147 (8.0)	1078 (8.3)	NR	NR	NR	NR	NR	NR	NR	NR	4688 (32.7)	3862 (29.6)	13,528 (94.2)	11,667 (89.5)
Kyto et al. [50]	2024	23,107 (50.8)	4786 (10.5)	NR	NR	8625 (19.0)	1981 (4.4)	8410 (18.5)	1508 (3.3)	NR	NR	NR	NR	NR	NR	NR	NR	NR	NR
Statin Timing																			
Kang et al. [46]	2015	74 (60.7)	28 (62.2)	NR	NR	22 (18.0)	10 (22.2)	49 (40.2)	15 (33.3)	NR	NR	NR	NR	18 (14.8)	12 (26.7)	NR	NR	NR	NR
Statin Type																			
Kim et al. [48]	2025	24,562 (66.6)	4550 (68.8)	146.1 (27.7)	152.4 (27.7)	12,426 (33.7)	2372 (35.9)	7214 (19.5)	1275 (19.3)	3146 (8.5)	625 (9.5)	12,656 (34.3)	2519 (38.1)	7274 (19.7)	1543 (23.3)	9010 (24.4)	1490 (22.5)	NR	NR
Cao et al. [30]	2017	28 (46.7)	25 (41.7)	NR	NR	10 (16.7)	11 (18.3)	NR	NR	NR	NR	15 (25.0)	16 (26.7)	NR	NR	7 (11.7)	8 (13.3)	NR	NR

Abbreviations: *n*, number of patients; SD, standard deviation; C, control; I, intervention; NR, not reported; AF, atrial fibrillation; LDL-C, low-density lipoprotein-cholesterol; ^^^ Includes any reported hyperlipidemia and hypercholesterolemia. * Percentage of control cohort size; ^+^ Percentage of intervention cohort size.

**Table 3:** Pooled prevalence of clinical outcomes in all ischemic stroke subtypes.

Outcome	Time Frame	Analysis Subgroup	Overall							Statin Nonusers							Statin Users						
			Summary Effects		Heterogeneity				Heterogeneity Variance Estimates	Summary Effects		Heterogeneity				Heterogeneity Variance Estimates	Summary Effects		Heterogeneity		Heterogeneity Variance Estimates		
			Pooled Prevalence (95% CI)	Test of ES = 0	Chi-Squared	H	I^2^ (%)	*p*-Value	τ^2^ ≤ Φ	Pooled Prevalence (95% CI)	Test of ES = 0	Chi-Squared	H	I^2^ (%)	*p*-Value	τ^2^ ≤ Φ	Pooled Prevalence (95% CI)	Test of ES = 0	Chi-squared	H	I^2^ (%)	*p*-Value	τ^2^ ≤ Φ
Dementia or Cognitive Impairment		Dementia	-	-	-	-	-	-	-	0.12 [0.11, 0.12]	z = 98.99, *p* < 0.01	-	-	-	-	-	0.11 [0.11, 0.11]	z = 144.13, *p* < 0.01	-	-	-	-	-
		Cognitive Impairment	-	-	-	-	-	-	-	0.36 [0.29, 0.42]	z = 17.72, *p* < 0.01	-	-	-	-	-	0.46 [0.37, 0.54]	z = 16.33, *p* < 0.01	-	-	-	-	-
		Overall	0.18 [0.09, 0.30]	z = 6.16, *p* < 0.01	1939.56	-	99.90	<0.01	0.06	0.19 [0.09, 0.31]	z = 6.05, *p* < 0.01	619.12	-	99.68	<0.01	0.06	0.19 [0.09, 0.31]	z = 5.67, *p* < 0.01	1444.50	-	99.86	<0.01	0.06
All-Cause Mortality	≤3 months	Retrospective	0.18 [0.12, 0.24]	z = 9.75, *p* < 0.01	-	36.14	91.70	<0.01	-	0.25 [0.16, 0.34]	z = 8.93, *p* < 0.01	-	27.15	88.95	<0.01	-	0.11 [0.06, 0.19]	z = 5.77, *p* < 0.01	-	26.21	88.55	<0.01	-
		Prospective	0.13 [0.07, 0.21]	z = 6.34, *p* < 0.01	-	-	-	-	-	0.25 [0.08, 0.47]	z = 4.13, *p* < 0.01	-	-	-	-	-	0.07 [0.03, 0.13]	z = 5.14, *p* < 0.01	-	-	-	-	-
		RCT	0.04 [0.02, 0.07]	z = 6.17, *p* < 0.01	-	-	-	-	-	0.04 [0.01, 0.09]	z = 3.28, *p* < 0.01	-	-	-	-	-	0.03 [0.01, 0.07]	z = 3.50, *p* < 0.01	-	-	-	-	-
		Overall	0.12 [0.09, 0.16]	z = 11.67, *p* < 0.01	-	160.04	94.38	<0.01	0.03	0.18 [0.13, 0.24]	z = 10.38, *p* < 0.01	-	140.81	93.61	<0.01	0.04	0.08 [0.05, 0.11]	z = 8.38, *p* < 0.01	-	77.56	88.40	<0.01	0.02
	≤1 year	Retrospective	0.17 [0.08, 0.30]	z = 5.31, *p* < 0.01	-	4303.98	99.86	<0.01	-	0.26 [0.13, 0.42]	z = 5.86, *p* < 0.01	-	2321.27	99.74	<0.01	-	0.11 [0.05, 0.19]	z = 5.01, *p* < 0.01	-	1364.31	99.56	<0.01	-
		Prospective	0.14 [0.07, 0.24]	z = 5.86, *p* < 0.01	-	113.27	97.35	<0.01	-	0.26 [0.10, 0.45]	z = 4.76, *p* < 0.01	-	97.91	96.94	<0.01	-	0.08 [0.03, 0.16]	z = 4.32, *p* < 0.01	-	73.56	95.92	<0.01	-
		RCT	0.07 [0.01, 0.17]	z = 2.82, *p* < 0.01	-	-	-	-	-	0.06 [0.00, 0.18]	z = 2.08, *p* < 0.01	-	-	-	-	-	0.08 [0.02, 0.17]	z = 3.37, *p* < 0.01	-	-	-	-	-
		Overall	0.14 [0.08, 0.21]	z = 7.82 *p* < 0.01	-	4436.65	99.71	<0.01	0.11	0.21 [0.14, 0.30]	z = 8.49, *p* < 0.01	-	2445.12	99.47	<0.01	0.13	0.09 [0.06, 0.14]	z = 7.30, *p* < 0.01	-	1453.11	99.11	<0.01	0.07
	>1 year	Retrospective	0.35 [0.07, 0.71]	z = 3.28, *p* < 0.01	-	-	-	-	-	0.38 [0.12, 0.69]	z = 4.01, *p* < 0.01	-	-	-	-	-	0.34 [0.05, 0.72]	z = 3.04, *p* < 0.01	-	-	-	-	-
		Prospective	0.17 [0.06, 0.31]	z = 4.79, *p* < 0.01	-	576.47	99.31	<0.01	-	0.26 [0.13, 0.42]	z = 5.73, *p* < 0.01	-	305.38	98.69	<0.01	-	0.12 [0.04, 0.24]	z = 4.26, *p* < 0.01	-	223.24	98.21	<0.01	-
		Overall	0.23 [0.07, 0.44]	z = 4.20, *p* = 0.01	-	36,960.44	99.98	<0.01	0.42	0.30 [0.14, 0.50]	z = 5.30, *p* < 0.01	-	11,553.88	99.94	<0.01	0.33	0.19 [0.05, 0.41]	z = 3.58, *p* < 0.01	-	23,089.78	99.97	<0.01	0.46
Stroke Recurrence	≤1 year	Retrospective	0.03 [0.00, 0.09]	z = 2.53, *p* = 0.01	-	14.26	78.97	<0.01	-	0.08 [0.08, 0.09]	z = 73.79, *p* < 0.01	-	-	-	-	-	0.07 [0.06, 0.07]	z = 107.27, *p* < 0.01	-	-	-	-	-
		RCT	0.07 [0.07, 0.07]	z = 130.56, *p* < 0.01	-	-	-	-	-	0.04 [0.00, 0.11]	z = 2.43, *p* = 0.02	-	8.57	65.01	0.04	-	0.02 [0.00, 0.06]	z = 1.84, *p* = 0.07	-	5.90	49.13	0.12	-
		Overall	0.04 [0.02, 0.08]	z = 4.38, *p* < 0.01	-	21.48	81.38	<0.01	0.02	0.05 [0.02, 0.10]	z = 4.59, *p* < 0.01	-	11.34	64.74	0.02	0.02	0.03 [0.01, 0.07]	z = 3.41, *p* < 0.01	-	10.91	63.32	0.03	0.02
	>1 year	Retrospective	0.20 [0.12, 0.30]	z = 7.62, *p* < 0.01	-	4717.49	99.92	<0.01	-	0.23 [0.13, 0.34]	z = 7.58, *p* < 0.01	-	1566.58	99.74	<0.01	-	0.15 [0.08, 0.25]	z = 6.47, *p* < 0.01	-	3191.48	99.87	<0.01	-
		Prospective	0.12 [0.07, 0.18]	z = 7.99, *p* < 0.01	-	-	-	-	-	0.14 [0.08, 0.22]	z = 7.28, *p* < 0.01	-	-	-	-	-	0.10 [0.05, 0.16]	z = 6.92, *p* < 0.01	-	-	-	-	-
		Overall	0.17 [0.11, 0.24]	z = 9.02, *p* < 0.01	-	5040.67	99.86	<0.01	0.06	0.20 [0.13, 0.28]	z = 9.31, *p* < 0.01	-	1761.82	99.60	<0.01	0.07	0.13 [0.08, 0.20]	z = 7.69, *p* < 0.01	-	3338.94	99.79	<0.01	0.06

Abbreviations: CI, confidence interval; ES, effect size; H, heterogeneity statistic; RCT, randomized controlled trial. Note: Φ represents the heterogeneity variance threshold used to assess model convergence. τ^2^ ≤ Φ denotes that the estimated between-study variance lies within the acceptable tolerance range for random-effects meta-analysis.

**Table 4:** Summary effects and heterogeneity obtained from meta-analysis of statin use and clinical outcomes after ischemic stroke.

Outcome	Time Frame	N	*n*	Effect Measure	Analysis Subgroup	Summary Effects			Heterogeneity				Heterogeneity Variance Estimates
						OR (95% CI)	SMD (95% CI)	Test of Overall Effect	Cochran’s Q	H	I^2^ (%)	*p*-Value	τ^2^ ≤ Φ
Post-Stroke Dementia or Cognitive Decline	-	2	67,666	OR	Post-Stroke Dementia	0.74 [0.60, 0.90]	-	z = −2.94, *p* < 0.01	12.69	-	92.1	<0.01	0.02
		1	350		Post-Stroke Cognitive Impairment	1.52 [0.98, 2.36]		z = 1.886, *p* = 0.06	0.00		-	-	-
		3	68,016		Overall	0.83 [0.66, 1.05]		z = −1.54, *p* = 0.12	21.95	3.31	90.9	<0.01	0.03
All-Cause Mortality	≤3 months	4	2749	OR	Retrospective	0.36 [0.27, 0.47]	-	z = −7.41, *p* < 0.01	2.36	-	0.0	0.50	-
		3	8142		Prospective	0.26 [0.18, 0.39]		z = −7.09, *p* < 0.01	4.10		51.2	0.13	0.06
		3	342		RCT	0.73 [0.25, 2.11]		z = −0.58, *p* = 0.57	1.13		0.0	0.57	-
		10	11,233		Overall	0.32 [0.26, 0.39]		z = −12.438, *p* < 0.01	11.16	1.11	19.4	0.27	0.02
	≤1 year	7	75,770	OR	Retrospective	0.37 [0.28, 0.47]	-	z = −7.554, *p* < 0.01	63.30	-	90.5	<0.01	0.08
		4	8455		Prospective	0.31 [0.26, 0.36]		z = −13.26, *p* < 0.01	2.62		0.0	0.45	-
		3	342		RCT	0.93 [0.46, 1.90]		z = −0.20, *p* = 0.84	0.80		0.0	0.81	-
		14	84,567		Overall	0.35 [0.28, 0.44]		z = −9.30, *p* < 0.01	96.31	2.75	86.8	<0.01	0.09
	>1 year	3	156,554	OR	Retrospective	0.78 [0.54, 1.12]	-	z = −1.36, *p* = 0.17	292.93	-	99.3	<0.01	0.08
		5	6794		Prospective	0.41 [0.27, 0.62]		z = −4.20, *p* < 0.01	18.56		78.4	0.01	0.14
		8	163,348		Overall	0.56 [0.44, 0.72]		z = −4.51, *p* < 0.01	326.91	6.83	97.9	<0.01	0.08
Stroke Recurrence	≤1 year	1	59,588	OR	Retrospective	0.77 [0.72, 0.82]	-	z = −7.59, *p* < 0.01	0.00	-	-	-	-
		4 *	438		RCT	0.62 [0.24, 1.60]		z = −0.99, *p* = 0.32	1.68		0.0	0.64	-
		5 *	60,026		Overall	0.77 [0.72, 0.82]		z = −7.64, *p* < 0.01	1.88	0.69	0.0	0.76	<0.01
	>1 year	5	138,670	OR	Retrospective	0.82 [0.70, 0.95]	-	z = −2.60, *p* = 0.01	32.50	-	87.1	<0.001	0.02
		3	6323		Prospective	0.68 [0.51, 0.89]		z = −2.79, *p* = 0.01	3.81		47.5	0.15	0.03
		8	144,993		Overall	0.76 [0.66, 0.87]		z = −3.88, *p* < 0.01	46.86	2.59	85.1	<0.01	0.02
CRP Levels	3–7 days	3	248	SMD	-	-	−0.41 [−0.75, −0.06]	z = −2.33, *p* = 0.02	0.01	0.08	0.0	0.993	<0.01
	>7 days	4	504	SMD	-	-	−2.64 [−5.26, −0.03]	z = −1.98, *p* < 0.01	207.97	8.33	98.6	< 0.01	6.96

Abbreviations: N, number of studies; *n*, number of patients; RCT, randomized controlled trial; OR, odds ratio; SMD, standardized mean difference; CI, confidence interval; CRP, C-reactive protein; * Due to model limitations, studies with 0 events were replaced with 0.5 to allow for OR calculation. Note: Φ represents the heterogeneity variance threshold used to assess model convergence. τ^2^ ≤ Φ denotes that the estimated between-study variance lies within the acceptable tolerance range for random-effects meta-analysis.

**Table 5:** Pooled prevalence and summary effects of clinical outcomes after ischemic stroke by statin parameters.

Parameter	Outcome	Timeframe	N	*n*	Effect Measure	Variation/ Comparison	Effect Size			
							Pooled Prevalence (95% CI)	Test of ES = 0	OR (95% CI)	Test of Overall Effect
Statin Type	All-Cause Mortality	≤1 year	2	136	Prevalence	Simvastatin	0.10 [0.06, 0.16]	z = 6.44, *p* < 0.01	N/A	N/A
			1	25		Lovastatin	0.04 [0.00, 0.20]	z = 1.42, *p* = 0.16	N/A	N/A
			3	37,020		Atorvastatin	0.05 [0.02, 0.08]	z = 5.18, *p* < 0.01	N/A	N/A
			3	6919		Rosuvastatin	0.04 [0.01, 0.07]	z = 4.55, *p* < 0.01	N/A	N/A
	Stroke Recurrence	≤1 year	2	136	Prevalence	Simvastatin	0.03 [0.00, 0.06]	z = 2.89, *p* < 0.01	N/A	N/A
			4	37,080		Atorvastatin	0.04 [0.03, 0.05]	z = 10.93, *p* < 0.01	N/A	N/A
			3	6681		Rosuvastatin	0.02 [0.00, 0.07]	z = 1.47, *p* = 0.14	N/A	N/A
Statin Solubility	All-Cause Mortality	≤1 year	2	6919	Prevalence	Hydrophilic	0.06 [0.05, 0.06]	z = 36.84, *p* < 0.01	N/A	N/A
			5	37,181		Lipophilic	0.06 [0.03, 0.10]	z = 5.88, *p* < 0.01	N/A	N/A
	Stroke Recurrence	≤1 year	3	6681	Prevalence	Hydrophilic	0.02 [0.00, 0.07]	z = 1.47, *p* = 0.14	N/A	N/A
			5	37,216		Lipophilic	0.04 [0.02, 0.06]	z = 6.37, *p* < 0.01	N/A	N/A
Statin Timing	All-Cause Mortality	90-day	3	316	Prevalence	≤1 day	0.10 [0.06, 0.12]	z = 7.13, *p* < 0.01	N/A	N/A
			3	1169		>1 day	0.06 [0.03, 0.09]	z = 7.45, *p* < 0.01	N/A	N/A
	Stroke Recurrence	90-day	2	95	Prevalence	≤1 day	0.05 [0.01, 0.11]	z = 3.44, *p* < 0.01	N/A	N/A
			2	239		>1 day	0.09 [0.06, 0.14]	z = 8.45, *p* < 0.01	N/A	N/A
Statin Intensity	All-Cause Mortality	≤2 years	1	25	Prevalence	Low	0.04 [0.00, 0.20]	z = 1.42, *p* = 0.16	N/A	N/A
			4	21,001		Low-moderate	0.19 [0.12, 0.28]	z = 8.31, *p* < 0.01	N/A	N/A
			4	348		Moderate	0.05 [0.01, 0.13]	z = 2.68, *p* = 0.01	N/A	N/A
			2	471		Moderate-high	0.04 [0.02, 0.06]	z = 7.54, *p* < 0.01	N/A	N/A
			3	9360		High	0.09 [0.00, 0.32]	z = 1.48, *p* = 0.14	N/A	N/A
		N/A	6	103,028	OR	Lower intensity vs. higher intensity	N/A	N/A	0.92 [0.76, 1.12]	z = −0.80, *p* = 0.42
	Stroke Recurrence	≤2 years	1	20,486	Prevalence	Low-moderate	0.10 [0.10, 0.11]	z = 93.47, *p* = 0.01	N/A	N/A
			4	205		Moderate	0.04 [0.00, 0.10]	z = 2.40, *p* = 0.02	N/A	N/A
			1	344		Moderate-high	0.17 [0.13, 0.22]	z = 14.90, *p* < 0.01	N/A	N/A
			2	9205		High	0.10 [0.10, 0.11]	z = 55.10, *p* = 0.12	N/A	N/A
		N/A	4	102,647	OR	Lower intensity vs. higher intensity	N/A	N/A	1.10 [0.88, 1.38]	z = −0.82, *p* = 0.41

Abbreviations: N, number of studies; *n*, number of patients; CI, confidence interval; ES, effect size; OR, odds ratio; N/A, not available.

**Table 6:** Grading of Recommendations Assessment, Development and Evaluation (GRADE) Summary of Findings: Statin Therapy After Ischemic Stroke.

Outcome	No. of Studies (Participants)	Study Design	Relative Effect (95% CI)	Assumed Risk (Control)	Risk with Statin	Absolute Effect	Certainty of Evidence	Reasons
All-cause mortality, ≤1 year	14 (*n* = 84,567)	Observational and RCTs (adjusted estimates pooled via random-effects)	OR 0.35 (0.28–0.44)	210 per 1000	90 per 1000	120 fewer per 1000	⊕⊕◯◯ Low to Moderate	−1 risk of bias; −1 inconsistency; +1 large effect
Stroke recurrence, ≤1 year	5 (*n* = 60,026)	Observational and RCTs (random-effects)	OR 0.77 (0.72–0.82)	—	—	—	⊕⊕◯◯ Low	−1 risk of bias; −1 inconsistency; −1 zero-event handling
Post-stroke dementia (any time)	2 (subset)	Observational (random-effects)	OR 0.74 (0.60–0.90)	—	—	—	⊕⊕◯◯ Low	−1 risk of bias; −1 imprecision; −1 inconsistency likely
CRP (3–7 d/>7 d)	3/4; *n* = 248/504	Observational and RCTs; continuous outcome (SMD)	SMD −0.41 (−0.75 to −0.06) at 3–7 d; SMD −2.64 (−5.26 to −0.03) at >7 d	—	—	—	⊕◯◯◯ Very low	−1 risk of bias; −1 inconsistency; −1 indirectness

GRADE Working Group grades of evidence: ⊕⊕⊕⊕ High: We are very confident that the true effect lies close to that of the estimate of the effect. ⊕⊕⊕◯ Moderate: We are moderately confident in the effect estimate; the true effect is likely to be close to the estimate of the effect, but there is a possibility that it is substantially different. ⊕⊕◯◯ Low: Our confidence in the effect estimate is limited; the true effect may be substantially different from the estimate of the effect. ⊕◯◯◯ Very low: We have very little confidence in the effect estimate; the true effect is likely to be substantially different from the estimate; The Assumed Risk comes from control group event rates in the included studies. The Relative Effect (OR) is applied to this baseline to estimate the risk under statin therapy. The Absolute Effect is simply the difference between assumed control risk and the estimated statin risk, expressed per 1000 patients for clarity. Abbreviations: n, number of participants; RCTs, randomized controlled trial; SMD, standardized mean difference; OR, odds ratio.

## Data Availability

The original contributions presented in this study are included in the article. Further inquiries can be directed to the corresponding author.

## Supplementary Materials

The following supporting information can be downloaded at: https://www.mdpi.com/article/10.3390/neurolint17110176/s1, Supplemental Tables: Table S1. Search Strategy (Keywords/MeSH Terms); Table S2. Preferred Reporting Items for Systematic Reviews and Meta-analyses (PRISMA) (2020) Checklist; Table S3. Meta-analysis of Observational Studies in Epidemiology (MOOSE) Checklist; Table S4. Modified Jadad Analysis for Methodological Quality; Table S5. ROBINS-I: Risk of Bias in Non-Randomized Studies of Interventions; Table S6. RoB-2: Risk of Bias in Randomized Trials; Table S7. Funding Bias Scores for Studies; Table S8. Pooled prevalence of clinical outcomes in ischemic stroke patient subgroups with post-stroke statin use vs. no post-stroke statin use; Table S9. Summary effects and heterogeneity obtained from meta-analysis of statin use and clinical outcomes after ischemic stroke in sub-groups; Table S10. Outputs of Peters’ test; Table S11. Outputs of Egger’s test; Supplemental Figures: Figure S1. Estimated prevalence of all-cause mortality within 3 months, 1 year and after 1 year of ischemic stroke; Figure S2. Estimated prevalence of all-cause mortality within 3 months, 1 year and after 1 year of ischemic stroke in statin users vs. nonusers, as shown in Figure S3. Estimated prevalence of stroke recurrence within 1 year and after 1 year of ischemic stroke; Figure S4. Estimated prevalence of stroke recurrence within 1 year and after 1 year of ischemic stroke in statin users vs. nonusers; Figure S5. Estimated prevalence of dementia/cognitive impairment after ischemic stroke; Figure S6. Estimated prevalence of dementia/cognitive impairment after ischemic stroke in statin users vs. nonusers; Figure S7. Estimated prevalence of all-cause mortality and stroke recurrence by statin timing of initiation; Figure S8. Estimated prevalence of all-cause mortality and stroke recurrence by statin type; Figure S9. Estimated prevalence of all-cause mortality and stroke recurrence by statin solubility; Figure S10. Estimated prevalence of all-cause mortality and stroke recurrence by statin intensity; Figure S11. Association between increasing statin intensity and all-cause mortality and stroke recurrence after ischemic stroke; Figure S12. Estimated prevalence of all-cause mortality within and after 1 year in cardioembolic/atrial fibrillation stroke patients and patients with low baseline low-density lipoprotein cholesterol; Figure S13. Estimated prevalence of all-cause mortality within and after 1 year in cardioembolic/atrial fibrillation stroke patients and patients with low baseline low-density lipoprotein cholesterol in statin users vs. nonusers; Figure S14. Estimated prevalence of stroke recurrence within and after 1 year in cardioembolic/atrial fibrillation stroke patients; Figure S15. Estimated prevalence of stroke recurrence within and after 1 year in cardioembolic/atrial fibrillation stroke patients in statin users vs. nonusers; Figure S16. Association between statin use and all-cause mortality within and after 1 year in cardioembolic and atrial-fibrillation related stroke patients and patients with low baseline low-density lipoprotein cholesterol; Figure S17. Association between statin use and stroke recurrence after 1 year in cardioembolic and atrial-fibrillation related stroke patients; Figure S18. Difference in CRP levels (mg/L) within 3–7 days and after 7 days of ischemic stroke between statin users and nonusers; Figure S19. Graphs of Sensitivity Analysis; Figure S20. Graphs of Egger’s Regression Test; Figure S21. Graphs of Funnel Plots.
