# Supplementary material for: Impact of Statin Therapy on the Risk of Stroke Recurrence, Mortality, and Dementia After Ischemic Stroke (ISMARDD Study): A Comprehensive Meta-Analysis
Source: Neurol Int. 2025 Nov 1;17(11):176. doi: 10.3390/neurolint17110176 (PMC12655416; doi:10.3390/neurolint17110176)
Supplement: Supplementary file 1 [file neurolint-17-00176-s001.zip › neurolint-3870307-supplementary.pdf]

SUPPLEMENTARY INFORMATION

Impact of Statin Therapy on the Risk of Stroke Recurrence, Mortality, and Dementia After Acute Ischemic Stroke (ISMARDD Study): A Comprehensive Meta-Analysis

Authors: Muskaan Gupta, Kevin J. Spring, Roy G. Beran, and Sonu Bhaskar*

***Corresponding author:** Prof. Sonu Bhaskar, MD, PhD, FANA
Email: Sonu.Bhaskar@globalhealthneurolab.org

Current address:
National Cerebral and Cardiovascular Center (NCVC)
Division of Cerebrovascular Medicine & Neurology
Department of Neurology
6-1 Kishibeshimmachi
Suita, Osaka
564-8565, Japan

List of Supplemental Information

1. **Supplemental Tables**
    a. Table S1. Search Strategy (Keywords/MeSH Terms)
    b. Table S2. Preferred Reporting Items for Systematic Reviews and Meta-analyses (PRISMA) (2020) Checklist
    c. Table S3. Meta-analysis of Observational Studies in Epidemiology (MOOSE) Checklist
    d. Table S4. Modified Jadad Analysis for Methodological Quality
    e. Table S5. ROBINS-I: Risk of Bias in Non-Randomized Studies of Interventions
    f. Table S6. RoB-2: Risk of Bias in Randomized Trials
    g. Table S7. Funding Bias Scores for Studies
    h. Table S8. Pooled prevalence of clinical outcomes in ischemic stroke patient subgroups with post-stroke statin use vs no post-stroke statin use
    i. Table S9. Summary effects and heterogeneity obtained from meta-analysis of statin use and clinical outcomes after ischemic stroke in sub-groups
    j. Table S10. Outputs of Peters' test
    k. Table S11. Outputs of Egger's test

2. **Supplemental Figures**
    a. Figure S1. Estimated prevalence of all-cause mortality within 3 months, 1 year and after 1 year of ischemic stroke.
    b. Figure S2. Estimated prevalence of all-cause mortality within 3 months, 1 year and after 1 year of ischemic stroke in statin users vs nonusers.
    c. Figure S3. Estimated prevalence of stroke recurrence within 1 year and after 1 year of ischemic stroke.
    d. Figure S4. Estimated prevalence of stroke recurrence within 1 year and after 1 year of ischemic stroke in statin users vs nonusers.
    e. Figure S5. Estimated prevalence of dementia/cognitive impairment after ischemic stroke.
    f. Figure S6. Estimated prevalence of dementia/cognitive impairment after ischemic stroke in statin users vs nonusers.
    g. Figure S7. Estimated prevalence of all-cause mortality and stroke recurrence by statin timing of initiation.
    h. Figure S8. Estimated prevalence of all-cause mortality and stroke recurrence by statin type.
    i. Figure S9. Estimated prevalence of all-cause mortality and stroke recurrence by statin solubility.
    j. Figure S10. Estimated prevalence of all-cause mortality and stroke recurrence by statin intensity.
    k. Figure S11. Association between increasing statin intensity and all-cause mortality and stroke recurrence after ischemic stroke.

l. Figure S12. Estimated prevalence of all-cause mortality within and after 1 year in cardioembolic/atrial fibrillation stroke patients and patients with low baseline low-density lipoprotein cholesterol.
m. Figure S13. Estimated prevalence of all-cause mortality within and after 1 year in cardioembolic/atrial fibrillation stroke patients and patients with low baseline low-density lipoprotein cholesterol in statin users vs nonusers.
n. Figure S14. Estimated prevalence of stroke recurrence within and after 1 year in cardioembolic/atrial fibrillation stroke patients.
o. Figure S15. Estimated prevalence of stroke recurrence within and after 1 year in cardioembolic/atrial fibrillation stroke patients in statin users vs nonusers.
p. Figure S16. Association between statin use and all-cause mortality within and after 1 year in cardioembolic and atrial-fibrillation related stroke patients and patients with low baseline low-density lipoprotein cholesterol.
q. Figure S17. Association between statin use and stroke recurrence after 1 year in cardioembolic and atrial-fibrillation related stroke patients.
r. Figure S18. Difference in CRP levels (mg/L) within 3-7 days and after 7 days of ischemic stroke between statin users and nonusers.
s. Figure S19. Graphs of Sensitivity Analysis
t. Figure S20. Graphs of Egger's Regression Test
u. Figure S21. Graphs of Funnel Plots

1. **Supplemental Tables**

Table S1. Search Strategy (Keywords/Medical Subject Headings (MeSH) Terms)

Database/ Source	Search Strategy	Filters	Search Date	Hits
PubMed	(statin OR hmg-coa reductase inhibitors OR statins OR hmg-coa reductase inhibitor OR hmg coa reductase inhibitor OR Hydroxymethylglutaryl-CoA reductase inhibitors OR Hydroxymethylglutaryl CoA reductase inhibitors OR atorvastatin OR fluvastatin OR lovastatin OR pravastatin OR pitavastatin OR rosuvastatin OR simvastatin OR lipitor OR caduet OR baycol OR lescol OR mevacor OR altocor OR pravachol OR lipostat OR zocor OR mevinolin OR compactin OR altoprev OR livalo OR flolipid) AND (stroke OR cerebrovascular accident OR brain infarction OR cerebrovascular event)	**Article Type:** Adaptive Clinical Trial, Clinical Study, Clinical Trial, Clinical Trial, Phase III, Clinical Trial, Phase IV, Comparative Study, Controlled Clinical Trial, Meta-Analysis, Multicenter Study, Observational Study, Pragmatic Clinical Trial, Randomized Controlled Trial, Systematic Review<br><br>**Article Language:** English<br><br>**Species:** Humans<br><br>**Age:** 19+ years, 65+ years<br><br>**Publication Date:** 2005 - 2025	28/02/2025	1574
EMBASE	(statin*.tw. OR hmg-coa reductase inhibitor*.tw. OR hydroxymethylglutaryl-CoA reductase inhibitor*.tw. OR atorvastatin.tw. OR fluvastatin.tw. OR lovastatin.tw. OR pravastatin.tw. OR pitavastatin.tw. OR rosuvastatin.tw. OR simvastatin.tw.) AND (stroke*.tw. OR cerebrovascular event.tw. OR cerebrovascular accident.tw. OR cerebrovascular disease.tw. OR brain infarct.tw.)	**Article Type:** Evidence Based Medicine, Meta Analysis, Outcomes Research, Systematic Review, Clinical Trial, Randomized Controlled Trial, Controlled Clinical Trial, Multicenter Study, Phase 3 Clinical Trial, Phase 2 Clinical Trial, Article, Review, Journal, Major Reference Work<br><br>**Article Language:** English Language<br><br>**Species:** Human	01/03/2025	3061

		**Age:** Adults 18-64 years, Aged 65+ years<br><br>**Publication Date:** 2005 - Current		
Scopus	(statin OR hmg-coa reductase inhibitors OR statins OR hmg-coa reductase inhibitor OR hmg coa reductase inhibitor OR Hydroxymethylglutaryl-CoA reductase inhibitors OR Hydroxymethylglutaryl CoA reductase inhibitors OR atorvastatin OR fluvastatin OR lovastatin OR pravastatin OR pitavastatin OR rosuvastatin OR simvastatin OR lipitor OR caduet OR baycol OR lescol OR mevacor OR altocor OR pravachol OR lipostat OR zocor OR mevinolin OR compactin OR altoprev OR livalo OR flolipid) AND (stroke OR cerebrovascular accident OR brain infarction OR cerebrovascular event)	**Limit search to:** Article title, abstract, keywords<br><br>**Article Type:** Articles, Reviews<br><br>**Article Language:** English Language<br><br>**Publication Date:** 2005 – 2025	02/03/2025	839
Web of Science	(statin OR hmg-coa reductase inhibitors OR statins OR hmg-coa reductase inhibitor OR hmg coa reductase inhibitor OR Hydroxymethylglutaryl-CoA reductase inhibitors OR Hydroxymethylglutaryl CoA reductase inhibitors OR atorvastatin OR fluvastatin OR lovastatin OR pravastatin OR pitavastatin OR rosuvastatin OR simvastatin OR lipitor OR caduet OR baycol OR lescol OR mevacor OR altocor OR pravachol OR lipostat OR zocor OR mevinolin OR compactin OR altoprev OR livalo OR flolipid) AND (stroke OR	**Limit search to:** Article title<br><br>**Article Type:** Articles, Reviews<br><br>**Article Language:** English Language<br><br>**Publication Date:** 2005 – 2025	02/03/2025	525

	cerebrovascular accident OR brain infarction OR cerebrovascular event)			
Cochrane Library	(statin OR hmg-coa reductase inhibitors OR statins OR hmg-coa reductase inhibitor OR hmg coa reductase inhibitor OR Hydroxymethylglutaryl-CoA reductase inhibitors OR Hydroxymethylglutaryl CoA reductase inhibitors OR atorvastatin OR fluvastatin OR lovastatin OR pravastatin OR pitavastatin OR rosuvastatin OR simvastatin OR lipitor OR caduet OR baycol OR lescol OR mevacor OR altocor OR pravachol OR lipostat OR zocor OR mevinolin OR compactin OR altoprev OR livalo OR flolipid) AND (stroke OR cerebrovascular accident OR brain infarction OR cerebrovascular event)		01/03/2025	204

Table S2. PRISMA 2020 Checklist

Section and Topic	Item #	Checklist item	Location where item is reported (Page Number)
**TITLE**			
Title	1	Identify the report as a systematic review.	1
**ABSTRACT**			
Abstract	2	See the PRISMA 2020 for Abstracts checklist.	2
**INTRODUCTION**			
Rationale	3	Describe the rationale for the review in the context of existing knowledge.	3
Objectives	4	Provide an explicit statement of the objective(s) or question(s) the review addresses.	4
**METHODS**			
Eligibility criteria	5	Specify the inclusion and exclusion criteria for the review and how studies were grouped for the syntheses.	5
Information sources	6	Specify all databases, registers, websites, organisations, reference lists and other sources searched or consulted to identify studies. Specify the date when each source was last searched or consulted.	4, Supplemental Table S1
Search strategy	7	Present the full search strategies for all databases, registers and websites, including any filters and limits used.	4, Supplemental Table S1
Selection process	8	Specify the methods used to decide whether a study met the inclusion criteria of the review, including how many reviewers screened each record and each report retrieved, whether they worked independently, and if applicable, details of automation tools used in the process.	4
Data collection process	9	Specify the methods used to collect data from reports, including how many reviewers collected data from each report, whether they worked independently, any processes for obtaining or confirming data from study investigators, and if applicable, details of automation tools used in the process.	4
Data items	10a	List and define all outcomes for which data were sought. Specify whether all results that were compatible with each outcome domain in each study were sought (e.g. for all measures, time points, analyses), and if not, the methods used to decide which results to collect.	5
	10b	List and define all other variables for which data were sought (e.g. participant and intervention characteristics, funding sources). Describe any assumptions made about any missing or unclear information.	5
Study risk of bias assessment	11	Specify the methods used to assess risk of bias in the included studies, including details of the tool(s) used, how many reviewers assessed each study and whether they worked independently, and if applicable, details of automation tools used in the process.	6
Effect measures	12	Specify for each outcome the effect measure(s) (e.g. risk ratio, mean difference) used in the synthesis or presentation of results.	6
Synthesis methods	13a	Describe the processes used to decide which studies were eligible for each synthesis (e.g. tabulating the study intervention characteristics and comparing against the planned groups for each synthesis (item #5)).	Table 1
	13b	Describe any methods required to prepare the data for presentation or synthesis, such as handling of missing summary statistics,	6

Section and Topic	Item #	Checklist item	Location where item is reported (Page Number)
		or data conversions.	
	13c	Describe any methods used to tabulate or visually display results of individual studies and syntheses.	6-11
	13d	Describe any methods used to synthesize results and provide a rationale for the choice(s). If meta-analysis was performed, describe the model(s), method(s) to identify the presence and extent of statistical heterogeneity, and software package(s) used.	6
	13e	Describe any methods used to explore possible causes of heterogeneity among study results (e.g. subgroup analysis, meta-regression).	6
	13f	Describe any sensitivity analyses conducted to assess robustness of the synthesized results.	6
Reporting bias assessment	14	Describe any methods used to assess risk of bias due to missing results in a synthesis (arising from reporting biases).	6
Certainty assessment	15	Describe any methods used to assess certainty (or confidence) in the body of evidence for an outcome.	6, 7
**RESULTS**			
Study selection	16a	Describe the results of the search and selection process, from the number of records identified in the search to the number of studies included in the review, ideally using a flow diagram.	6, Figure 1
	16b	Cite studies that might appear to meet the inclusion criteria, but which were excluded, and explain why they were excluded.	N/A
Study characteristics	17	Cite each included study and present its characteristics.	7, Table 1-2
Risk of bias in studies	18	Present assessments of risk of bias for each included study.	11, Supplemental Table S5, Supplemental Figures S19-24
Results of individual studies	19	For all outcomes, present, for each study: (a) summary statistics for each group (where appropriate) and (b) an effect estimate and its precision (e.g. confidence/credible interval), ideally using structured tables or plots.	Table 3-6, Supplemental Tables S6-S7
Results of syntheses	20a	For each synthesis, briefly summarise the characteristics and risk of bias among contributing studies.	6-10
	20b	Present results of all statistical syntheses conducted. If meta-analysis was done, present for each the summary estimate and its precision (e.g. confidence/credible interval) and measures of statistical heterogeneity. If comparing groups, describe the direction of the effect.	6-10, Table 3-6, Supplemental Tables S6-S7
	20c	Present results of all investigations of possible causes of heterogeneity among study results.	6-10, Supplemental Tables S5, Supplemental Figures S19-24

Section and Topic	Item #	Checklist item	Location where item is reported (Page Number)
	20d	Present results of all sensitivity analyses conducted to assess the robustness of the synthesized results.	10, Supplemental Table S5, Supplemental Figures S19-24
Reporting biases	21	Present assessments of risk of bias due to missing results (arising from reporting biases) for each synthesis assessed.	Supplemental Table S5 and S6
Certainty of evidence	22	Present assessments of certainty (or confidence) in the body of evidence for each outcome assessed.	GRADE Table (Table 6)
**DISCUSSION**			
Discussion	23a	Provide a general interpretation of the results in the context of other evidence.	11-13
	23b	Discuss any limitations of the evidence included in the review.	13
	23c	Discuss any limitations of the review processes used.	13
	23d	Discuss implications of the results for practice, policy, and future research.	13
**OTHER INFORMATION**			
Registration and protocol	24a	Provide registration information for the review, including register name and registration number, or state that the review was not registered.	N/A
	24b	Indicate where the review protocol can be accessed, or state that a protocol was not prepared.	N/A
	24c	Describe and explain any amendments to information provided at registration or in the protocol.	N/A
Support	25	Describe sources of financial or non-financial support for the review, and the role of the funders or sponsors in the review.	15
Competing interests	26	Declare any competing interests of review authors.	15
Availability of data, code and other materials	27	Report which of the following are publicly available and where they can be found: template data collection forms; data extracted from included studies; data used for all analyses; analytic code; any other materials used in the review.	15

Table S3. Meta-analysis of Observational Studies in Epidemiology (MOOSE) Checklist

Item No	Recommendation	Location where reported
Reporting of background should include		
1	Problem definition	3-4
2	Hypothesis statement	4
3	Description of study outcome(s)	5
4	Type of exposure or intervention used	5
5	Type of study designs used	Table 1
6	Study population	Table 1
Reporting of search strategy should include		
7	Qualifications of searchers (eg, librarians and investigators)	N/A
8	Search strategy, including time period included in the synthesis and key words	Supplemental Table S1
9	Effort to include all available studies, including contact with authors	Figure 1, Supplemental Table S1
10	Databases and registries searched	Supplemental Table S1
11	Search software used, name and version, including special features used (eg, explosion)	Supplemental Table S1
12	Use of hand searching (eg, reference lists of obtained articles)	Figure 1
13	List of citations located and those excluded, including justification	Figure 1, 6-7
14	Method of addressing articles published in languages other than English	5
15	Method of handling abstracts and unpublished studies	5
16	Description of any contact with authors	N/A
Reporting of methods should include		
17	Description of relevance or appropriateness of studies assembled for assessing the hypothesis to be tested	5
18	Rationale for the selection and coding of data (eg, sound clinical principles or convenience)	5
19	Documentation of how data were classified and coded (eg, multiple raters, blinding and interrater reliability)	5-8
20	Assessment of confounding (eg, comparability of cases and controls in studies where appropriate)	Supplemental Table S5 and S6
21	Assessment of study quality, including blinding of quality assessors, stratification or regression on possible predictors of study results	5
22	Assessment of heterogeneity	6
23	Description of statistical methods (eg, complete description of fixed or random effects models, justification of whether the chosen models account for predictors of study results, dose-response models, or cumulative meta-analysis) in sufficient detail to be replicated	6
24	Provision of appropriate tables and graphics	Tables 1-6, Figures 1-4
Reporting of results should include		

25	Graphic summarizing individual study estimates and overall estimate	Figures 1-4, Supplemental Figures S1-18
26	Table giving descriptive information for each study included	Tables 1-2
27	Results of sensitivity testing (eg, subgroup analysis)	9-10
28	Indication of statistical uncertainty of findings	6-11

Table S4. Modified Jadad Analysis (MJA) for Methodological Quality

Study ID	Author	Criteria 1	Criteria 2	Criteria 3	Criteria 4	Criteria 5	Criteria 6	Criteria 7	Criteria 8	Total
1	Yakusevich et al.	1	1	0	0	0	1	0	1	4
2	Kim et al.	0	0	0	0	0	1	0	1	2
3	Yang et al.	0	0	0	0	0	1	0	1	2
4	Huang et al.	0	0	0	0	1	1	0	1	3
5	Beer et al.	1	1	1	1	1	1	1	1	8
6	Muscari et al.	1	1	1	0	1	1	0	1	6
7	Zare et al.	1	1	1	0	0	1	0	1	5
8	Kang et al.	0	0	0	0	0	1	0	1	2
9	Aivo et al.	0	0	0	0	0	1	1	1	3
10	Flach et al.	0	0	0	0	0	1	0	1	2
11	Han et al.	0	0	0	0	0	1	0	1	2
12	Makihara et al.	0	0	0	0	0	0	0	1	1
13	Milionis et al.	0	0	0	0	0	0	0	1	1
14	O’Brien et al.	0	0	0	0	0	1	1	1	3
15	Zhang et al.	1	0	0	0	0	1	1	1	4
16	Jia & Zhou	0	0	0	0	1	1	0	1	3
17	Arevalo-Lorido et al.	0	0	0	0	1	1	0	1	3
18	Bao et al.	0	0	0	0	0	1	0	1	2
19	Ni Chroinin et al.	0	0	0	0	0	1	0	1	2
20	Montaner et al.	1	1	1	1	1	1	1	1	8
21	Cappellari et al.	0	0	0	0	0	1	0	1	2
22	Cui et al.	0	0	0	0	1	1	0	1	3
23	Cui et al.	0	0	0	0	1	1	1	1	4
24	Scheitz et al.	0	0	0	0	0	0	0	1	1
25	Furlan et al.	0	0	0	0	0	0	0	1	1
26	Winardi et al.	0	0	0	0	0	1	0	1	2
27	Song et al.	0	0	0	0	0	1	0	1	2
28	Hjalmarsson et al.	0	0	0	0	0	0	0	1	1
29	Sakurai et al.	0	0	0	0	0	1	0	1	2
30	Ueno et al.	1	0	0.5	0	0	1	1	1	4.5
31	Flint et al.	0	0	0	0	0	1	0	1	2
32	Vitturi & Gagliardi	0	0	0	0	1	1	0	1	3

33	Choi et al.	0	0	0	0	0	1	1	1	3
34	Lin et al.	0	0	0	0	0	1	0	1	2
35	Ntaois et al.	0	0	0	0	0	1	0	1	2
36	Choi et al.	0	0	0	0	0	1	0	1	2
37	Choi et al.	0	0	0	0	0	1	0	1	2
38	Vitturi & Gagliardi	0	0	0	0	1	1	0	1	3
39	Gong et al.	0	0	0	0	0	1	1	1	3
40	Park et al.	0	0	0	0	0	1	0	1	2
41	Marvardi et al.	0	0	0	0	0	1	0	1	2
42	Wu et al.	0	0	0	0	0	1	1	1	3
43	Kim et al.	0	0	0	0	0	1	0	1	2
44	Lee et al.	0	0	0	0	0	0	0	1	1
45	Song et al.	0	0	0	0	1	1	0	1	3
46	Yang et al.	1	1	1	1	1	1	1	1	8
47	Chen et al.	1	1	1	0	1	1	1	1	7
48	Bach et al.	0	0	0	0	0	1	0	1	2
49	Kyto et al.	0	0	0	0	0	0	0	1	1
50	Kim et al.	0	0	0	0	0	1	0	1	2
51	Cao et al.	1	1	0	0	0	1	0	1	4

Criteria 1: Was the study randomised? (0 = not described or no, 1 = yes)
Criteria 2: Was the method of randomisation appropriate (0 = not described or no, 1 = yes)
Criteria 3: Was the study described as being blinded? (0 = not described or no, 0.5 = single blinded 1 = double-blinded)
Criteria 4: Was the method of blinding appropriate (0 = not described or no, 1 = yes)
Criteria 5: Was there a description of withdrawals and dropouts? (0 = not described or no, 1 = yes)
Criteria 6: Was there a clear description of the inclusion/exclusion criteria? (0 = not described or no, 1 = yes)
Criteria 7: Was the method used to assess adverse events described? (0 = not described or no, 1 = yes)
Criteria 8: Was the method of statistical analysis described? (0 = not described or no, 1 = yes)

<u>Assessment of quality based on MJA score</u>
3 or more = high quality
2 or less = low quality

Table S5. ROBINS-I: Risk of Bias in Non-Randomized Studies of Interventions

Study (year)	Confounding*	Selection of participants	Classification of interventions	Deviations from intended interventions	Missing data	Measurement of outcomes	Selection of reported result	Overall risk of bias
Kim et al. (2024)	Moderate	Low	Low	Low	NI	Low	Low	Moderate
Yang et al. (2022)	Low	Low	Low	Low	Low	Low	Low	Low
Huang et al. (2015)	Moderate	Moderate	Moderate	NI	Moderate	Moderate	Moderate	Moderate
Aivo et al. (2023)	Low	Low	Low	Serious	Moderate	Low	Low	Serious
Flach et al. (2019)	Moderate	Moderate	Low	Serious	NI	Low	Low	Serious
Han et al. (2021)	Moderate	Moderate	Moderate	Moderate	Moderate	Moderate	Low	Moderate
Makihara et al. (2013)	Moderate	Moderate	Moderate	Moderate	NI	Low	Low	Moderate
Milionis et al. (2009)	Moderate	Low	Moderate	NI	NI	Low	Low	Moderate
O'Brien et al. (2015)	Low	Low	Low	Serious	NI	Low	Low	Serious
Jia & Zhou, 2013	Moderate	Low	Low	Moderate	Moderate	Low	Low	Moderate
Arevalo-Lorido et al. (2014)	Moderate	Low	Moderate	NI	Moderate	Low	Low	Moderate
Bao et al. (2023)	Low	Low	Low	NI	Low	Low	Low	Low
Ni Chroinin et al. (2011)	Moderate	Moderate	Low	NI	Low	Low	Low	Moderate
Cappellari et al. (2013)	Moderate	Moderate	Low	NI	NI	Moderate	Moderate	Moderate
Cui et al. (2022)	Moderate	Moderate	Low	NI	Moderate	Moderate	Low	Moderate

Cui et al. (2021)	Moderate	Moderate	Low	NI	Moderate	Moderate	Low	Moderate
Scheitz et al. (2015)	Low	Low	Moderate	NI	Moderate	Low	Low	Moderate
Furlan et al. (2020)	Moderate	Moderate	Low	NI	Low	Low	Low	Moderate
Winardi et al. (2024)	Moderate	Moderate	Moderate	NI	NI	Low	Low	Moderate
Song et al. (2014)	Low	Low	Low	Moderate	Moderate	Moderate	Low	Moderate
Hjalmarsson et al. (2012)	Moderate	Moderate	Moderate	NI	NI	Moderate	Low	Moderate
Sakurai et al. (2011)	Serious	Low	Low	Moderate	NI	Low	Low	Serious
Flint et al. (2011)	Low	Low	Low	NI	NI	Moderate	Low	Moderate
Vitturi & Gagliardi (2020)	Moderate	Moderate	Low	Low	Low	Low	Low	Moderate
Choi et al. (2019)	Moderate	Moderate	Low	Moderate	NI	Moderate	Low	Moderate
Lin et al. (2019)	Serious	Moderate	Moderate	NI	NI	Moderate	Low	Serious
Ntaios et al. (2014)	Moderate	Low	Moderate	NI	Moderate	Low	Moderate	Moderate
Choi et al. (2014)	Serious	Serious	Moderate	NI	Low	Moderate	Moderate	Serious
Choi et al. (2024)	Low	Low	Low	NI	NI	Moderate	Low	Moderate
Vitturi & Gagliardi (2019)	Moderate	Low	Moderate	NI	Moderate	Low	Moderate	Moderate
Gong et al. (2023)	Low	Low	Low	NI	NI	Low	Low	Low
Park et al. (2020)	Low	Low	Moderate	Moderate	NI	Low	Low	Moderate

Marvardi et al. (2025)	Moderate	Moderate	Moderate	Moderate	Moderate	Low	Low	Moderate
Wu et al. (2017)	Moderate	Moderate	Low	NI	NI	Low	Low	Moderate
Kim et al. (2023)	Low	Moderate	Moderate	NI	NI	Moderate	Low	Moderate
Lee et al. (2024)	Serious	Moderate	Serious	NI	NI	Moderate	Moderate	Serious
Song et al. (2015)	Low	Low	Moderate	NI	Moderate	Moderate	Moderate	Moderate
Bach et al. (2023)	Low	Low	Low	Low	Low	Low	Low	Low
Kyto et al. (2024)	Low	Moderate	Moderate	Moderate	NI	Moderate	Moderate	Moderate
Kang et al. (2015)	Moderate	Moderate	Low	NI	Moderate	Low	Low	Moderate
Kim et al. (2025)	Low	Low	Low	NI	Moderate	Low	Low	Moderate

Judgement scale: Low, Moderate, Serious, Critical, or NI (No information).

Rule of thumb: Overall = the worst domain (unless clearly implausible).

*Prespecified key confounders for statins-after-stroke: age, sex, stroke subtype (TOAST classification), baseline NIHSS, prior statin use, LDL-C, diabetes, hypertension, AF, smoking, reperfusion therapy (IVT/EVT), time-to-treatment, and year of enrolment/care model.

Two reviewers (MG and SB) independently rated ROBINS-I; disagreements resolved by consensus/third reviewer. We considered time-varying confounding (e.g., treatment escalation) and immortal time bias in classification/deviation domains. When outcome assessors were unblinded and outcomes were subjective (e.g., PSCI), we rated “Measurement of outcomes” ≥ Moderate.

Table S6. RoB 2: Risk of Bias in Randomized Trials

Trial (year)	Randomization process	Deviations from intended interventions (effect of assignment)	Missing outcome data	Measurement of the outcome	Selection of the reported result	Overall risk of bias
Yakusevich et al. (2012)	Low	Low	Low	Low	Low	Low
Beer et al. (2012)	Low	Some	Low	Low	Some	High
Muscari et al. (2011)	Low	Some	Some	Low	Low	High
Zare et al. (2012)	Low	Low	Low	Low	Low	Low
Zhang et al. (2018)	Some	Some	Low	Low	Low	High
Montaner et al. (2016)	Low	Some	Some	Low	Some	High
Ueno et al. (2015)	Low	Some	Some	Low	Low	High
Yang et al. (2021)	Low	Some	Some	Low	Low	High
Chen et al. (2018)	Low	Low	Low	Low	Low	Low

Cao et al. (2017)	Low	Low	Low	Low	Low	Low

Judgement scale: Low risk, Some concerns, High risk.

Rules of thumb: If Randomization process is High → Overall often High; If ≥2 domains are Some concerns → Overall typically High (per recent guidance).

We used the RoB 2 (parallel-group, effect of assignment) tool with signaling questions; two reviewers rated independently. For blinded outcomes (e.g., mortality) measured from registries, “Measurement” is usually Low; for PSCI with unblinded assessors, consider Some concerns/High. We verified selective reporting by comparing prespecified outcomes (protocol/registry) with published endpoints.

Table S7. Funding Bias Scores for Studies

Study ID	Author	Publication Bias	Funding
1	Yakusevich et al.	0	
2	Kim et al.	1	Ministry of Science and ICT (RS-2023-00208062
3	Yang et al.	2	National Institute for Health Research (NIHR) School of Primary Care Research [SPCR-2014-10043, reference number 340], Cambridge Commonwealth, European and International Trust, NIH/NIA R03AG070661
4	Huang et al.	1	Science and Technology Program of Beijing, China
5	Beer et al.	2	NHMRC Centre of Clinical Research Excellence – Centre for Training in Clinical Cerebrovascular and Cardiovascular Research, A Pfizer CVL grant for CT scans and pharmaceuticals, UWA Research Grant funded S100B assays, UWA Small Bequest Research Grant funded isoprostane and CRP assays. 'Oscar 2' ambulatory blood pressure monitors were donated by SunTech Medical.
6	Muscari et al.	2	Pfizer Italia S.R.L
7	Zare et al.	0	
8	Kang et al.	1	Korea Health 21 R&D project, Ministry of Health and Welfare, Korea
9	Aivo et al.	2	Finnish Foundation for Cardiovascular Research, Finnish Cultural Foundation, the Paulo Foundation, the Paavo Nurmi Foundation, and the Finnish Governmental VTR-funding
10	Flach et al.	2	National Institute for Health Research Collaboration for Leadership in Applied Health Research and Care South London at King's College Hospital NHS Foundation Trust, Royal College of Physicians, as well as the support from the National Institute for Health Research Biomedical Research Centre based at Guy's and St Thomas' NHS Foundation Trust and King's College London
11	Han et al.	0	
12	Makihara et al.	1	Grant-in-Aid for Scientific Research (A 22249069) and the Coordination, Support and Training Program for Translational Research from the Japanese Ministry of Education, Culture, Sports, Science and Technology.
13	Milionis et al.	0	
14	O'Brien et al.	1	Patient-Centered Outcomes Research Institute
15	Zhang et al.	0	
16	Jia & Zhou	1	Wu Jieping Medical Foundation Special Assistance Fund to Clinical Research
17	Arevalo-Lorido et al.	0	
18	Bao et al.	2	Natural Science Foundation of China (No. 81971162), China Postdoctoral Science Foundation (Nos. 2020M673248 and 2021M692294), Sichuan Science and Technology Program (No. 2021YJ0437)
19	Ni Chroinin et al.	2	Irish Health Services Executive, National Lottery of Ireland, and an unrestricted educational grant for stroke research and education from Servier
20	Montaner et al.	2	EC07/90195 "Strategies to improve safety and efficacy of Simvastatin in the acute phase of stroke: STARS (Stroke Treatment With Acute Reperfusion and Simvastatin) trial, "Consorcio de Apoyo a la Investigación Biomédica en Red (CAIBER) 1546-C-161", Spanish stroke research network INVICTUS (RD12/0014/0005), Multi-PART (Multicentre Preclinical Animal Research Team; FP7 Grant Agreement HEALTH-F2-2013–603043)
21	Cappellari et al.	0	Nonfinancial auspices of the Italian Stroke Association
22	Cui et al.	0	

23	Cui et al.	2	National Key R&D Program of China (Funding number: 2018YFC1311400 and 2018YFC1311401), National Natural Science Foundation of China (Funding number: 81772435), Post-Doctor Research Project (Funding number: 2020HXBH032)
24	Scheitz et al.	1	Volkswagen Stiftung (Lichtenberg program to ME), Deutsche Forschungsgemeinschaft and German Ministry of Education and Research (Center for Stroke Research Berlin, CSB)
25	Furlan et al.	0	
26	Winardi et al.	0	
27	Song et al.	0	
28	Hjalmarsson et al.	0	
29	Sakurai et al.	2	Grant-in-Aid for Scientific Research (C) from the Japan Society for the Promotion of Science, Ministry of Education, Culture, Sports, Science and Technology
30	Ueno et al.	3	AstraZeneca K.K. participated in the preparation of the study design.
31	Flint et al.	2	Centers for Disease Control and Prevention, Kaiser Permanente Community Benefits Research Fund
32	Vitturi & Gagliardi	0	
33	Choi et al.	1	National Research Foundation of Korea: NRF-2019M3A9E8020261, Korean Government, Korean Neurological Association: KNA-17-MI-10
34	Lin et al.	0	
35	Ntaois et al.	0	
36	Choi et al.	1	Korean Stroke Society Young Investigator’s Award (KSS-2009-003), Korea University Grant (K1032861)
37	Choi et al.	1	Conflicts of interest reported
38	Vitturi & Gagliardi	0	
39	Gong et al.	2	National Natural Science Foundation of China (No. 82001264), Chongqing Technology Innovation and Application Development Project
40	Park et al.	2	Korea Healthcare Technology R&D Project, Ministry for Health and Welfare, Republic of Korea (HI10C2020), by a fund (code 2017ER620100) from the Research of Korea Centers for Disease Control and Prevention, and by a grant from Yuhan corporation
41	Marvardi et al.	1	
42	Wu et al.	2	Chang Gung Memorial Hospital and National Science Council, Taiwan (CMRPG6B0111, CMRPG6B0112, and NSC 102–2628-B-182–012)
43	Kim et al.	2	(2023-ER1006-00) from the Research of Korea Centers for Disease Control and Prevention. This study was supported by a grant (BCRI22032) from the Chonnam National University Hospital Biomedical Research Institute
44	Lee et al.	0	
45	Song et al.	0	
46	Bach et al.	2	Department of Clinical Epidemiology, Aarhus University and Aarhus University Hospital
47	Kyto et al.	2	Finnish Foundation for Cardiovascular Research sr, Finnish State research funding
48	Yang et al.	2	National Natural Science Foundation of China (No. 81171084, 81671167, and 81801150), the Guangzhou Science and Technology Program of China (No. 2014Y2-00505), the Science and Technology Program of Guangdong, China (No. 2014A030313384, 201508020004 and 2017A020215049), Guangdong Natural Science Foundation (2018A0303130182)
49	Chen et al.	2	Youth Fund of Health Department of Fujian Province in 2012

50	Kim et al.	2	Korea National Institute of Health research project (No. 2023-ER-1006-01), (BCRI24042) from the Chonnam National University Hospital Biomedical Research Institute.
51	Cao et al.	0	

0 = Low potential for bias
1 = Conflicts of interest declared relating to industry funding outside of current research publication
2 = Funded by industry
3 = High potential for bias

Table S8. Pooled prevalence of clinical outcomes in ischemic stroke patient subgroups with post-stroke statin use vs no post-stroke statin use

Subgroup	Outcome	Timeframe	Study Type	Overall							Statin nonusers							Statin users						
				Summary Effects		Heterogeneity				Heterogeneity Variance Estimates	Summary Effects		Heterogeneity				Heterogeneity Variance Estimates	Summary Effects		Heterogeneity				Heterogeneity Variance Estimates
				Pooled Prevalence (95% CI)	Test of ES = 0	Chi-squared	H	I^2 (%)	P-value	$t^2 \leq^{F}$	Pooled Prevalence (95% CI)	Test of ES = 0	Chi-squared	H	I^2 (%)	P-value	$t^2 \leq^{F}$	Pooled Prevalence (95% CI)	Test of ES = 0	Chi-squared	H	I^2 (%)	P-value	$t^2 \leq^{F}$
**Cardioembolic/ AF-related stroke**	**All-Cause Mortality**	≤ 1 year	Retrospective	0.21 [0.21, 0.22]	z = 204.99, p < 0.01	-	-	-	-	-	0.22 [0.21, 0.22]	z = 188.80, p < 0.01	-	-	-	-	-	0.17 [0.16, 0.18]	z = 58.06, p < 0.01	-	-	-	-	-
			Prospective	0.28 [0.27, 0.30]	z = 59.17, p < 0.01	-	-	-	-	-	0.36 [0.33, 0.39]	z = 40.09, p < 0.01	-	-	-	-	-	0.24 [0.22, 0.26]	z = 43.21, p < 0.01	-	-	-	-	-
			**Overall**	**0.23 [0.18, 0.28]**	**z = 15.74,**	**69.72**	**-**	**97.13**	**<0.01**	**0.01**	**0.30 [0.19, 0.42]**	**z = 8.66,**	**101.67**	**-**	**98.03**	**<0.01**	**0.05**	**0.19 [0.14, 0.24]**	**z = 12.97,**	**41.78**	**-**	**95.21**	**<0.01**	**0.01**

					**p < 0.01**							**p < 0.01**							**p < 0.01**					
			Retrospective	0.10 [0.04, 0.17]	z = 5.24, p < 0.01	-	285.80	98.95	<0.01	-	0.13 [0.05, 0.24]	z = 4.54, p < 0.01	-	201.55	98.51	<0.01	-	0.06 [0.02, 0.12]	z = 4.19, p < 0.01	-	131.85	97.72	-	-
		> 1 year	Prospective	0.01 [0.00, 0.06]	z = 1.42, p = 0.16	-	-	-	-	-	0.00 [0.00, 0.19]	z = 0.00, p = 1.00	-	-	-	-	-	0.01 [0.00, 0.07]	z = 1.42, p < 0.01	-	-	-	-	-
			**Overall**	**0.10 [0.04, 0.17]**	**z = 4.94, p < 0.01**	-	**292.79**	**98.63**	**<0.01**	**0.05**	**0.10 [0.03, 0.20]**	**z = 3.95, p < 0.01**	-	**203.12**	**98.03**	**<0.01**	**0.09**	**0.05 [0.02, 0.10]**	**z = 4.07, p < 0.01**	-	**136.60**	**97.07**	**<0.01**	**0.04**
		≤ 1 year	-	**0.06 [0.05, 0.06]**	**z = 31.22, p < 0.01**	**-**	**-**	**-**	**-**	**-**	**0.06 [0.05, 0.07]**	**z = 20.53, p < 0.01**	**-**	**-**	**-**	**-**	**-**	**0.05 [0.04, 0.06]**	**z = 22.78, p < 0.01**	**-**	**-**	**-**	**-**	**-**
	**Stroke Recurrence**		Retrospective	0.13 [0.11, 0.16]	z = 17.29, p <0.01	-	57.18	94.75	<0.01	-	0.15 [0.13, 0.17]	z = 24.55, p < 0.01	-	18.29	83.59	<0.01	-	0.12 [0.07, 0.17]	z = 8.77, p < 0.01	-	60.83	95.07	<0.01	-
		> 1 year	Prospective	0.06 [0.05-0.07]	z = 20.91, p <0.01	-	-	-	-	-	0.06 [0.04, 0.08]	z = 8.88, p = 1.00	-	-	-	-	-	0.06 [0.05, 0.07]	z = 17.40, p = 1.00	-	-	-	-	-

			**Overall**	**0.13 [0.10, 0.18]**	**z = 11.60, p < 0.01**	**-**	**250.23**	**98.00**	**<0.01**	**0.02**	**0.13 [0.10, 0.16]**	**z = 13.35, p < 0.01**	**-**	**76.35**	**93.45**	**<0.01**	**0.01**	**0.12 [0.08, 0.17]**	**z = 8.94, p < 0.01**	**-**	**154.65**	**96.77**	**<0.01**	**0.03**
**Low Baseline LDL-Cholesterol Levels**	**All-Cause Mortality**	-	-	**0.11 [0.07-0.16]**	**z = 8.94, p < 0.01**	**42.45**	**-**	**95.29**	**<0.01**	**0.01**	**0.17 [0.11, 0.23]**	**z = 9.68, p < 0.01**	**27.70**	**-**	**92.78**	**<0.01**	**0.02**	**0.06 [0.04, 0.09]**	**z = 8.18, p < 0.01**	**9.92**	**-**	**79.83**	**0.01**	**0.01**

Abbreviations: CI, confidence interval; ES, effect size; H, heterogeneity statistic; RCT, randomized controlled trial; AF, atrial fibrillation; LDL, low-density lipoprotein

Table S9. Summary effects and heterogeneity obtained from meta-analysis of statin use and clinical outcomes after ischemic stroke in sub-groups

Sub-group	Outcome	Time frame	N	n	Effect Measure	Study Type	Summary Effects		Heterogeneity				Heterogeneity Variance Estimates
							OR (95% CI)	Test of Overall Effect	Cochran's Q	H	I^2 (%)	P-value	t^2≤^{F}
Cardioembolic/ AF-related stroke	All-Cause Mortality	≤ 1 year	2	48,932	OR	Retrospective	0.52 [0.24, 1.13]	z = -1.64, p = 0.10	9.69	-	89.7	<0.01	0.28
			1	2888		Prospective	0.57 [0.48, 0.67]	z = -6.71, p < 0.01	0.00		-	-	-
			**3**	**51,280**		**Overall**	**0.57 [0.42, 0.78]**	**z = -3.49, p < 0.01**	**17.42**	**2.95**	**88.5**	**<0.01**	**0.06**
		> 1 year	4	23,994	OR	Retrospective	0.46 [0.24, 0.88]	z = -2.36, p = 0.02	42.02	-	92.9	<0.01	0.31
			1	91		Prospective	0.77 [0.03, 19.57]	z = -0.16, p = 0.87	0.00		-	-	-
			**5**	**24,085**		**Overall**	**0.47 [0.25, 0.88]**	**z = -2.37, p = 0.02**	**42.03**	**3.24**	**90.5**	**<0.01**	**0.30**
	Stroke Recurrence		Insufficient studies										
		> 1 year	4	26,479	OR	Retrospective	0.82 [0.57, 1.17]	z = -1.10, p = 0.27	27.62	-	89.1	<0.01	0.09
			2	2,244		Prospective	0.89 [0.61, 1.30]	z = -0.60, p = 0.55	0.03		0.0	0.85	-
			**6**	**28,723**		**Overall**	**0.84 [0.63, 1.12]**	**z = -1.22, p = 0.22**	**28.20**	**2.38**	**82.3**	**<0.01**	**0.08**
Low baseline cholesterol levels	All-Cause Mortality	Any	**3**	**4,824**	**OR**	-	**0.32 [0.17, 0.58]**	**z = -3.71, p <0.001**	**13.88**	**2.63**	**85.6**	**0.01**	**0.24**

Abbreviations: OR, odds ratio; CI, confidence interval; AF, atrial fibrillation; N, number of studies; n, number of patients

Table S10. Outputs of Peters' test

Analysis		Number of Studies	Peters' test slope					Peters' test constant					Test of H0
			Coefficient	Standard Error	t	P>|t|	95% CI	Coefficient	Standard Error	t	P>|t|	95% CI	P-value
Dementia/CI		3	235.81	290.12	0.81	0.57	-3450.55, -3922.18	-0.26	0.09	-2.85	0.22	-1.43, 0.90	0.57
Association between Statin Use and All-Cause Mortality:	Within 3 months of IS	9*	-13.04	47.54	-0.27	0.79	-125.47, 99.38	-1.10	0.10	-10.99	0.00	-1.33, -0.86	0.79
	Within 1 year of IS	13*	-120.05	87.01	-1.38	0.20	-311.56, 71.45	-0.72	0.08	-9.08	0.00	-0.89, -0.54	0.20
	After 1 year of IS	8	-238.31	355.84	-0.67	0.53	-1109.03, 632.40	-0.34	0.08	-4.13	0.01	-0.55, -0.14	0.53
	Within 1 year of CE/AF-stroke	3	-481.36	173.81	-2.77	0.22	-2689.97, 1727.16	-0.29	0.04	-8.03	0.08	-0.74, 0.17	0.22
	After 1 year of CE/AF-stroke	4*	-858.68	229.49	-3.74	0.07	-1946.10, 128.74	-0.02	0.12	-0.17	0.88	-0.52, 0.48	0.07
	IS patients with low baseline LDL-C	3	1231.91	169.77	7.26	0.09	-925.21, 3389.03	-2.11	0.13	-16.57	0.04	-3.73, -0.49	0.09
Association between Statin Use and Stroke Recurrence:	Within 1 year of IS	4*	-11.73	40.12	-0.29	0.80	-184.33, 160.88	0.26	0.04	-6.58	0.02	-0.43, -0.09	0.80
	After 1 year of IS	8	-351.84	87.38	-4.03	0.01	-565.65, 138.02	-0.04	0.02	-1.60	0.16	-0.10, 0.02	0.01

	**Within 1 year of CE/AF-stroke**	Insufficient studies										
	**After 1 year of CE/AF-stroke**	6	10.21	111.00	0.09	0.93	-297.97, 318.39	-0.26	0.10	-2.66	0.06	-0.54, 0.01	0.93
**Association between Increasing Statin Intensity and:**	**All-Cause Mortality**	6	20.81	356.65	0.06	0.96	-969.41, 1011.03	-0.15	0.07	-2.15	0.10	-0.35, 0.04	0.96
	**Stroke Recurrence**	4	-56.62	447.62	-0.13	0.91	-1982.55, 1869.31	-0.001	0.10	-0.01	0.99	-0.41, 0.41	0.91

*Study with zero-events excluded from bias analysis

Table S10. Outputs of Egger's test

<table>
<tr><th colspan="2" rowspan="2">Analysis</th><th rowspan="2">Number of Studies</th><th colspan="5">Egger test slope</th><th colspan="5">Egger test constant</th><th>Test of H0</th></tr>
<tr><th>Coefficient</th><th>Standard Error</th><th>t</th><th>P>|t|</th><th>95% CI</th><th>Coefficient</th><th>Standard Error</th><th>t</th><th>P>|t|</th><th>95% CI</th><th>P-value</th></tr>
<tr><td colspan="2">Dementia/CI</td><td>3</td><td>-0.34</td><td>0.23</td><td>-1.47</td><td>0.38</td><td>-3.24, 2.56</td><td>2.00</td><td>4.97</td><td>0.40</td><td>0.76</td><td>-61.15, 65.17</td><td>0.76</td></tr>
<tr><td rowspan="6">Association between Statin Use and All-Cause Mortality:</td><td>Within 3 months of IS</td><td>10</td><td>-1.22</td><td>0.14</td><td>-8.51</td><td>0.00</td><td>-1.55, -0.89</td><td>0.45</td><td>0.59</td><td>0.76</td><td>0.47</td><td>-0.91, 1.81</td><td>0.47</td></tr>
<tr><td>Within 1 year of IS</td><td>14</td><td>-0.62</td><td>0.08</td><td>-7.41</td><td>0.00</td><td>-0.81, -0.44</td><td>-1.67</td><td>0.87</td><td>-1.93</td><td>0.08</td><td>-3.57, 0.22</td><td>0.08</td></tr>
<tr><td>After 1 year of IS</td><td>8</td><td>-0.30</td><td>0.10</td><td>-2.89</td><td>0.03</td><td>-0.56, -0.05</td><td>-1.76</td><td>3.21</td><td>-0.55</td><td>0.60</td><td>-9.61, 6.09</td><td>0.60</td></tr>
<tr><td>Within 1 year of CE/AF-stroke</td><td>3</td><td>-0.13</td><td>0.07</td><td>-1.72</td><td>0.34</td><td>-1.06, 0.80</td><td>-4.35</td><td>1.17</td><td>-3.71</td><td>0.17</td><td>-19.23, 10.54</td><td>0.17</td></tr>
<tr><td>After 1 year of CE/AF-stroke</td><td>5</td><td>0.05</td><td>0.21</td><td>0.23</td><td>0.83</td><td>-0.62, 0.72</td><td>-2.68</td><td>1.76</td><td>-1.52</td><td>0.23</td><td>-8.28, 2.92</td><td>0.23</td></tr>
<tr><td>IS patients with low baseline LDL-C</td><td>3</td><td>-2.75</td><td>0.31</td><td>-8.90</td><td>0.07</td><td>-6.67, 1.17</td><td>7.80</td><td>1.65</td><td>4.72</td><td>0.13</td><td>-13.19, 28.80</td><td>0.13</td></tr>
<tr><td rowspan="2">Association between Statin Use and Stroke Recurrence:</td><td>Within 1 year of IS</td><td>4*</td><td>-0.25</td><td>0.03</td><td>-7.11</td><td>0.02</td><td>-0.40, -0.10</td><td>-0.39</td><td>0.51</td><td>-0.77</td><td>0.52</td><td>-2.57, 1.79</td><td>0.52</td></tr>
<tr><td>After 1 year of IS</td><td>8</td><td>0.02</td><td>0.03</td><td>0.82</td><td>0.44</td><td>-0.05,</td><td>-2.78</td><td>0.64</td><td>-4.31</td><td>0.01</td><td>-4.35,</td><td>0.01</td></tr>
</table>

							0.10					-1.20	
	**Within 1 year of CE/AF-stroke**	Insufficient studies										
	**After 1 year of CE/AF-stroke**	6	-0.30	0.14	-2.22	0.09	-0.68, 0.08	0.76	1.52	0.50	0.64	-3.45, 4.98	0.64
**Association between Increasing Statin Intensity and:**	**All-Cause Mortality**	6	-0.14	0.10	-1.40	0.24	-0.43, 0.14	0.86	2.43	0.35	0.74	-5.88, 7.59	0.74
	**Stroke Recurrence**	4	-0.05	0.20	-0.25	0.82	-0.92, 0.81	2.00	4.46	0.45	0.70	-17.18, 21.18	0.70

*Study with zero-events excluded from bias analysis

2. Supplemental Figures

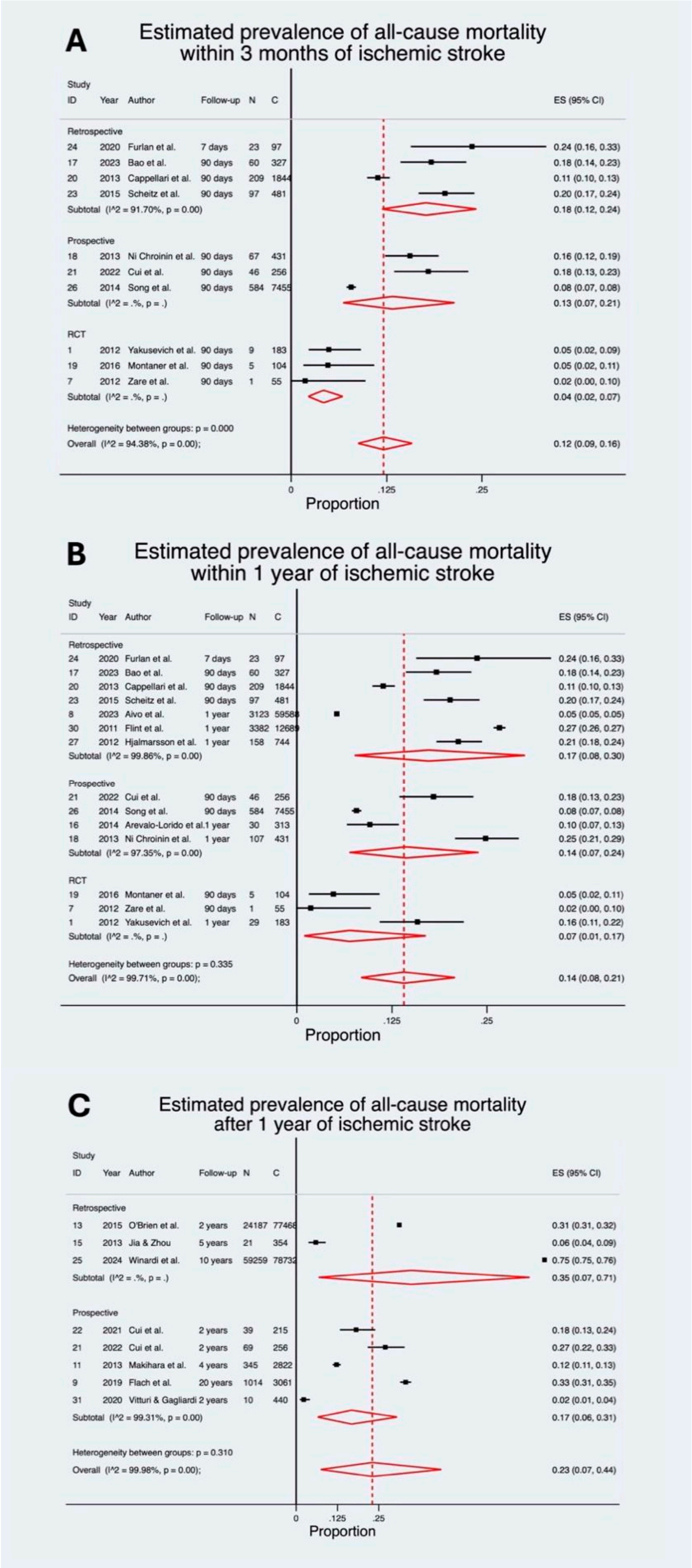

Figure S1. Estimated prevalence of all-cause mortality: (A) within 3 months; (B) 1 year and; (C) after 1 year of ischemic stroke.

Abbreviations: N, number of patients who died; C, total number of patients; ES, effect size; p, *p*-value

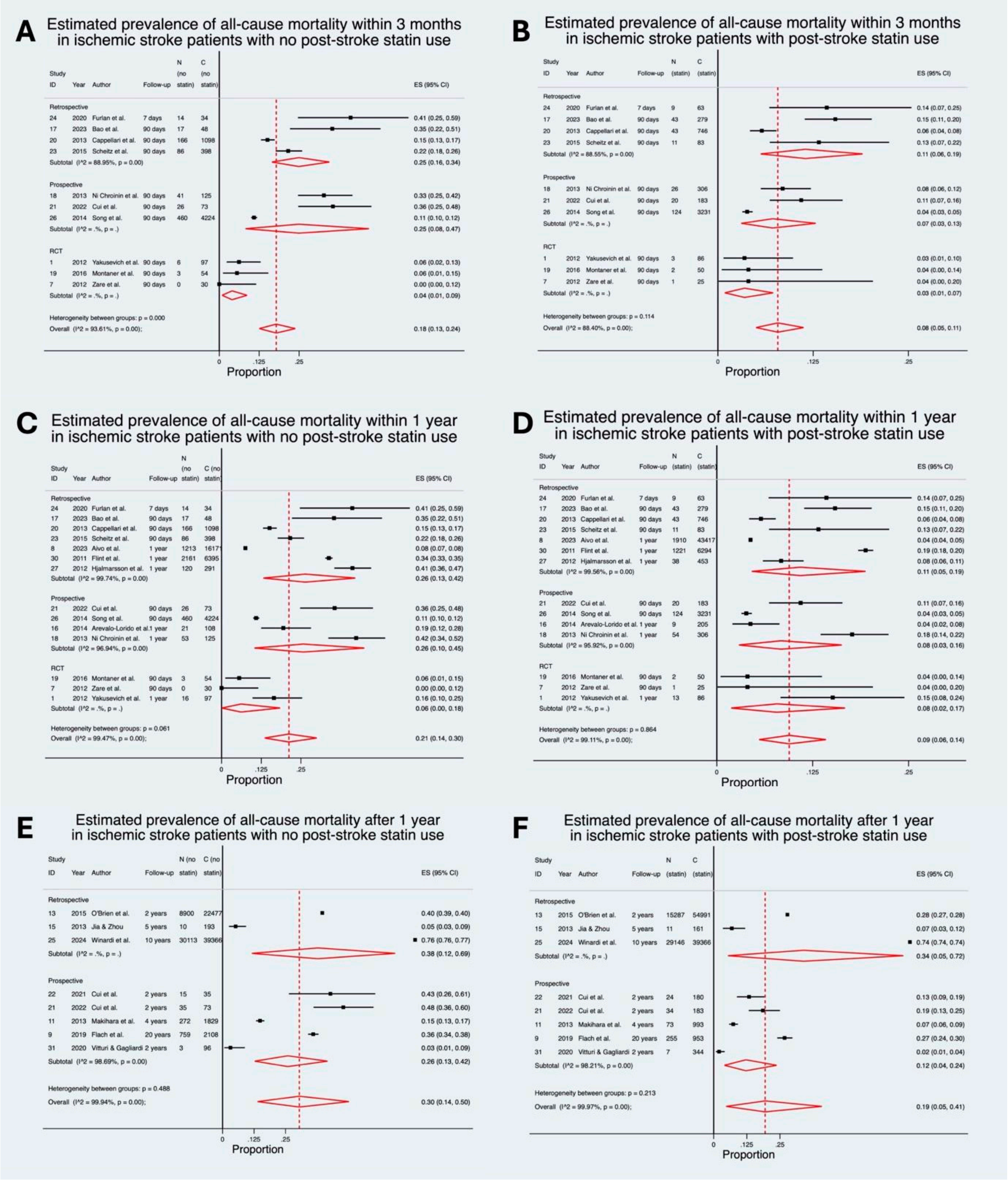

Figure S2. Estimated prevalence of all-cause mortality within (A, B) 3 months; (C, D) 1 year and; (E, F) after 1 year of ischemic stroke in statin users vs nonusers

Abbreviations: N, number of patients who died; C, total number of patients; ES, effect size; p, *p*-value

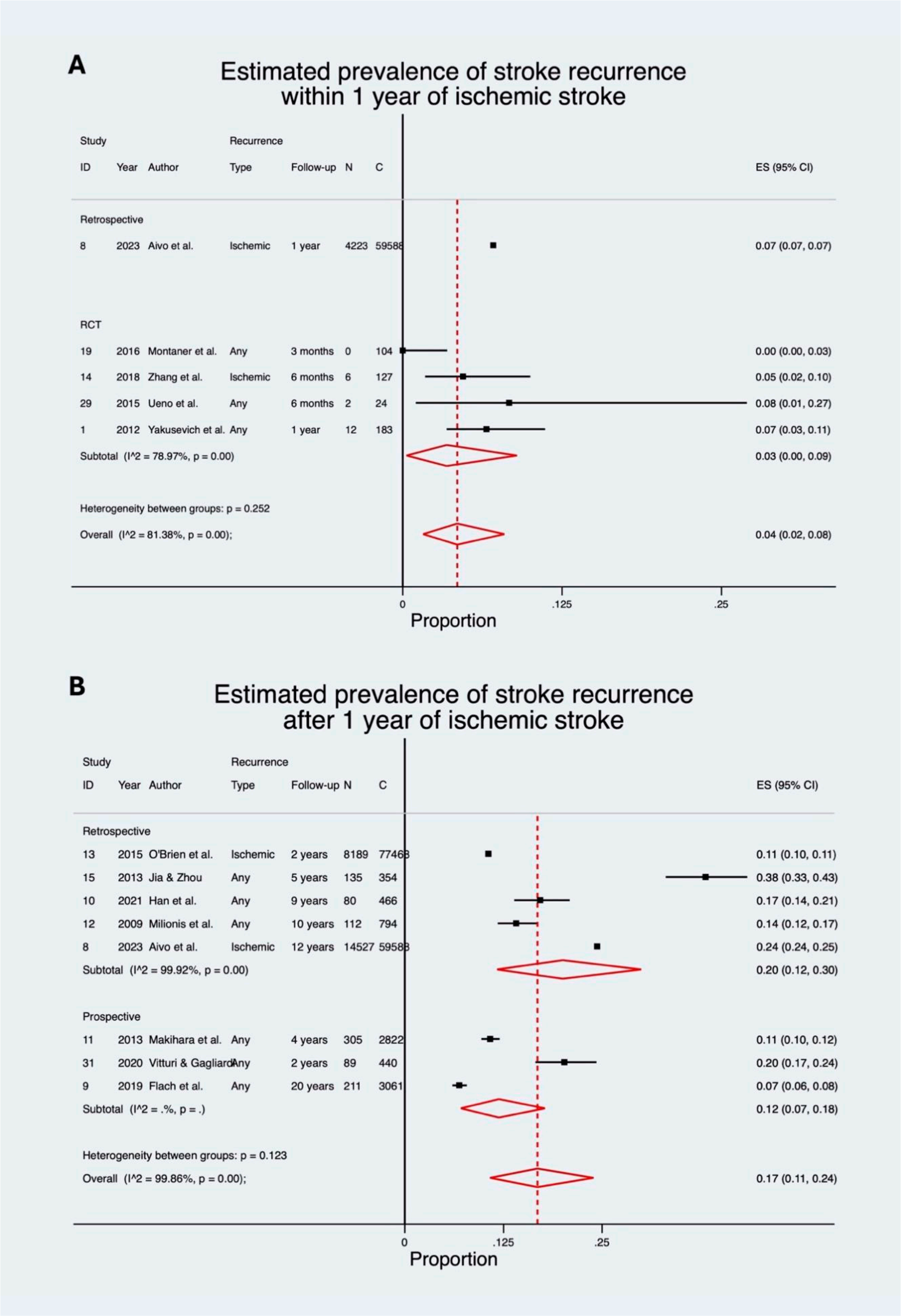

Figure S3. Estimated prevalence of stroke recurrence within: (A) 1 year and; (B) after 1 year of ischemic stroke.

Abbreviations: N, number of patients who had a recurrent stroke; C, total number of patients; ES, effect size; p, *p*-value

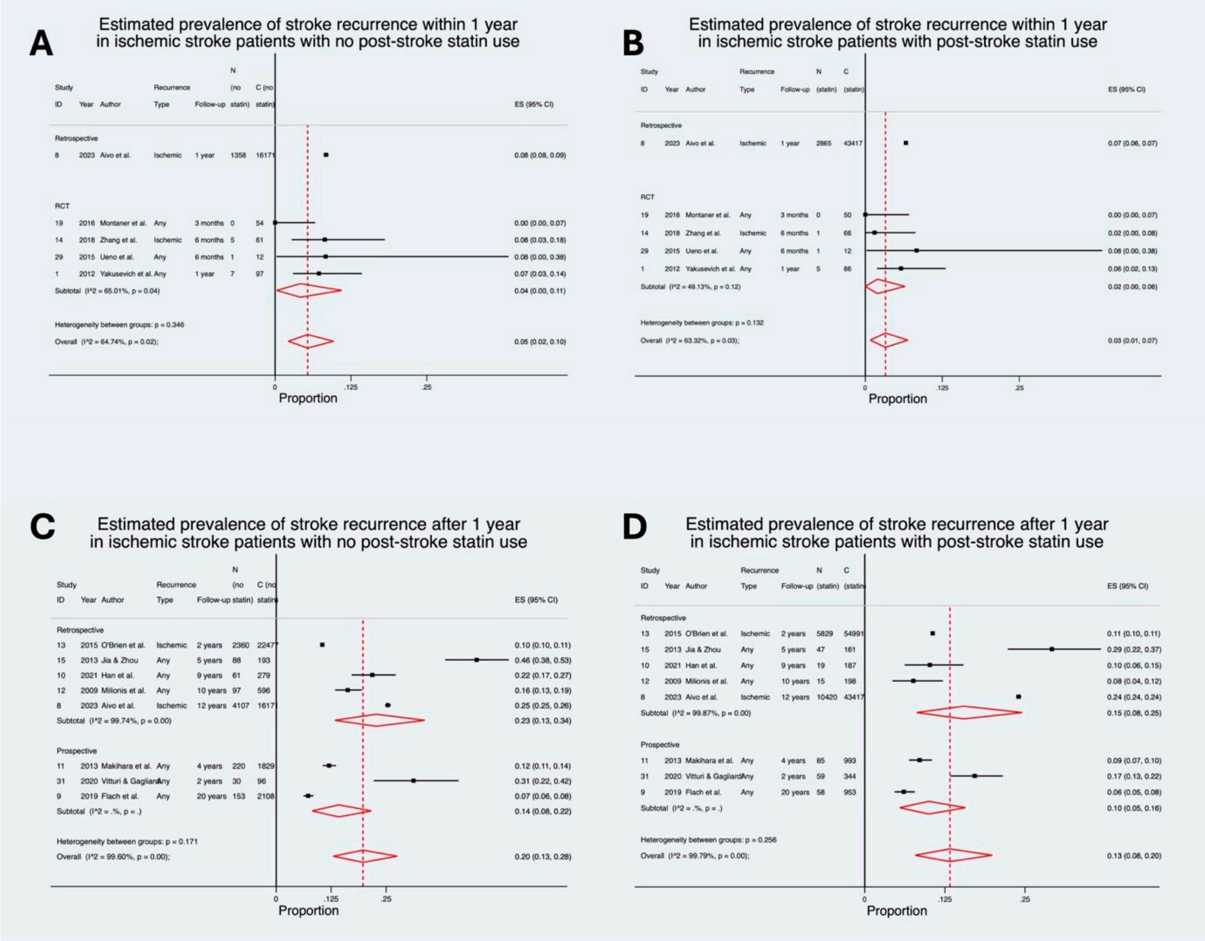

Figure S4. Estimated prevalence of stroke recurrence within: (A, B) 1 year and; (C, D) after 1 year of ischemic stroke in statin users vs nonusers.

Abbreviations: N, number of patients who had a recurrent stroke; C, total number of patients; ES, effect size; p, *p*-value

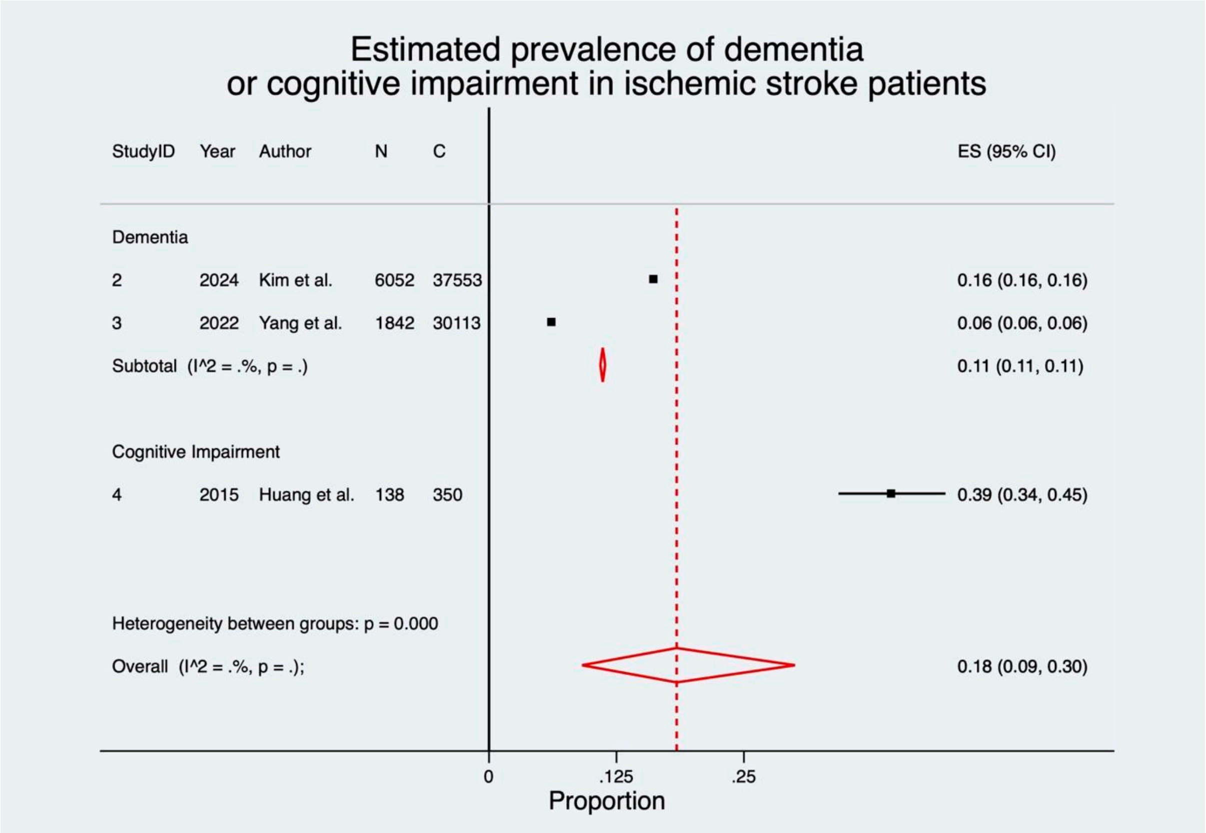

Figure S5. Estimated prevalence of dementia/cognitive impairment after ischemic stroke. Abbreviations: N, number of patients who had dementia or cognitive impairment; C, total number of patients; ES, effect size; p, *p*-value

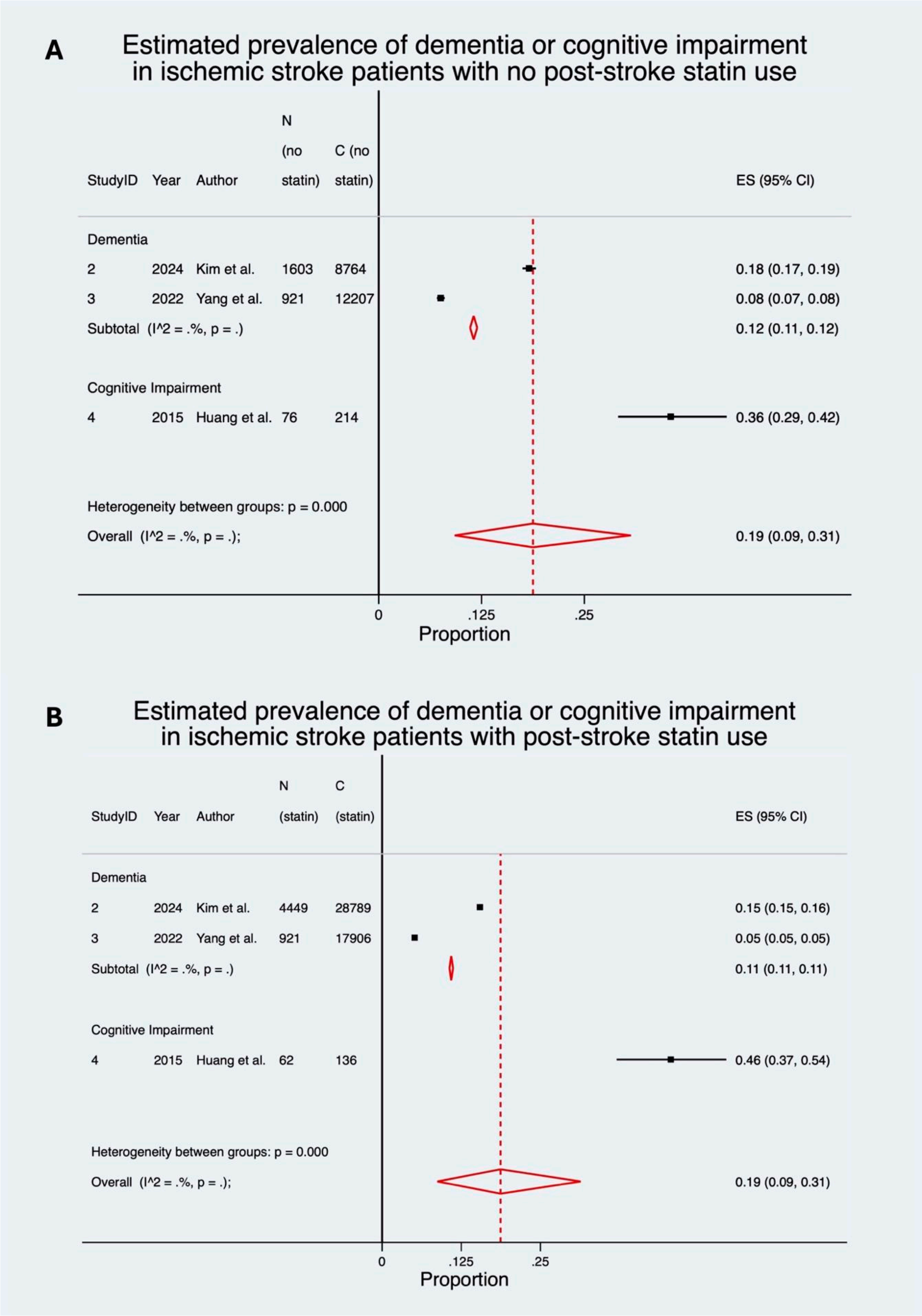

Figure S6. Estimated prevalence of dementia/cognitive impairment after ischemic stroke in: (A) statin users and; (B) nonusers.

Abbreviations: N, number of patients who had dementia or cognitive impairment; C, total number of patients; ES, effect size; p, *p*-value

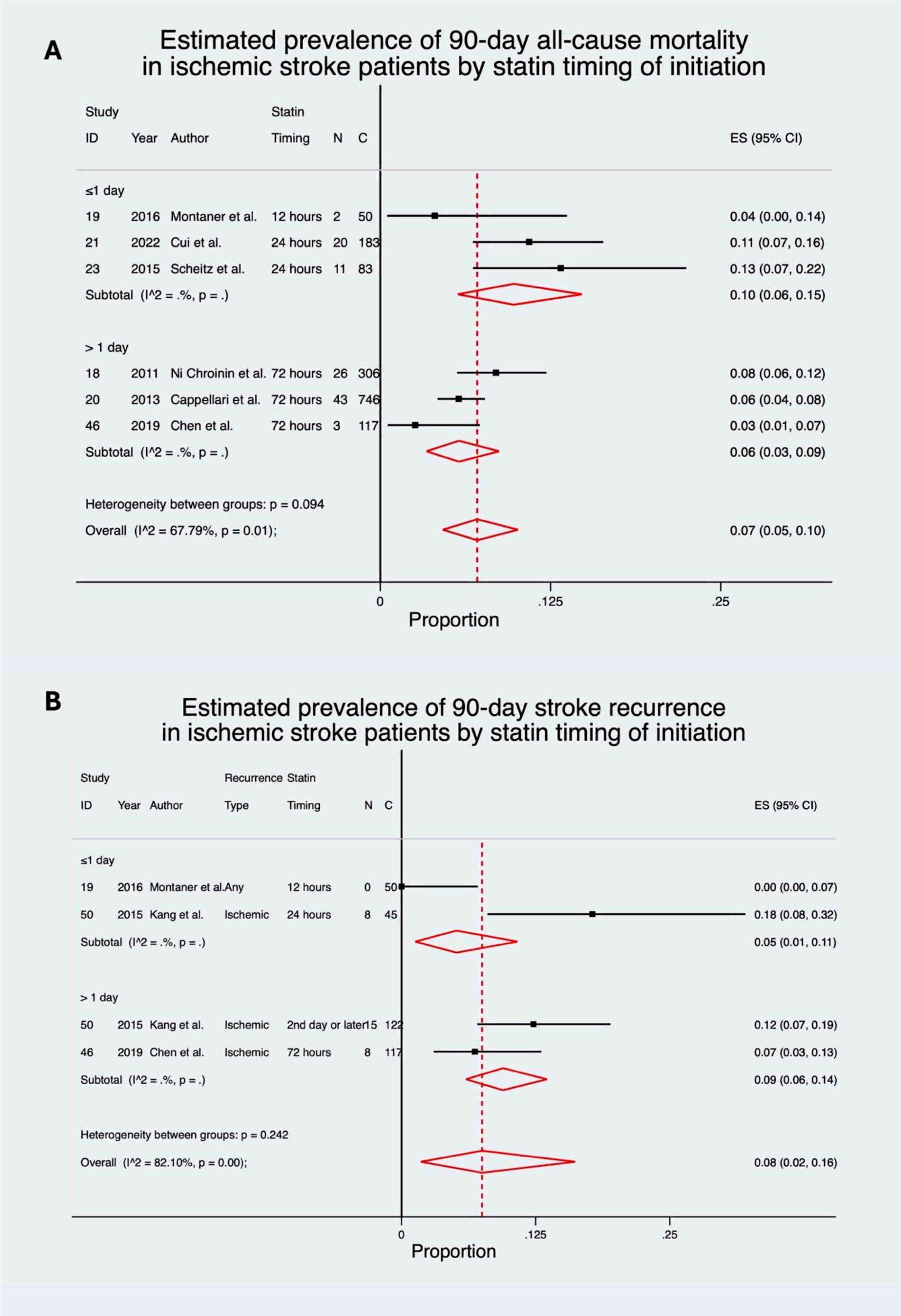

Figure S7. Estimated prevalence of: (A) all-cause mortality and; (B) stroke recurrence with 90-days of ischemic stroke by statin timing of initiation.

Abbreviations: N, number of patients who had died or had recurrent stroke; C, total number of patients; ES, effect size; p, *p*-value

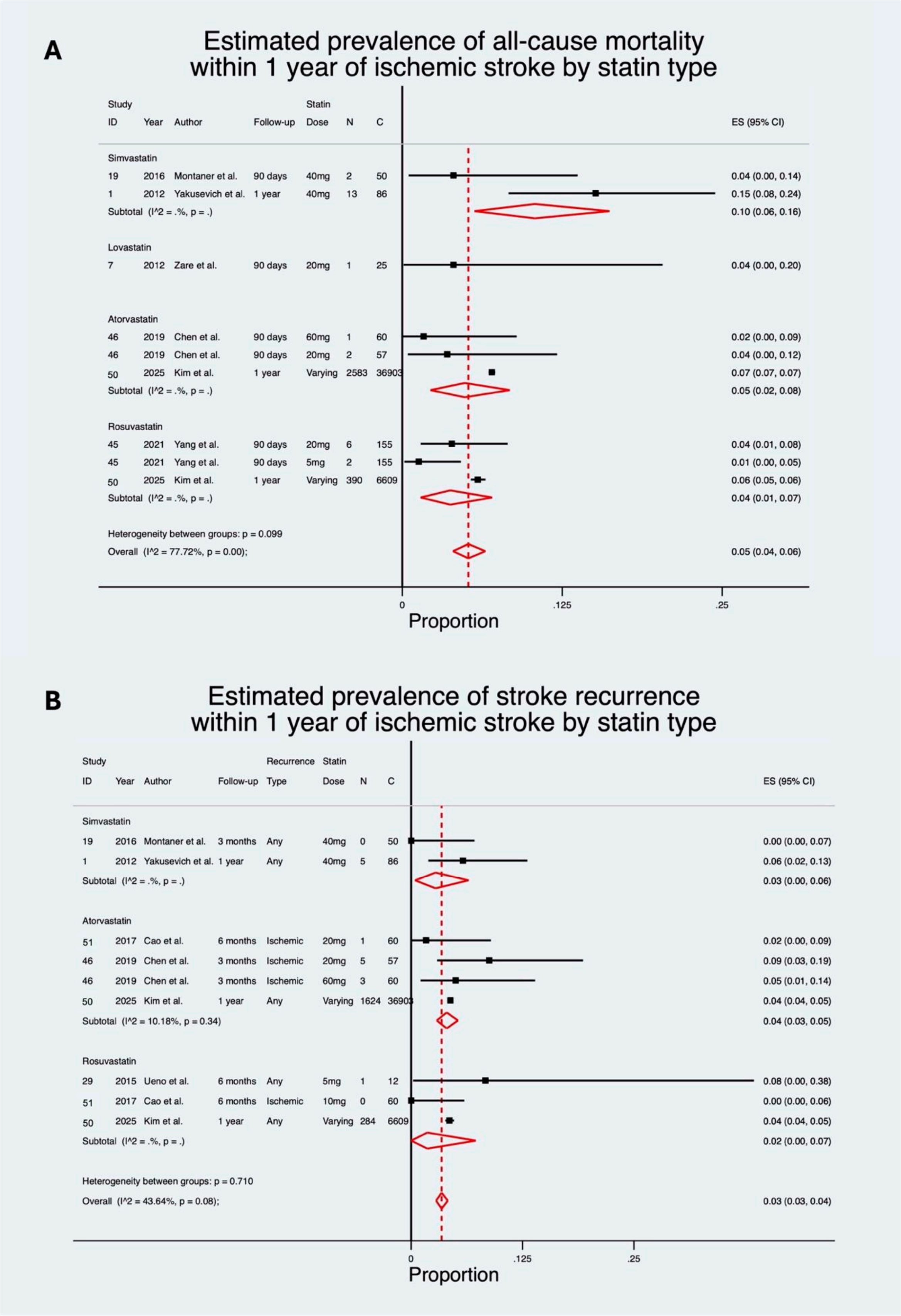

Figure S8. Estimated prevalence of: (A) all-cause mortality and; (B) stroke recurrence within 1 year of ischemic stroke by statin type.

Abbreviations: N, number of patients who had died or had recurrent stroke; C, total number of patients; ES, effect size; p, *p*-value

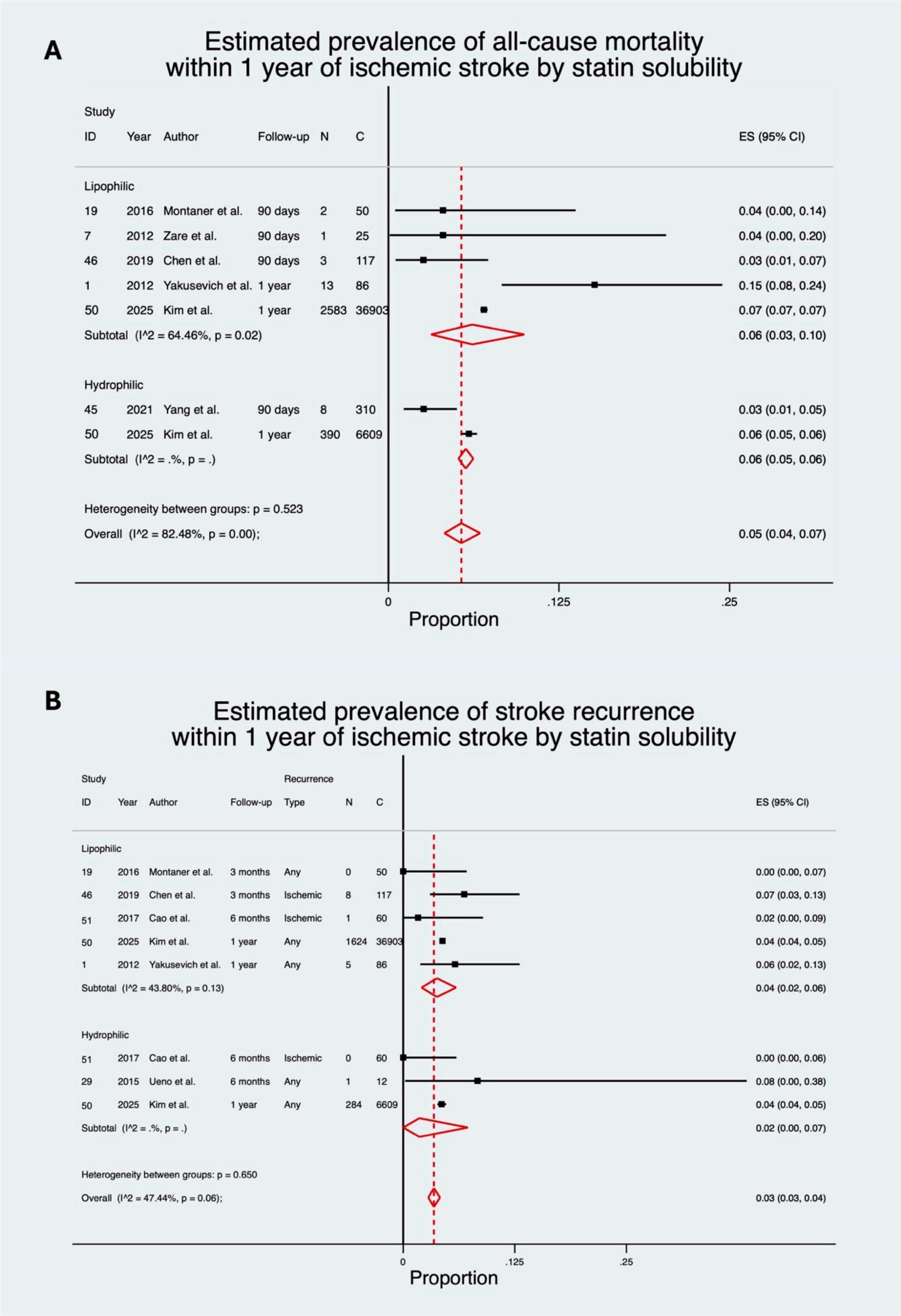

Figure S9. Estimated prevalence of: (A) all-cause mortality and; (B) stroke recurrence within 1 year of ischemic stroke by statin solubility.

Abbreviations: N, number of patients who had died or had recurrent stroke; C, total number of patients; ES, effect size; p, *p*-value

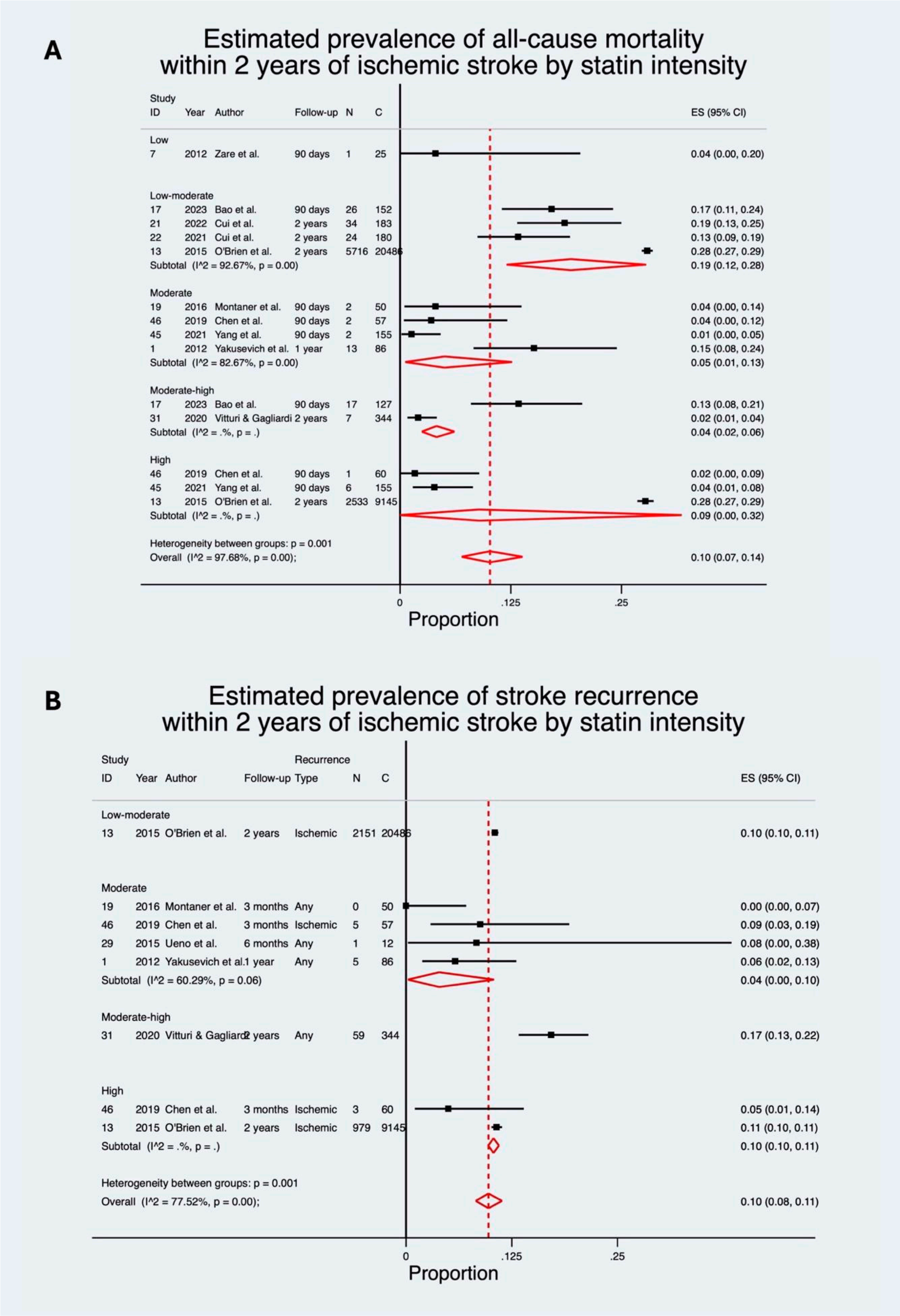

Figure S10. Estimated prevalence of: (A) all-cause mortality and; (B) stroke recurrence within 2 years of ischemic stroke by statin intensity.
Abbreviations: N, number of patients who had died or had recurrent stroke; C, total number of patients; ES, effect size; p, *p*-value

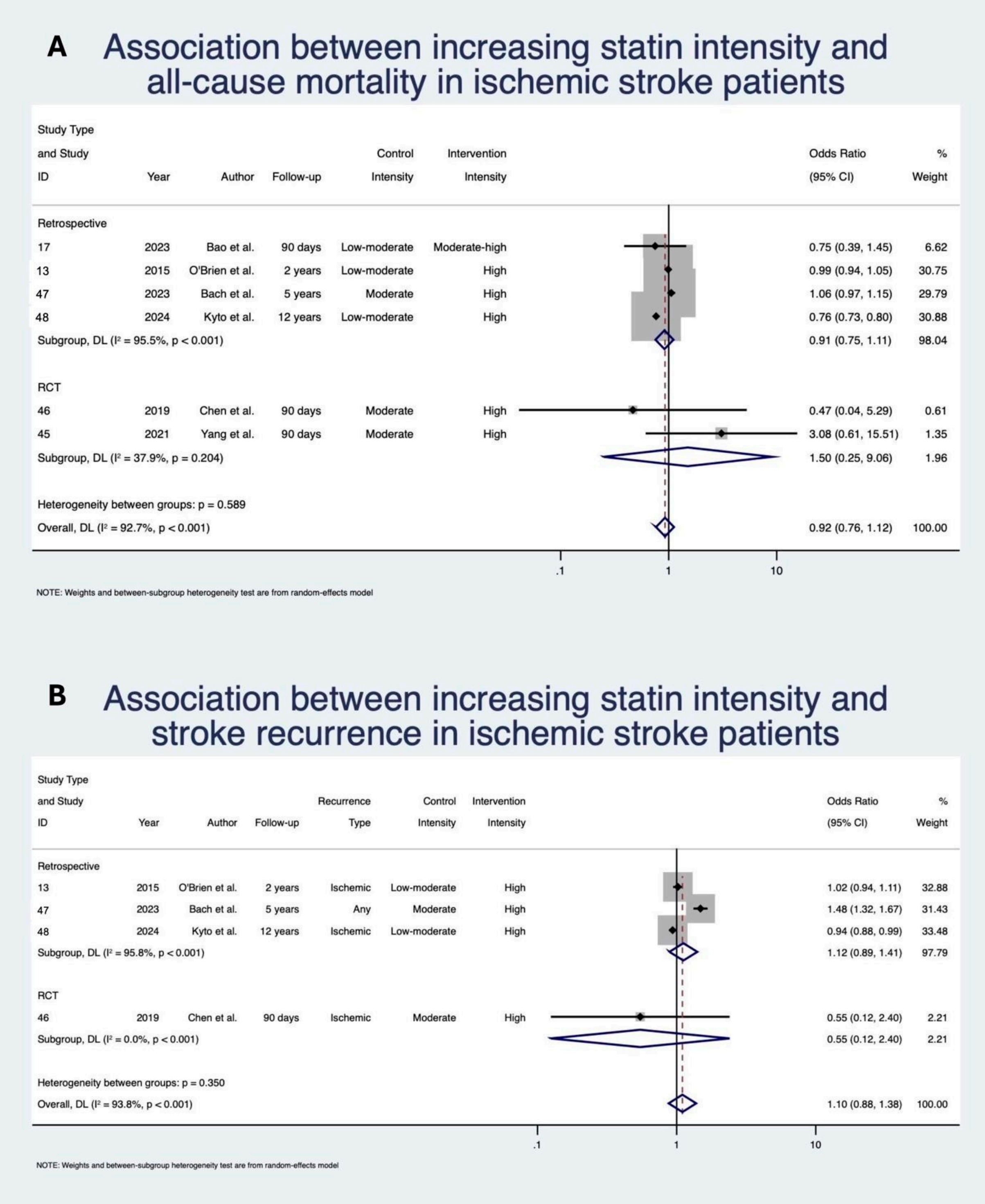

Figure S11. Association between increasing statin intensity and: (A) all-cause mortality and; (B) stroke recurrence after ischemic stroke.
Abbreviations: CI, confidence interval; RCT, randomized controlled trial; DL, DerSimonian and Laird; p, *p*-value

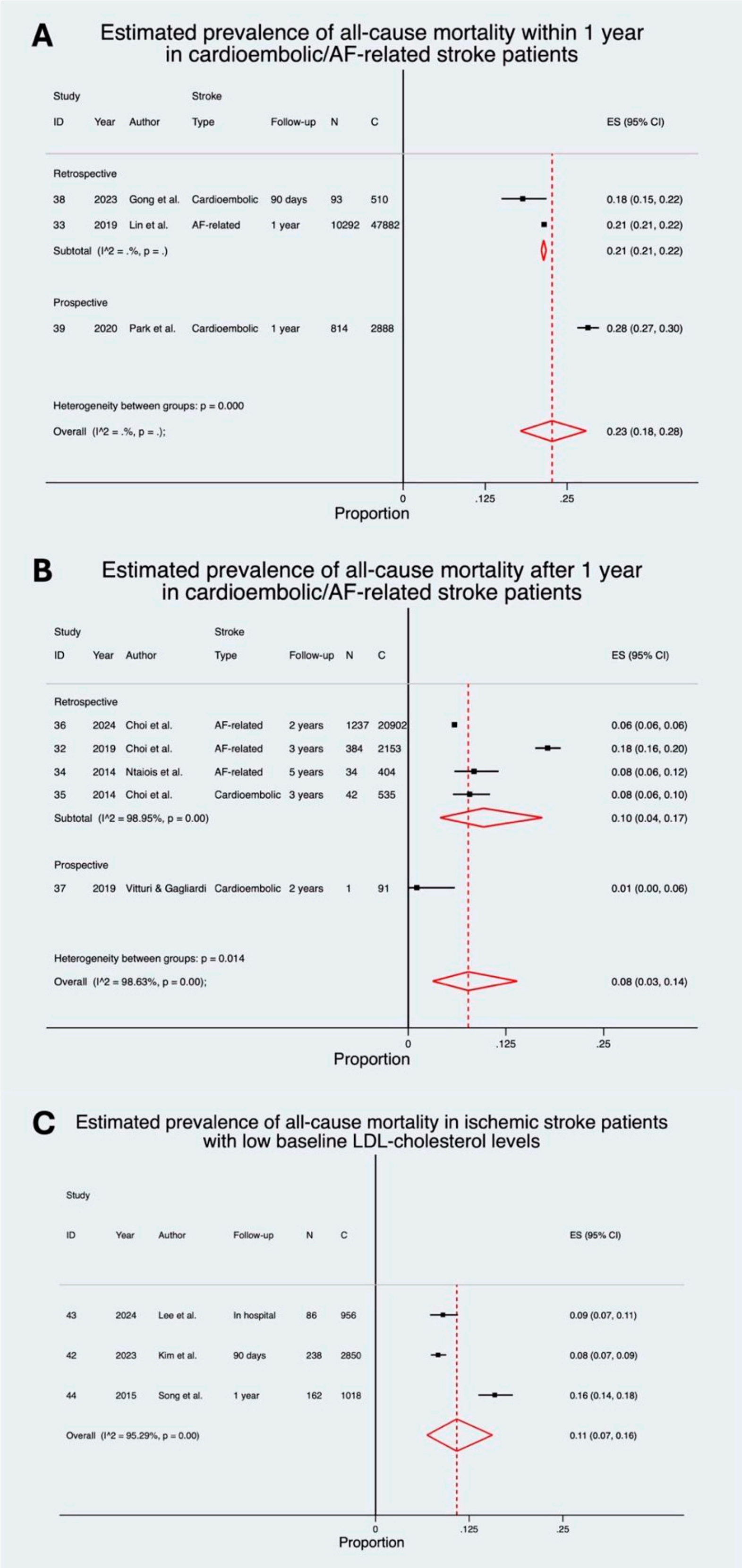

Figure S12. Estimated prevalence of all-cause mortality: (A) within and; (B) after 1 year in cardioembolic/atrial fibrillation stroke patients and; (C) patients with low baseline low-density lipoprotein cholesterol.

Abbreviations: N, number of patients who died; C, total number of patients; ES, effect size; p, *p*-value

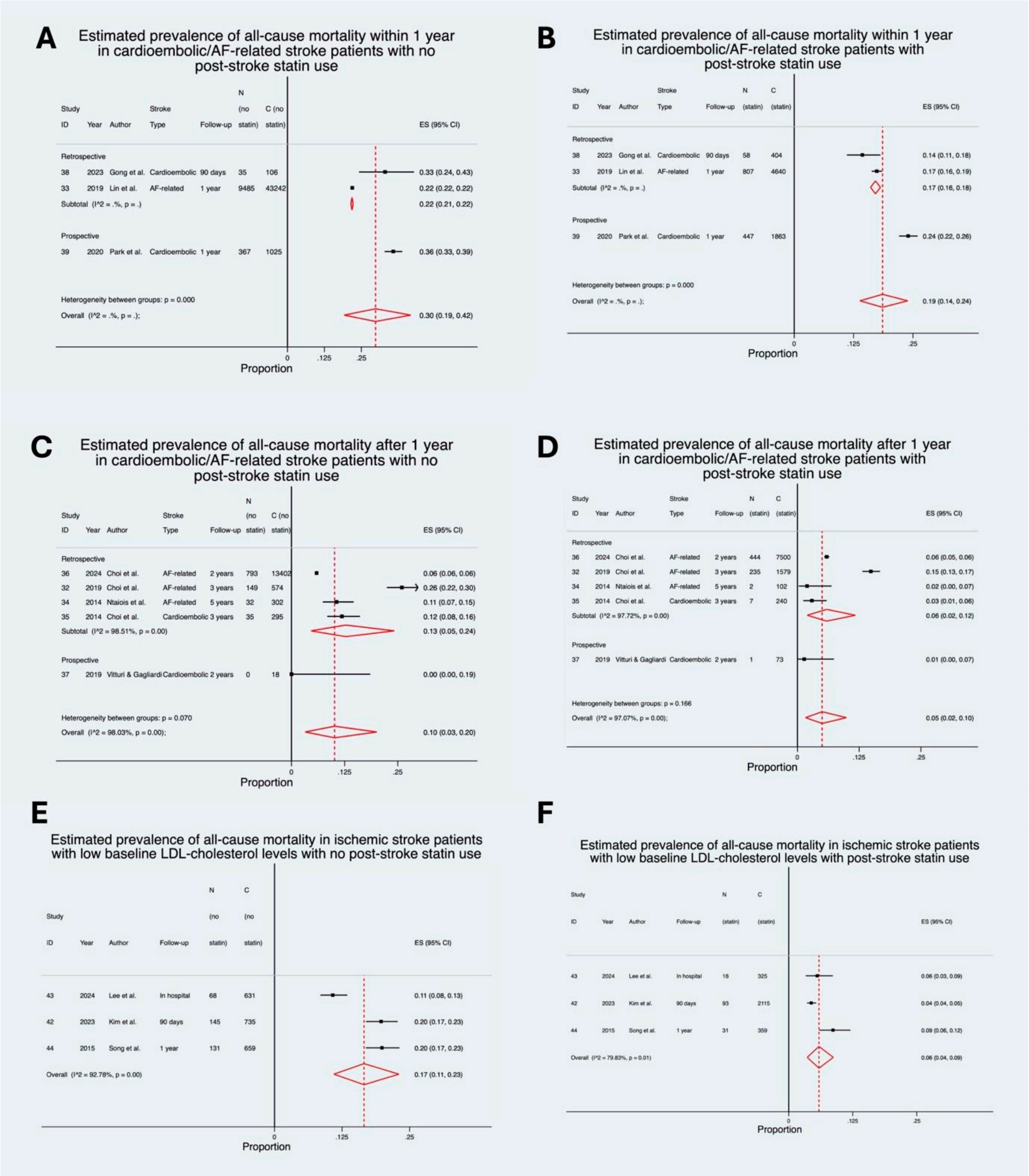

Figure S13. Estimated prevalence of all-cause mortality: (A, B) within and; (C, D) after 1 year in cardioembolic/atrial fibrillation stroke patients and; (E, F) patients with low baseline low-density lipoprotein cholesterol in statin users vs nonusers.

Abbreviations: N, number of patients who died; C, total number of patients; ES, effect size; p, *p*-value

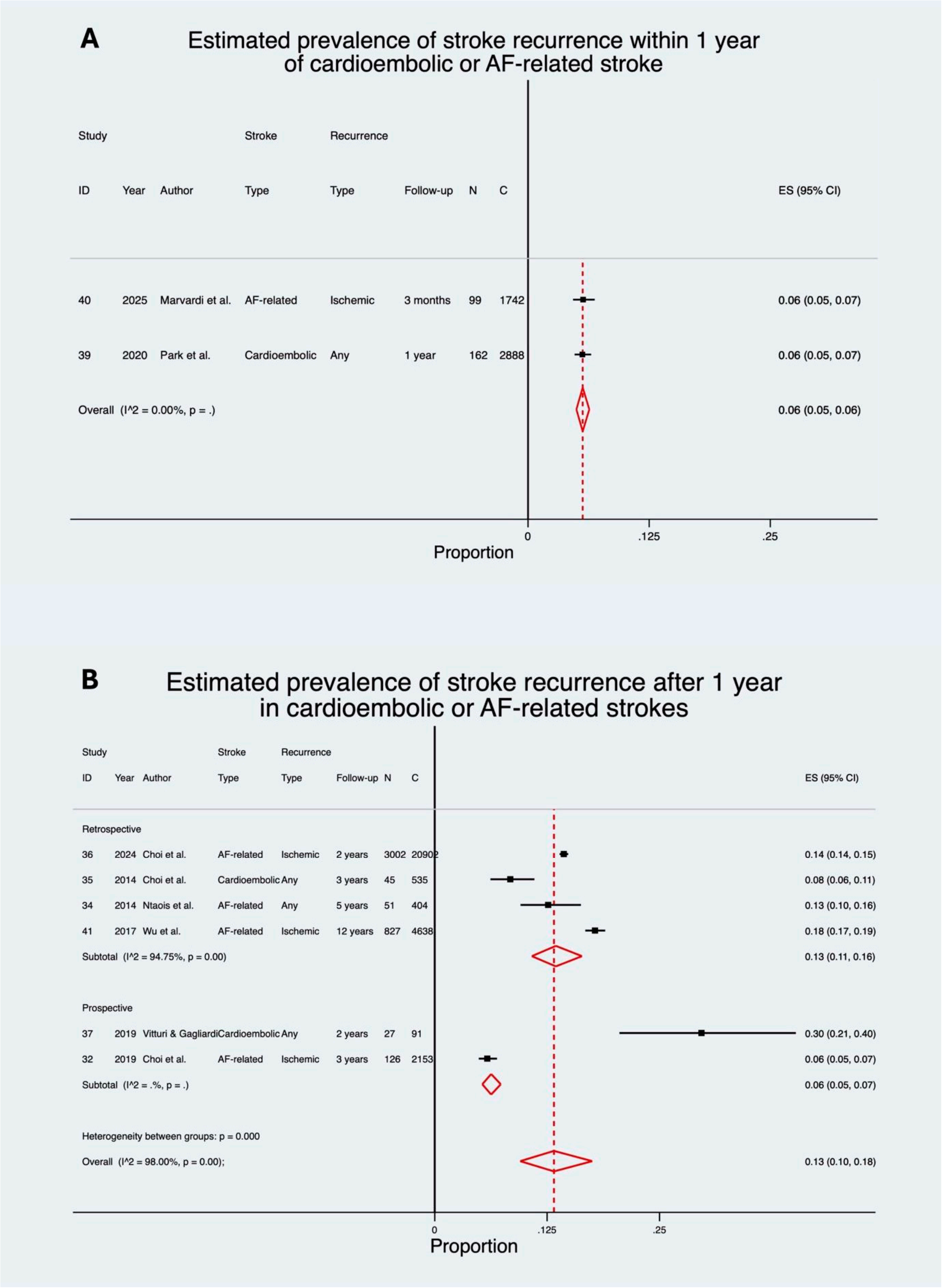

Figure S14. Estimated prevalence of stroke recurrence: (A) within and (B) after 1 year in cardioembolic/atrial fibrillation stroke patients.

Abbreviations: N, number of patients who had a recurrent stroke; C, total number of patients; ES, effect size; p, *p*-value

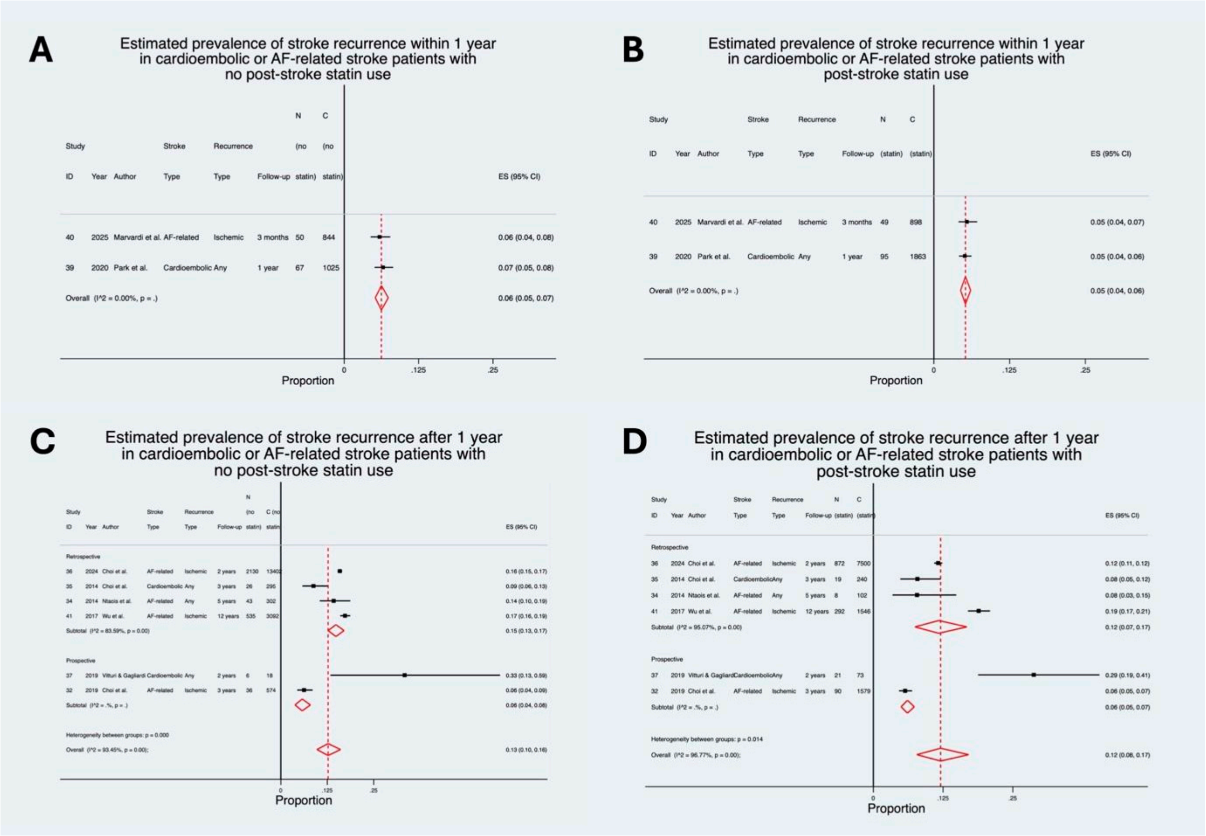

Figure S15. Estimated prevalence of stroke recurrence: (A, B) within and; (C, D) after 1 year in cardioembolic/atrial fibrillation stroke patients in statin users vs nonusers.
Abbreviations: N, number of patients who had a recurrent stroke; C, total number of patients; ES, effect size; p, p-value

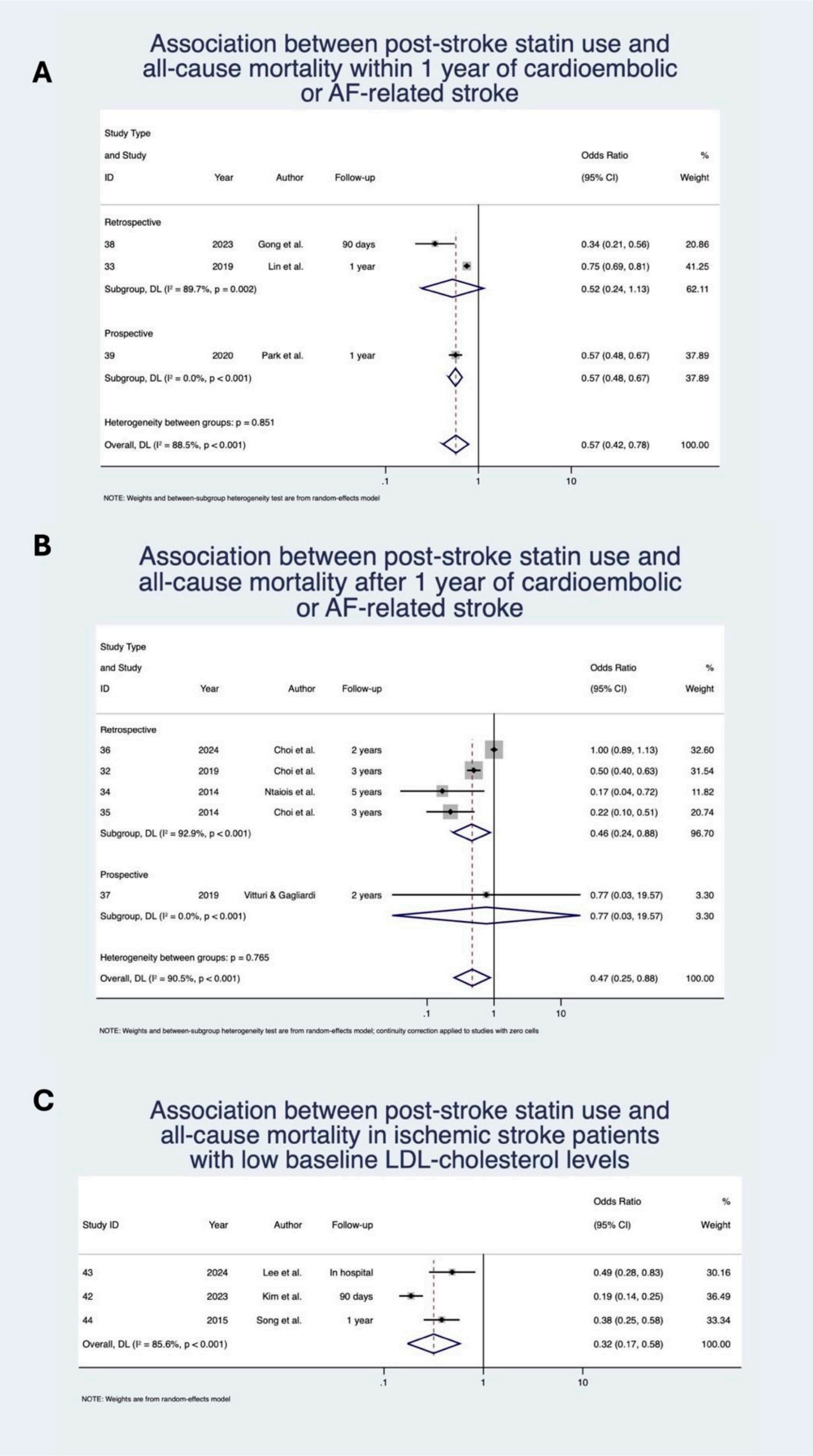

Figure S16. Association between statin use and all-cause mortality: (A) within and; (B) after 1 year in cardioembolic and atrial-fibrillation related stroke patients and; (C) patients with low baseline low-density lipoprotein cholesterol.

Abbreviations: CI, confidence interval; DL, DerSimonian and Laird; p, p-value

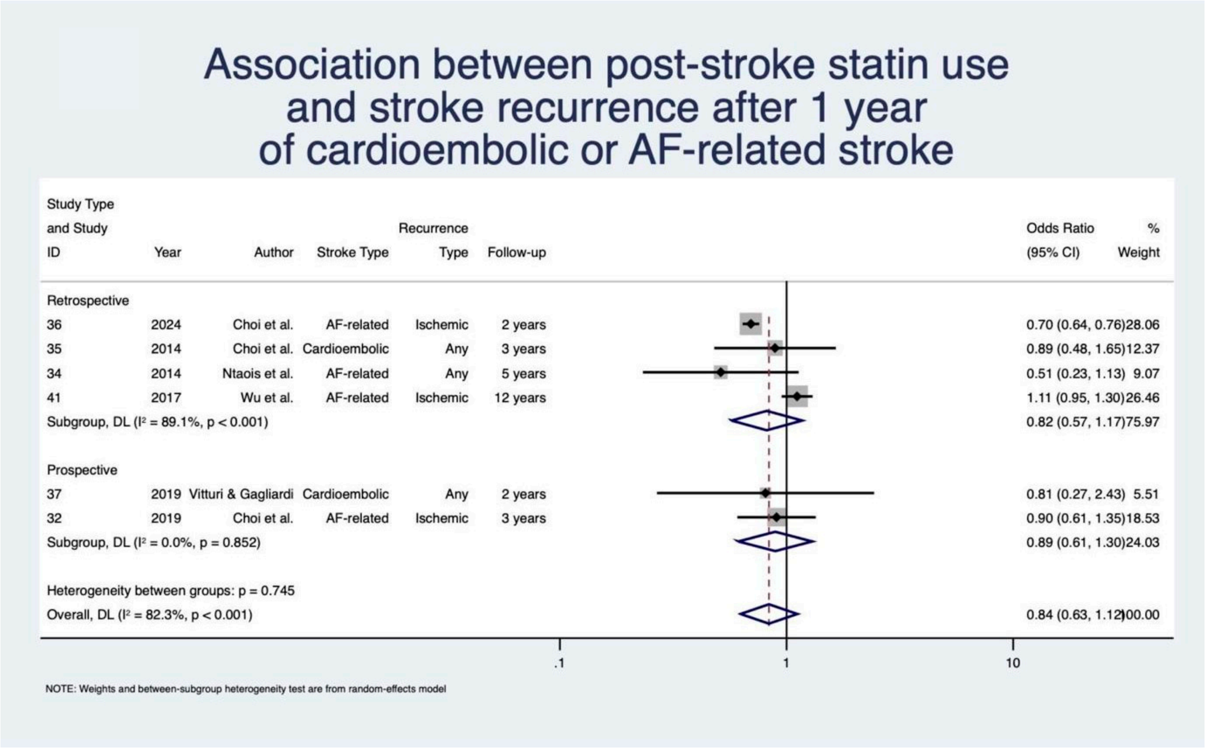

Figure S17. Association between statin use and stroke recurrenceafter 1 year in cardioembolic and atrial-fibrillation related stroke patients.

Abbreviations: CI, confidence interval; DL, DerSimonian and Laird; AF, atrial fibrillation; p, *p*-value

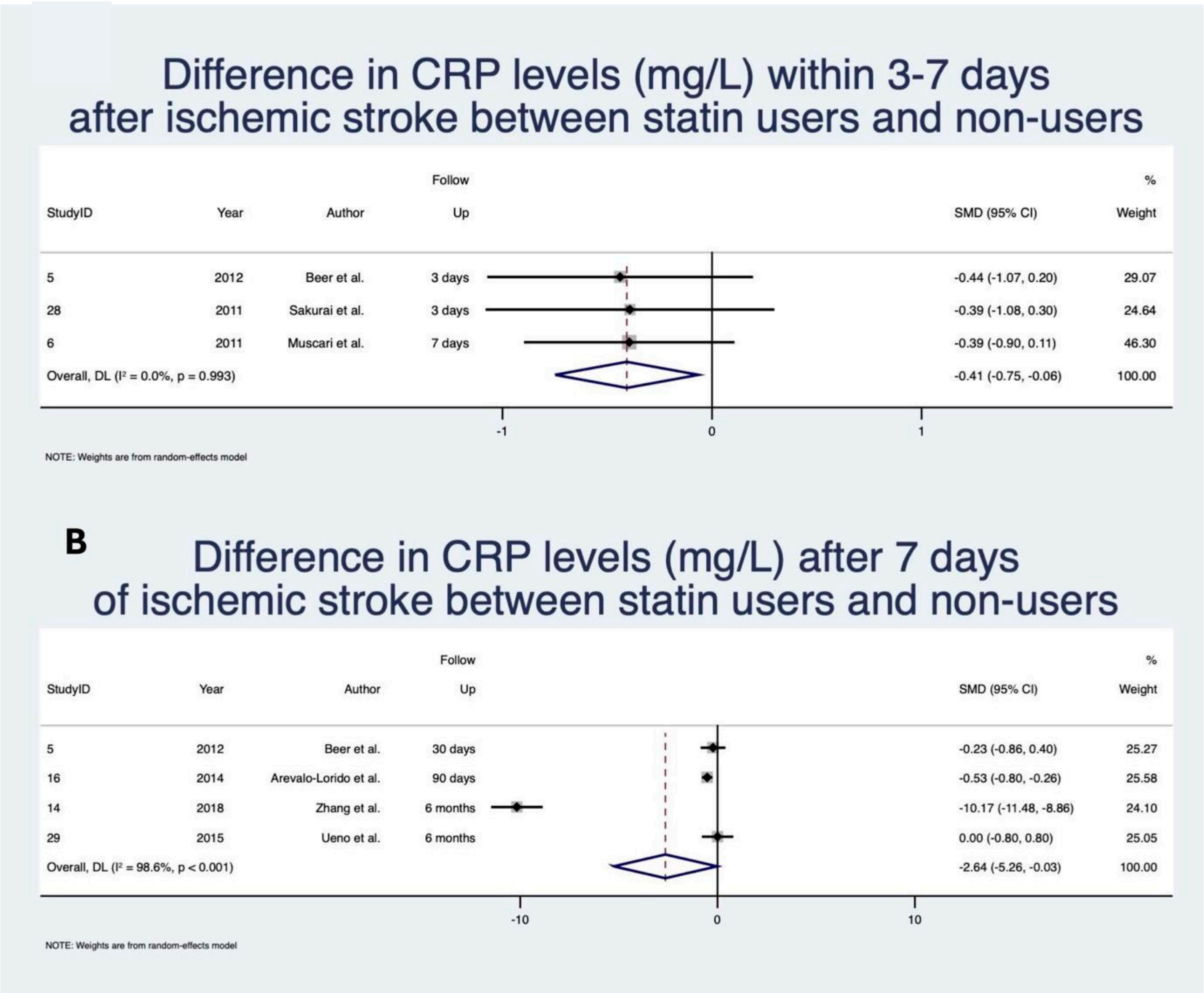

Figure S18. Difference in CRP levels (mg/L) after 7 days of ischemic stroke between statin users and nonusers.

Abbreviations: CRP; C-reactive protein; CI, confidence interval; SMD, standardized mean difference; DL, DerSimonian and Laird

Figure S19. Graphs of Sensitivity Analyses

Studies assessing the association between post-stroke statin use and dementia/ cognitive Impairment

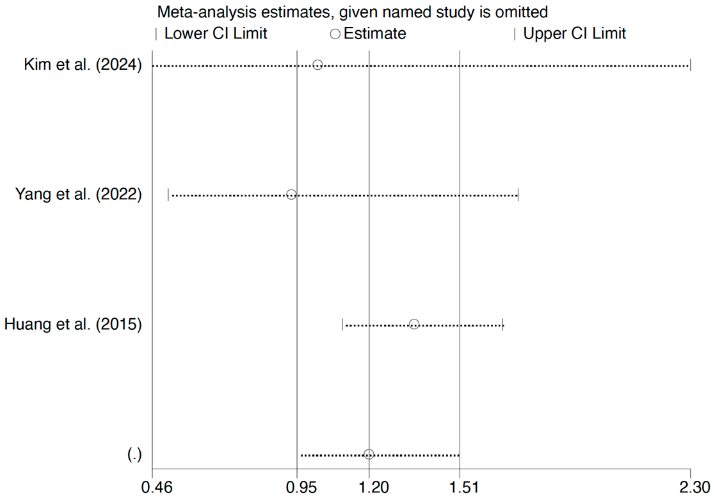

Studies assessing the association between post-stroke statin use and mortality within 3 months of ischemic stroke

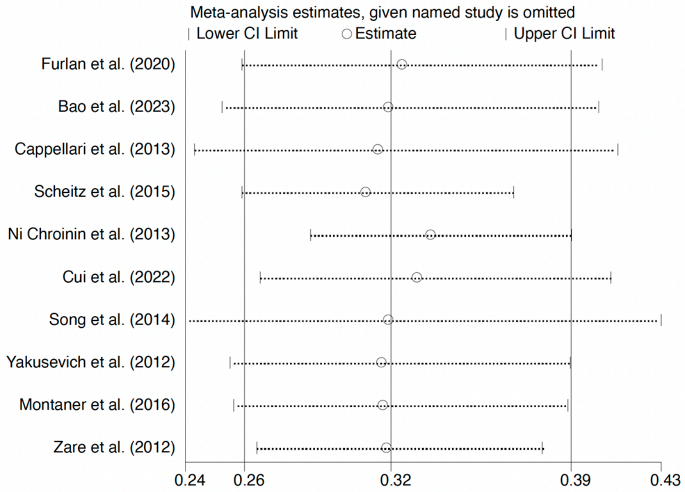

Studies assessing the association between post-stroke statin use and mortality within 1 year of ischemic stroke

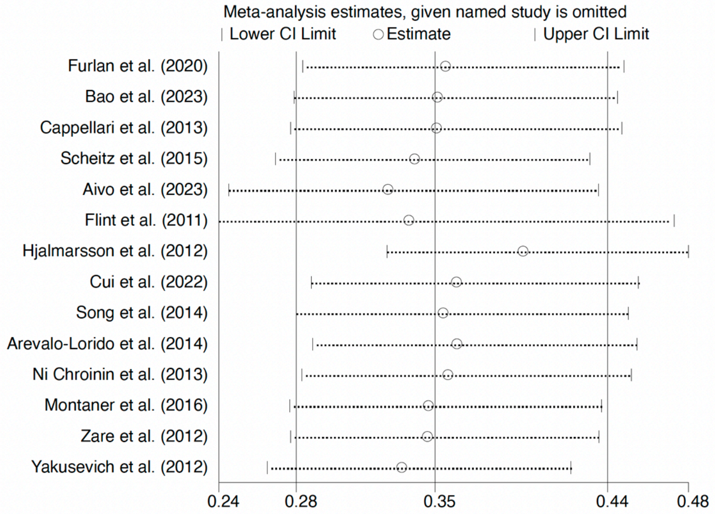

Studies assessing the association between post-stroke statin use and mortality after 1 year of ischemic stroke	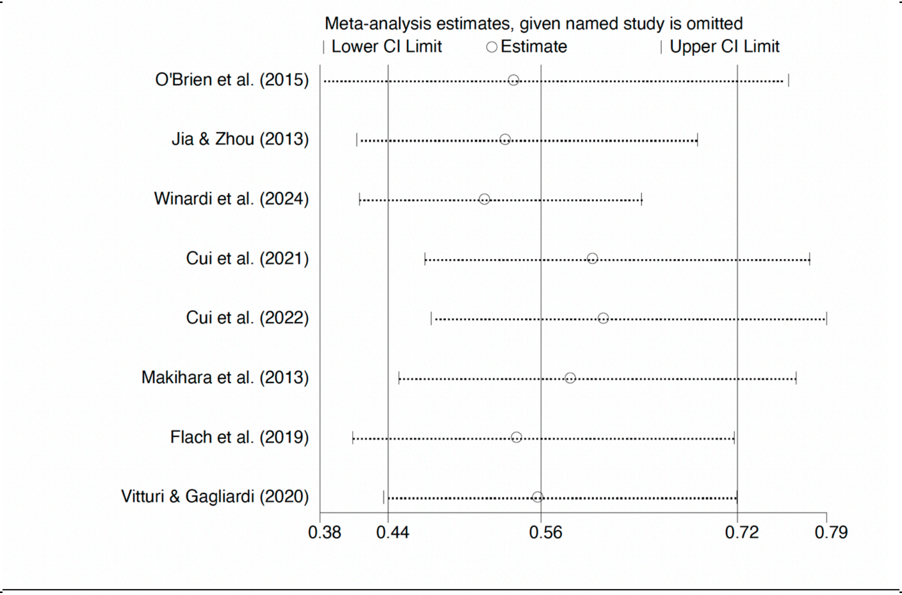
Studies assessing the association between post-stroke statin use and mortality within 1 year of cardioembolic or AF-related stroke	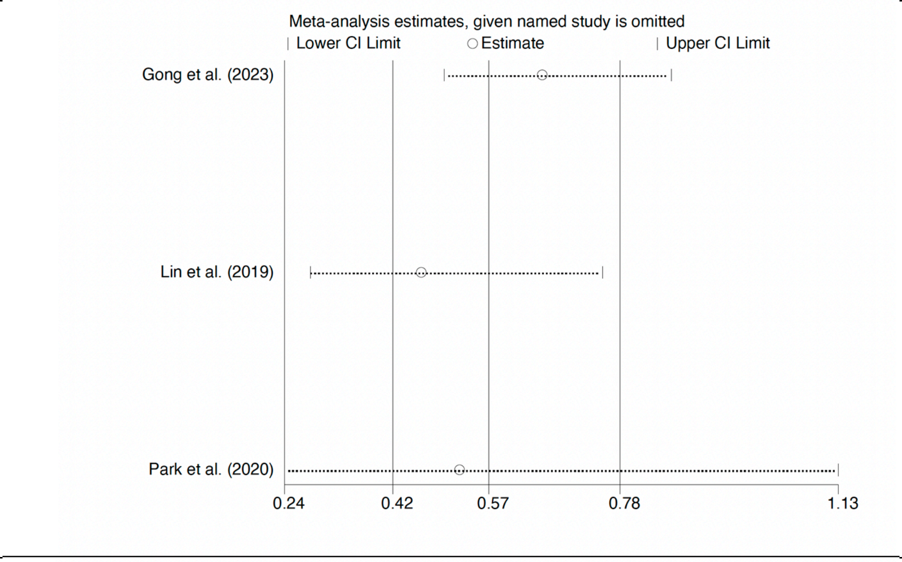
Studies assessing the association between post-stroke statin use and mortality after 1 year of cardioembolic or AF-related stroke	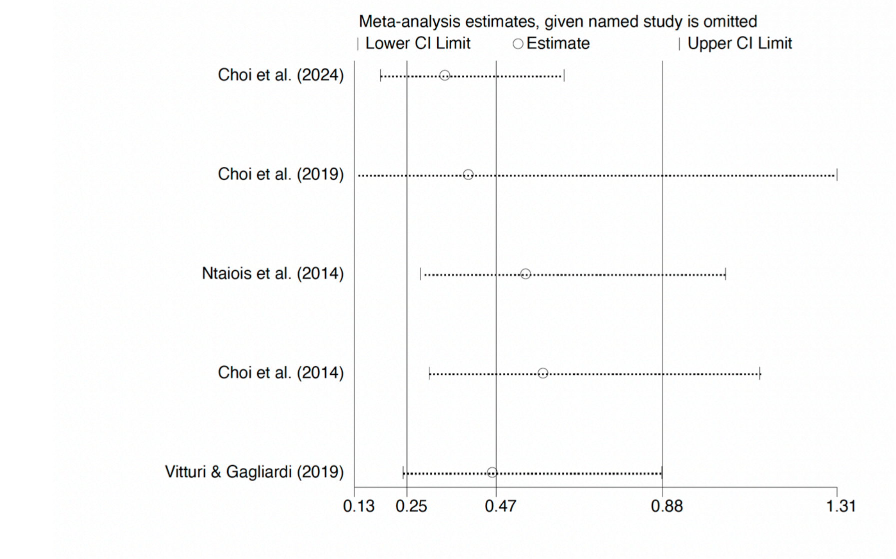

Studies assessing the association between post-stroke statin use and mortality within patients with low baseline LDL-cholesterol levels	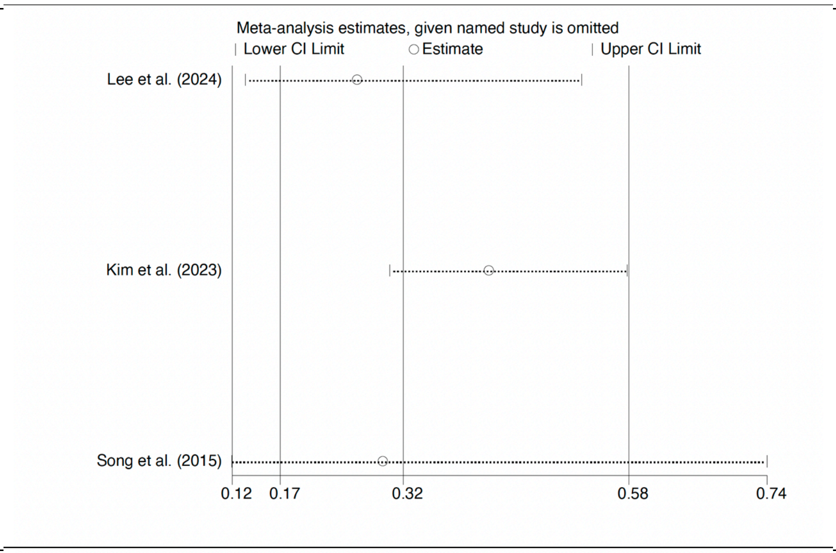
Studies assessing the association between post-stroke statin use and stroke recurrence within 1 year of ischemic stroke	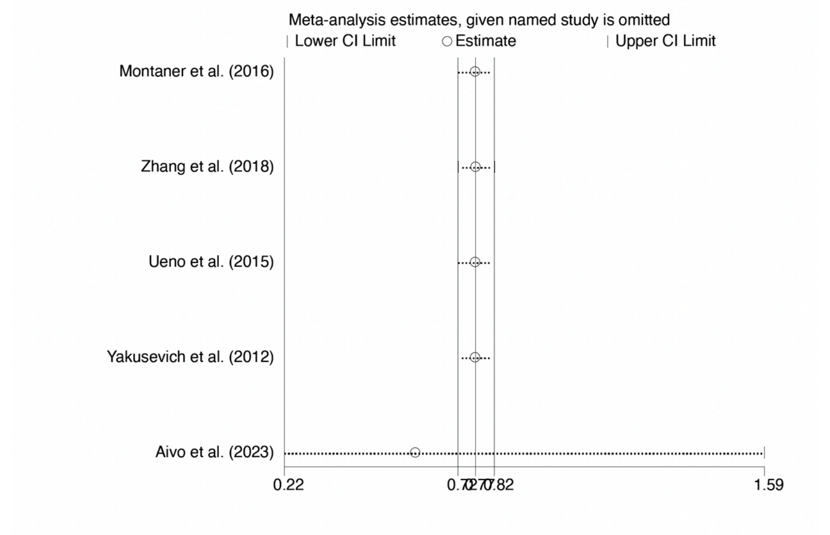
Studies assessing the association between post-stroke statin use and stroke recurrence after 1 year of ischemic stroke	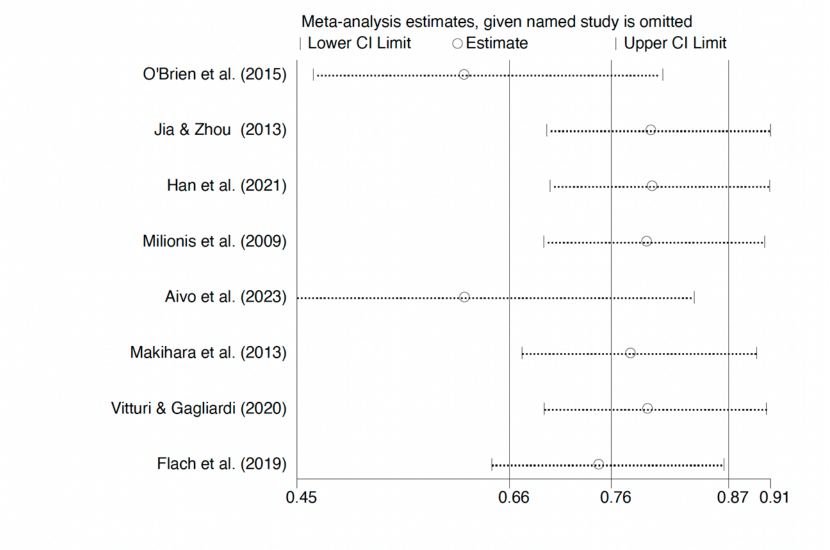

Studies assessing the association between post-stroke statin use and stroke recurrence within 1 year of cardioembolic or AF-related stroke	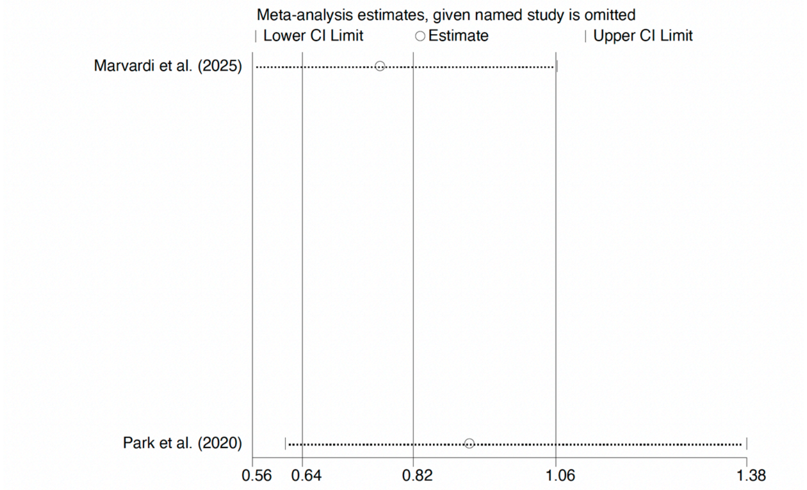
Studies assessing the association between post-stroke statin use and stroke recurrence after 1 year of cardioembolic or AF-related stroke	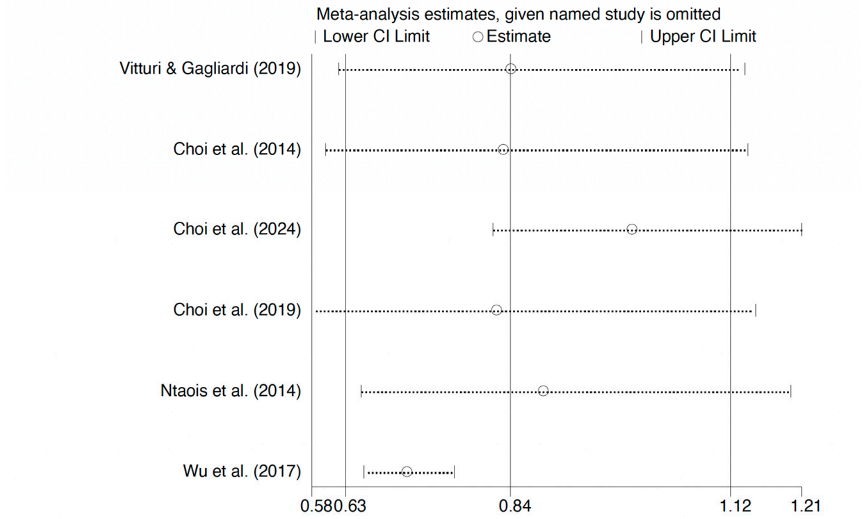
Studies assessing association between increasing statin intensity and all-cause mortality	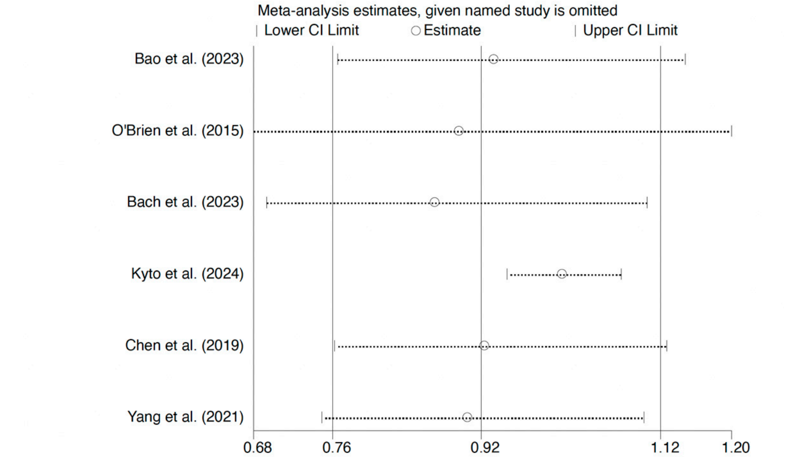

Studies assessing association between increasing statin intensity and stroke recurrence	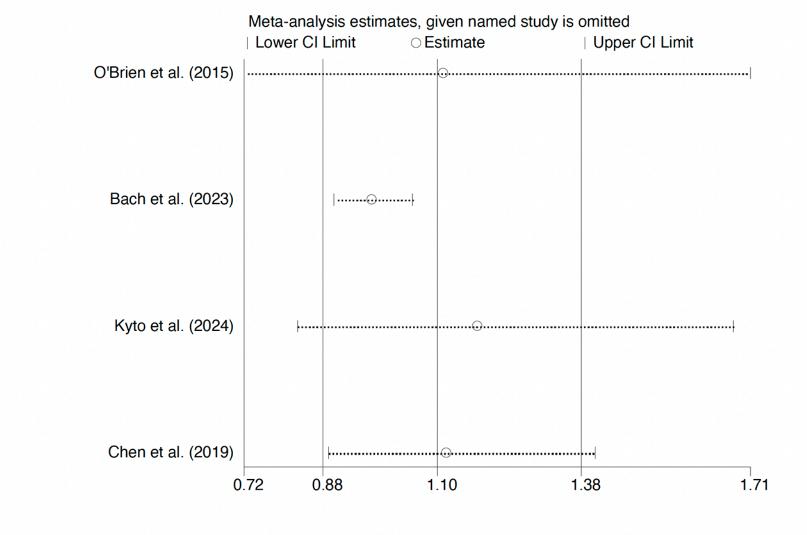

Abbreviations: CI, confidence interval; AF, atrial fibrillation

Figure S20. Graphs of Egger's Regression Test

Studies assessing the association between post-stroke statin use and dementia/ cognitive Impairment	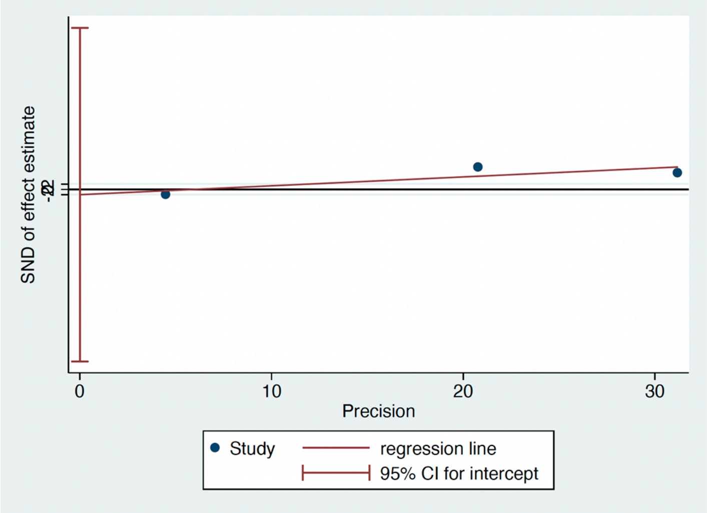
Studies assessing the association between post-stroke statin use and mortality within 3 months of ischemic stroke	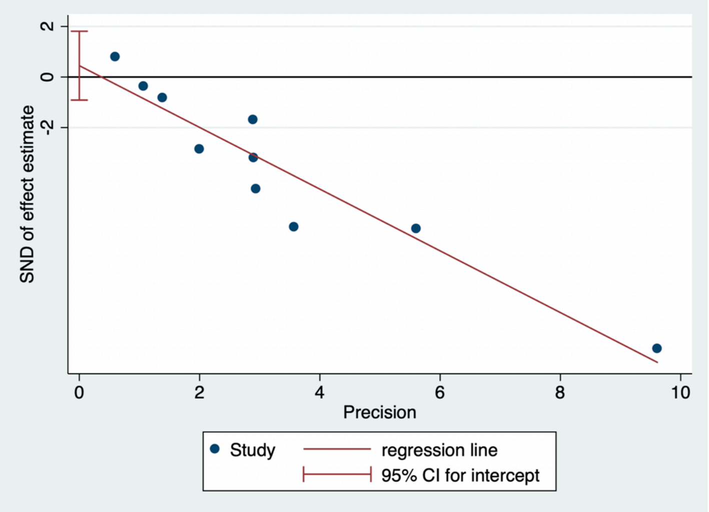
Studies assessing the association between post-stroke statin use and mortality within 1 year of ischemic stroke	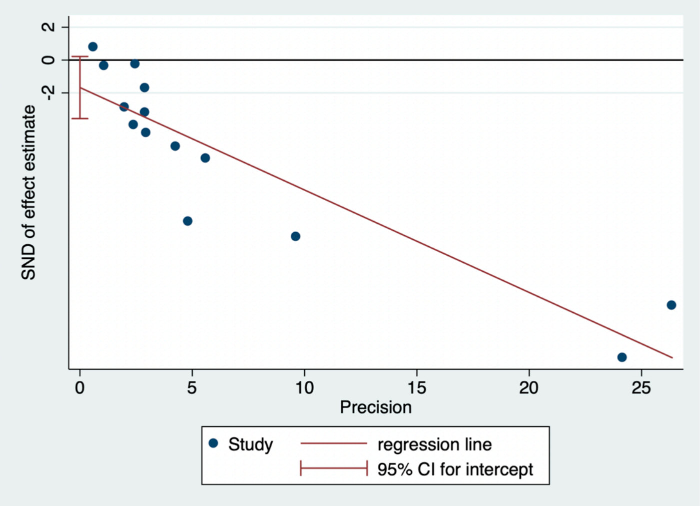

Studies assessing the association between post-stroke statin use and mortality after 1 year of ischemic stroke	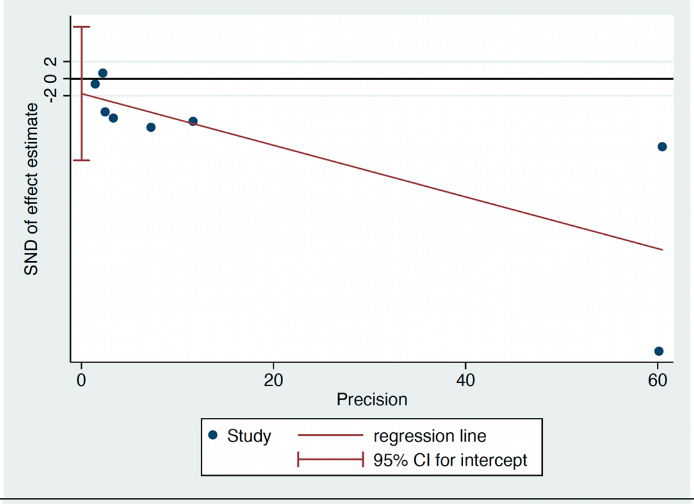
Studies assessing the association between post-stroke statin use and mortality within 1 year of cardioembolic or AF-related stroke	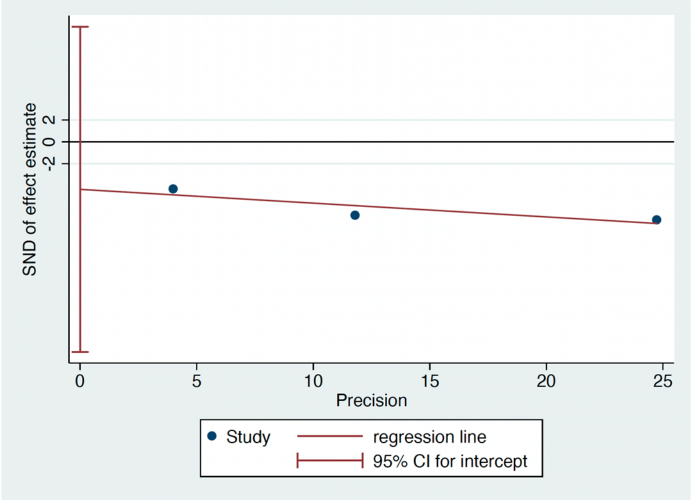
Studies assessing the association between post-stroke statin use and mortality after 1 year of cardioembolic or AF-related stroke	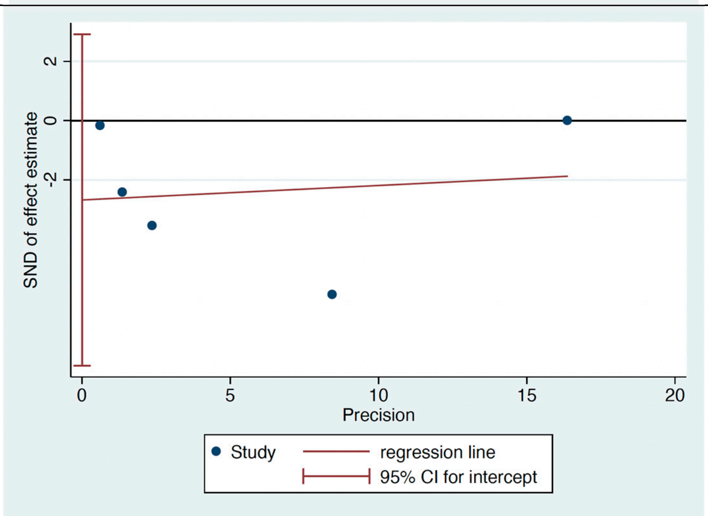

Studies assessing the association between post-stroke statin use and mortality within patients with low baseline LDL-cholesterol levels	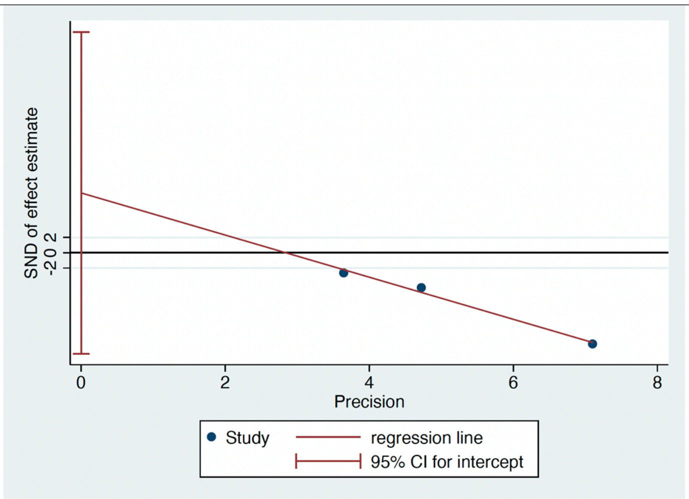
Studies assessing the association between post-stroke statin use and stroke recurrence within 1 year of ischemic stroke	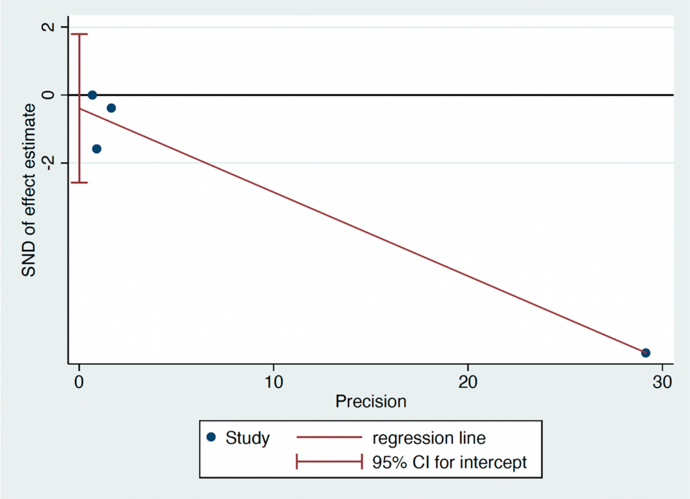
Studies assessing the association between post-stroke statin use and stroke recurrence after 1 year of ischemic stroke	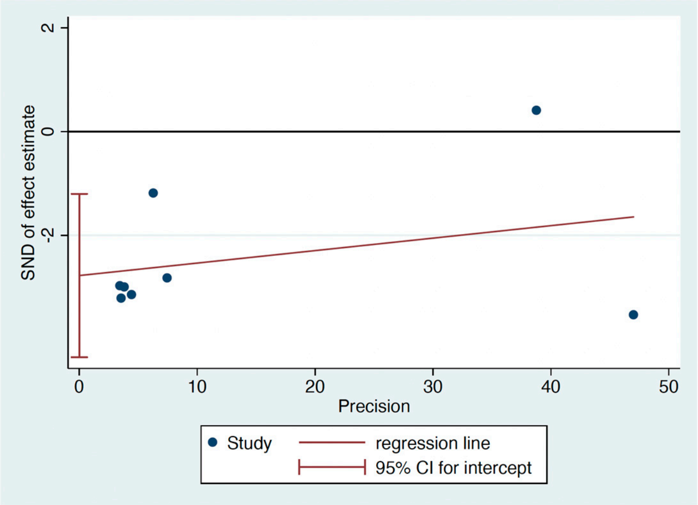

Studies assessing the association between post-stroke statin use and stroke recurrence within 1 year of cardioembolic or AF-related stroke	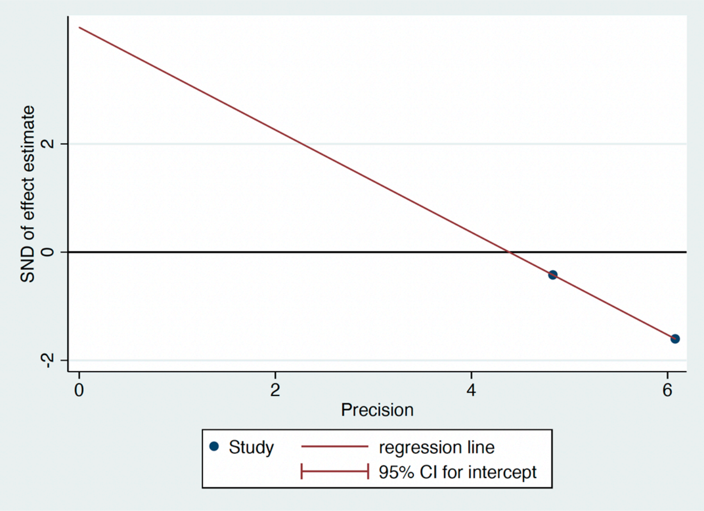
Studies assessing the association between post-stroke statin use and stroke recurrence after 1 year of cardioembolic or AF-related stroke	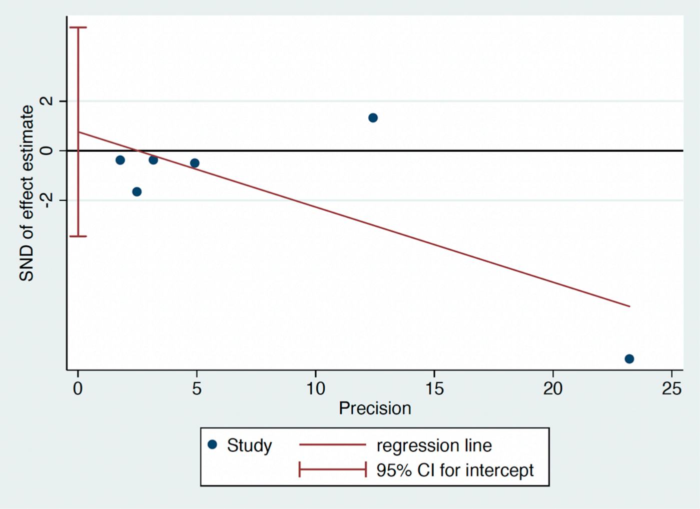
Studies assessing association between increasing statin intensity and all-cause mortality	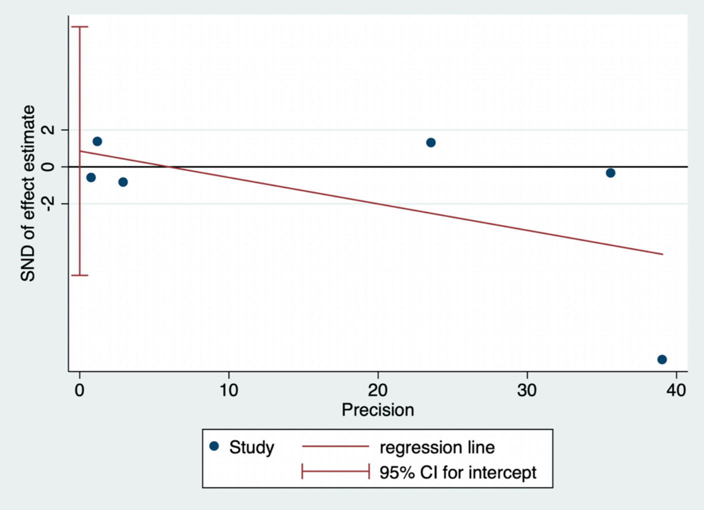

Studies assessing association between increasing statin intensity and stroke recurrence	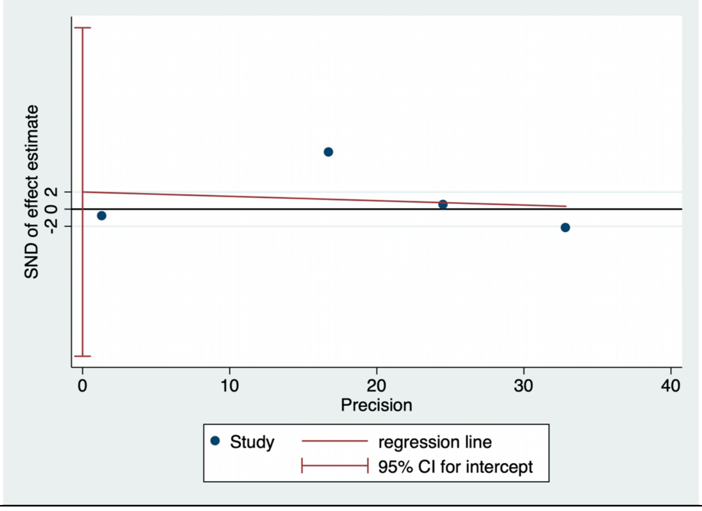

Abbreviations: SND, standard normal deviate; CI, confidence interval; AF, atrial fibrillation

Figure S21. Graphs of Funnel Plots

Studies assessing the association between post-stroke statin use and dementia/ cognitive Impairment	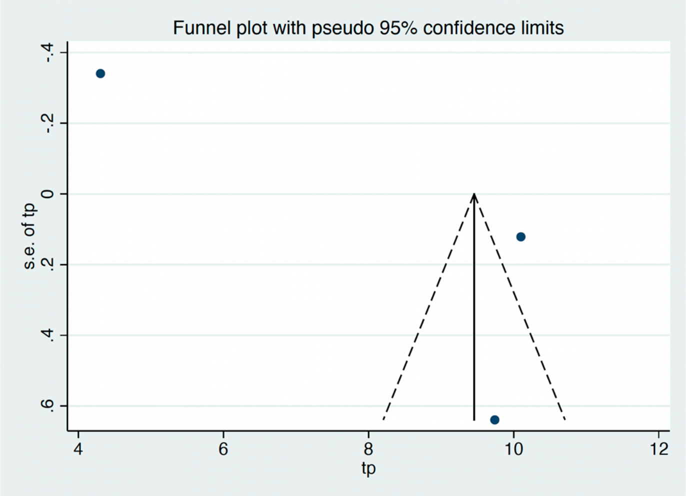
Studies assessing the association between post-stroke statin use and mortality within 3 months of ischemic stroke	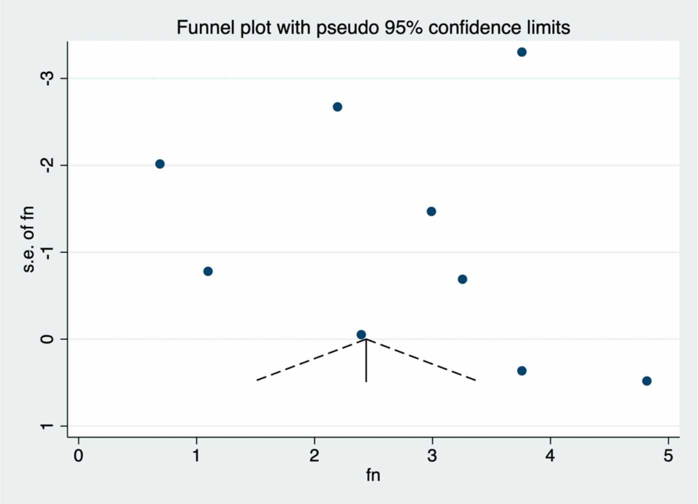
Studies assessing the association between post-stroke statin use and mortality within 1 year of ischemic stroke	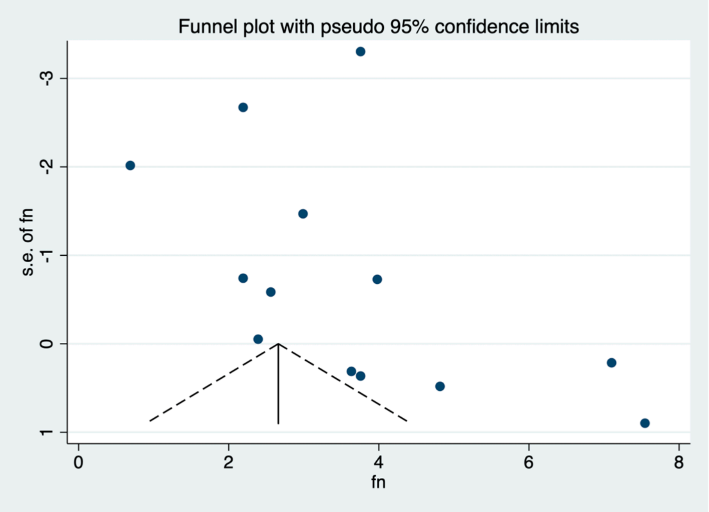

Studies assessing the association between post-stroke statin use and mortality after 1 year of ischemic stroke		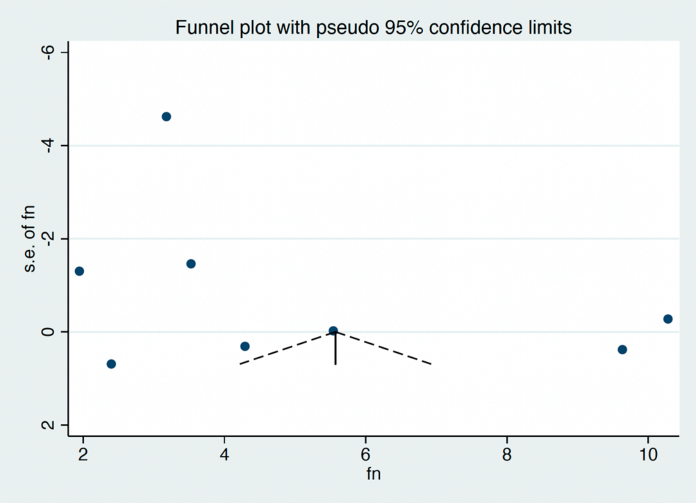
Studies assessing the association between post-stroke statin use and mortality within 1 year of cardioembolic or AF-related stroke		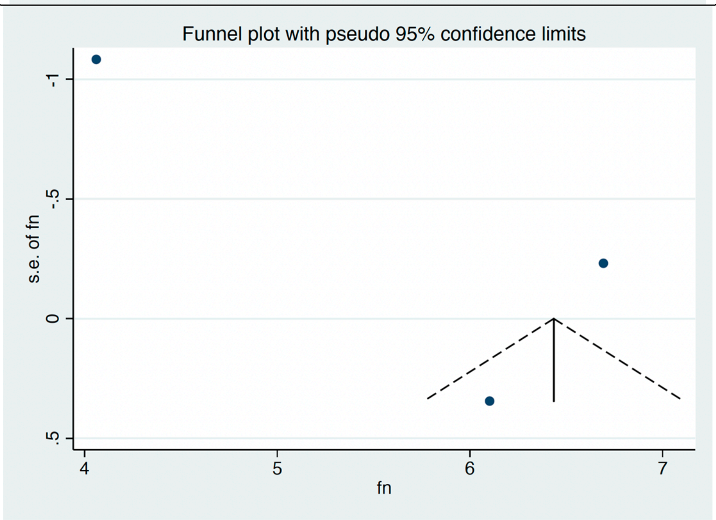
Studies assessing the association between post-stroke statin use and mortality after 1 year of cardioembolic or AF-related stroke		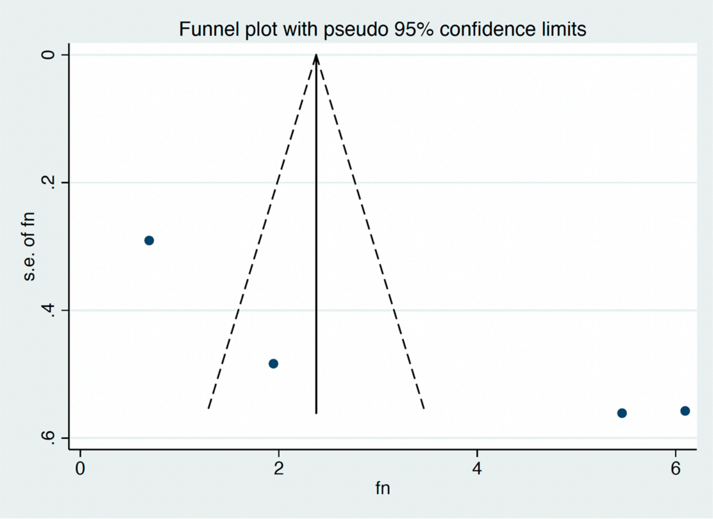

Studies assessing the association between post-stroke statin use and mortality within patients with low baseline LDL-cholesterol levels	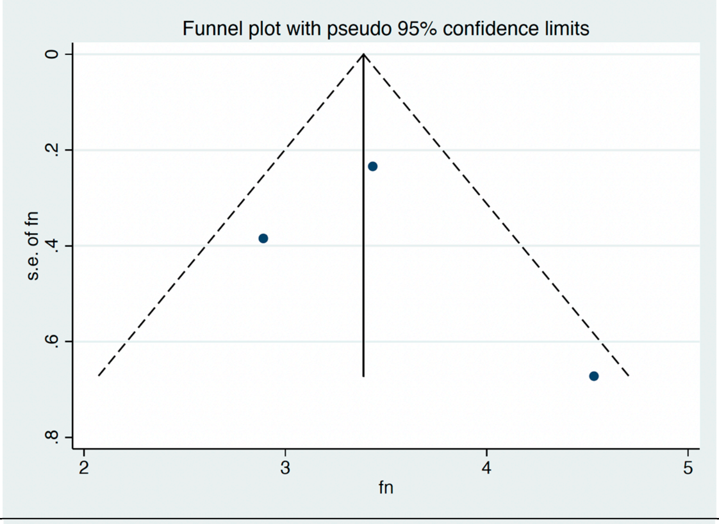
Studies assessing the association between post-stroke statin use and stroke recurrence within 1 year of ischemic stroke	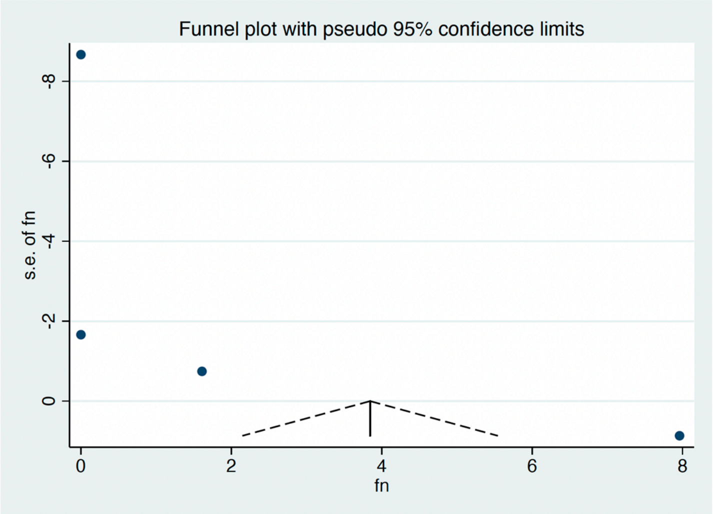
Studies assessing the association between post-stroke statin use and stroke recurrence after 1 year of ischemic stroke	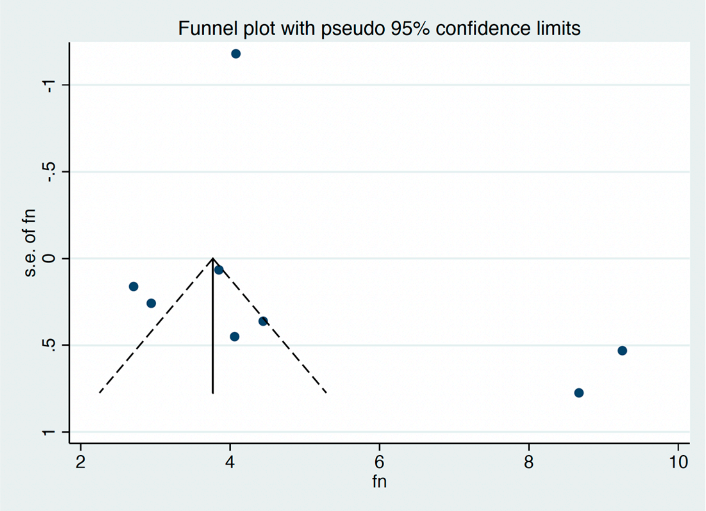

Studies assessing the association between post-stroke statin use and stroke recurrence within 1 year of cardioembolic or AF-related stroke	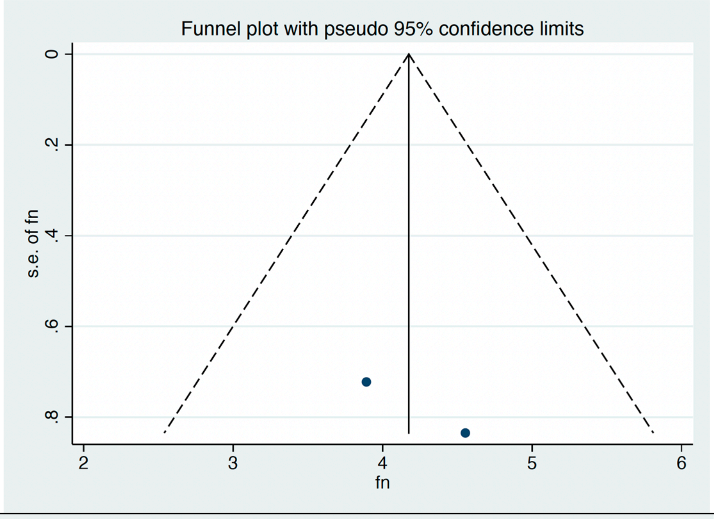
Studies assessing the association between post-stroke statin use and stroke recurrence after 1 year of cardioembolic or AF-related stroke	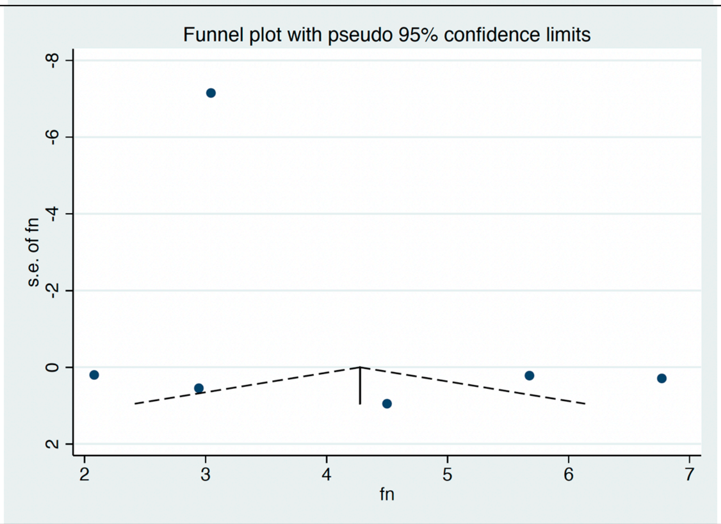
Studies assessing association between increasing statin intensity and all-cause mortality	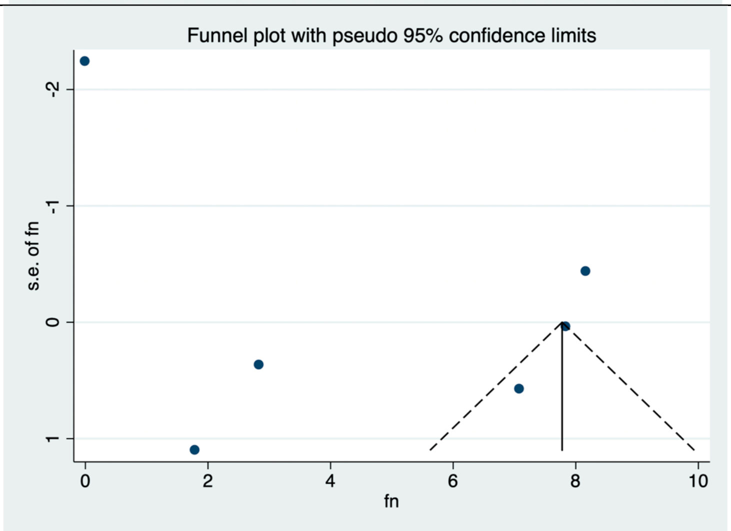

Studies assessing association between increasing statin intensity and stroke recurrence	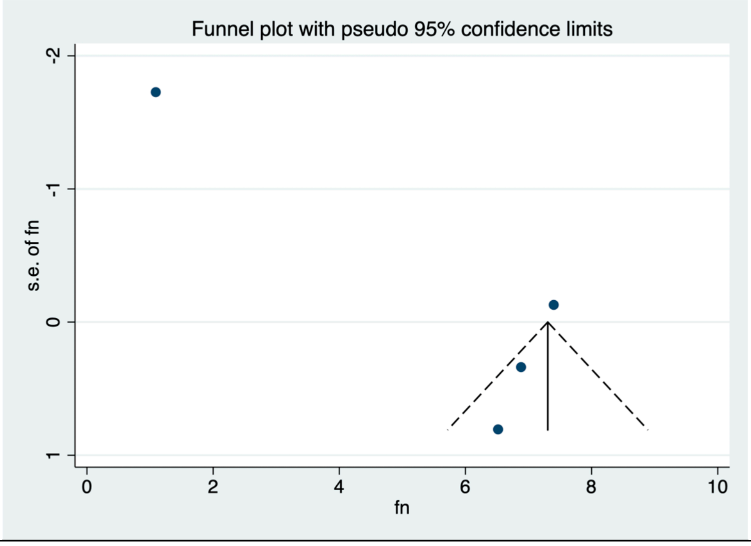

Abbreviations: SE, standard error; fn, false negative (refers to occurrence of adverse event in those taking statins); AF, atrial fibrillation
